# Genome-Wide Analysis and Characterization of the Proline-Rich Extensin-like Receptor Kinases (PERKs) Gene Family Reveals Their Role in Different Developmental Stages and Stress Conditions in Wheat (*Triticum aestivum* L.)

**DOI:** 10.3390/plants11040496

**Published:** 2022-02-11

**Authors:** Mahipal Singh Kesawat, Bhagwat Singh Kherawat, Anupama Singh, Prajjal Dey, Snehasish Routray, Chinmayee Mohapatra, Debanjana Saha, Chet Ram, Kadambot H. M. Siddique, Ajay Kumar, Ravi Gupta, Sang-Min Chung, Manu Kumar

**Affiliations:** 1 Department of Genetics and Plant Breeding, Faculty of Agriculture, Sri Sri University, Cuttack 754006, Odisha, India; mahipal.s@srisriuniversity.edu.in (M.S.K.); anupama.s@srisriuniversity.edu.in (A.S.); prajjal.d@srisriuniversity.edu.in (P.D.); 2 School of Biological Sciences and Institute for Molecular Biology and Genetics, Seoul National University, Seoul 08826, Korea; 3 Krishi Vigyan Kendra, Bikaner II, Swami Keshwanand Rajasthan Agricultural University, Bikaner 334603, Rajasthan, India; skherawat@gmail.com; 4 Department of Entomology and Plant Pathology, Faculty of Agriculture, Sri Sri University, Cuttack 754006, Odisha, India; snehasish.r@srisriuniversity.edu.in (S.R.); chinmayee.m@srisriuniversity.edu.in (C.M.); 5 Department of Biotechnology, Centurion University of Technology and Management, Bhubaneshwar 752050, Odisha, India; debanjana.saha@cutm.ac.in; 6 ICAR-Central Institute for Arid Horticulture, Bikaner 334006, Rajasthan, India; chetram.nbpgr@gmail.com; 7 The UWA Institute of Agriculture, The University of Western Australia, Perth, WA 6009, Australia; kadambot.siddique@uwa.edu.au; 8 Agriculture Research Organization, Volcani Center, Department of Postharvest Science, Rishon Lezzion 50250, Israel; ajaykumar_bhu@yahoo.com; 9 College of General Education, Kookmin University, Seoul 02707, Korea; ravigupta07@ymail.com; 10 Department of Life Science, Dongguk University, Dong-gu, Ilsan, Seoul 10326, Korea; smchung@dongguk.edu

**Keywords:** PERK, kinase, RT-qPCR, promoter, drought, heat stress

## Abstract

Proline-rich extensin-like receptor kinases (PERKs) are a class of receptor kinases implicated in multiple cellular processes in plants. However, there is a lack of information on the PERK gene family in wheat. Therefore, we identified 37 PERK genes in wheat to understand their role in various developmental processes and stress conditions. Phylogenetic analysis of PERK genes from *Arabidopsis thaliana*, *Oryza sativa*, *Glycine max,* and *T. aestivum* grouped them into eight well-defined classes. Furthermore, synteny analysis revealed 275 orthologous gene pairs in *B. distachyon*, *Ae. tauschii*, *T. dicoccoides*, *O. sativa* and *A. thaliana*. Ka/Ks values showed that most TaPERK genes, except TaPERK1, TaPERK2, TaPERK17, and TaPERK26, underwent strong purifying selection during evolutionary processes. Several cis-acting regulatory elements, essential for plant growth and development and the response to light, phytohormones, and diverse biotic and abiotic stresses, were predicted in the promoter regions of TaPERK genes. In addition, the expression profile of the TaPERK gene family revealed differential expression of TaPERK genes in various tissues and developmental stages. Furthermore, TaPERK gene expression was induced by various biotic and abiotic stresses. The RT-qPCR analysis also revealed similar results with slight variation. Therefore, this study’s outcome provides valuable information for elucidating the precise functions of TaPERK in developmental processes and diverse stress conditions in wheat.

## 1. Introduction

Protein phosphorylation is a key post-translational modification that regulates cell signaling networks and cellular processes in response to internal and external environmental stimulation through the reversible regulation of protein function by activation or deactivation, formation of protein complexes, and determination of the subcellular location of proteins [1,2,3]. Phosphorylation is the most common event in which the phosphoryl group transfers from adenosine triphosphate to hydroxyl residue of the protein substrate [4]. Although plants deploy receptor kinases at the cell surface to perceive, the signal, generated in the sudden changing environment, activates the various signaling pathways and regulates the growth, reproduction, and response against diverse stresses [2,5]. Receptor kinases are the most prominent gene family in crop species, including Arabidopsis, rice, maize, soybean, cotton, and sorghum [6,7,8]. For example, the Arabidopsis receptor kinase gene family encompasses ~610 members, and their homologs have been identified and characterized in many plant species [7,9,10,11]. The biological functions of these predicted receptor kinase genes remain to be elucidated. However, receptor kinases play a crucial roles in cell differentiation, pollen tube growth, pollen development, symbiosis, pathogen recognition, phytohormone response, signal transduction, self-incompatibility and response towards internal and external stimuli [8,9,12,13,14,15,16]. A few studies have identified and characterized the ligands that activate specific receptor kinases, as well as some signaling components [8,13]. Receptor kinases bind to different kinds of biomolecules such as polypeptides, steroids, carbohydrates, and cell wall components. These receptor kinases perceive and transduce the signals across the plasma membrane via diverse signaling complexes, which have been developed during the long course evolution of complex multicellular organisms [2,9].

Receptor kinases were divided into different groups based on the motifs structure in their extracellular domains [9,17]. For instance, the leucine-rich repeat receptor kinase family comprises the main class of receptor kinases in plants, including BAK1 and BRI1, which have been implicated in brassinosteroid signaling [18,19,20]. Proline-rich extensin-like receptor kinases (PERKs) are one of the main classes of receptor kinases. Fifteen PERK genes have been found in the Arabidopsis genome. However, their functions are poorly understood [6,12]. Most of the *AtPERK* gene family members are ubiquitously expressed, while few genes are specifically expressed [6]. For instance, *AtPERK1* is broadly expressed, whereas the expression of *AtPERK2* is mainly observed in rosette leaf veins, stems, and pollen [21]. In addition, the expression of *AtPERK8* and *AtPERK13* were detected in root hairs [6,22,23]. The expression of *AtPERK5*, *AtPERK6*, *AtPERK7*, *AtPERK11,* and *AtPERK12* were highly elevated in pollen. However, unnoticeable expressions were observed in the sporophytic tissues [23,24,25]. Furthermore, *AtPERK4* regulates the root growth function at an early stage of ABA signaling by perturbing calcium homeostasis in Arabidopsis [14]. A few studies have demonstrated that increased concentrations of calcium in the cells also enhances antioxidant enzyme activities and eventually regulates the lipid peroxidation of cell membranes and stomatal apertures [26,27,28]. The PERKs suppress the accumulation of reactive oxygen species (ROS) in the root, which is necessary for root hair growth [29,30]. MAPK cascade is an essential regulator of high light-induced Cu/Zn SODs and anti-PERK antibodies from animals, used to detect the presence of homologous proteins such as MPK3 and MPK6 in plants [30,31]. *AtPERK5* and *AtPERK12* are essential for the pollen tube growth in Arabidopsis [16]. Furthermore, *AtPERK8*, *AtPERK9*, and At*PERK10* negatively regulate root growth in Arabidopsis [23]. *PERK1* rapidly induces early perception and response to a wound in Chinese cabbage [12]. Antisense suppression of *BnPERK1* has exhibited various growth defects, such as amplified secondary branching, loss of apical dominance, and defects in floral organ formation. At the same time, the overexpression line showed increased lateral shoot production, seed set, and unusual deposition of callose and cellulose in *Brassica napus* [21]. A PERK-like receptor kinase specifically interacts with the nuclear shuttle protein (NSP), led viral infection, and positively regulates the NSP function in cabbage leaf curl virus and geminivirus [32].

Plants are sessile organisms, and constantly face fluctuating environmental conditions and various biotic and abiotic stresses during growth and development [33,34,35]. For example, wheat is an important cereal crop cultivated worldwide [36,37], and its quality and productivity are largely influenced by different biotic and abiotic factors [38,39]. With the recent advent of sequencing technology, a rapid increase in sequenced plant genomes has been accessed in the past few years [40]. However, identifying the genes in plant species’ genomes is now a great challenge, particularly in terms of their structure to functionally characterization [41,42]. For example, the wheat genome sequenced, completed and identified 124,201 genes [43]. Thus, this project’s completion has made it possible to complete genome-wide analysis and identification of the PERK gene family in wheat. We performed a comprehensive analysis of 37 PERK genes using several computational approaches in this work.

Furthermore, phylogenetic analysis, physical and biochemical properties, exon/intron, conserved motifs, chromosomal distribution, subcellular localization, gene duplication, Ka/Ks values, synteny analysis, and three-dimensional (3D) structure were also determined. In addition, tissue-specific expression profiles and responses to diverse stress conditions were also examined for the TaPERK genes. The outcome of the present study will be helpful in the detailed understanding of the TaPERK gene’s role in plant growth, development, and survivability under different stress conditions.

## 2. Results

### 2.1. Identification of TaPERK in Wheat

In this study, we identified 37 PERK genes in the wheat genome using various computational approaches (Table 1).

This number is relatively higher than the earlier reported PERK genes in Arabidopsis, soybean, rice, sorghum, maize, and cotton (Table 2).

This might be due to the higher chromosome number and big size of the wheat genome, which indicates that the PERK genes underwent a substantial expansion in wheat. In addition, wheat is derived from the hybridization of three progenitor genomes: A, B, and D. The TaPERK family had protein lengths ranging from 401–1052, and amino acid with molecular weight (MW) 44.37–113.73 kDa for TaPERK23 and TaPERK7, respectively. The isoelectric point (pI) ranged from 5.22 and 9.04 for TaPERK34 and TaPERK3, respectively. We also plotted the MW of TaPERK with their pI to understand the MW distribution of different TaPERK proteins (Figure S1). The plots showed that most of the TaPERKs had similar MW and pI. Hence, pI values ranged from acidic to basic, and the heaviest TaPERK was over twice the weight of the lightest. Furthermore, the grand average of hydropathy index values ranged from −0.09 to −0.645, indicating that TaPERK proteins are hydrophilic in nature. Moreover, the subcellular localization prediction of TaPERK proteins indicated that most of the TaPERKs were situated on the plasma membrane (Table 1).

**Figure 1:**
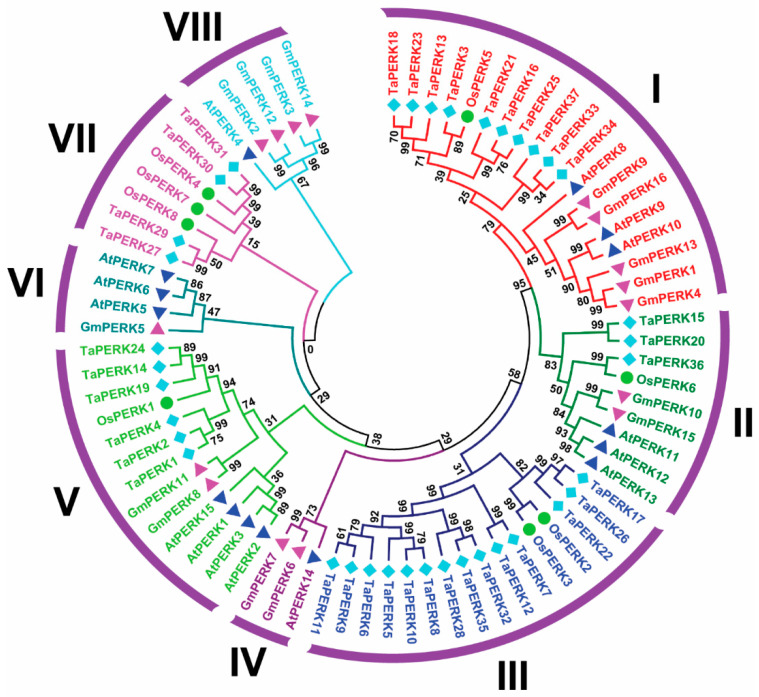
Phylogenetic analysis of TaPERK proteins with Arabidopsis (15), rice (8), and soybean (16). The phylogenetic analysis was executed using the ClustalW program as well as MEGAX software by the neighbor-joining method and bootstrap values of 1000 replicates. The numbers on the nodes indicate the bootstrap values. Distinct groups are represented by the different colors.

To understand the origins and evolutionary dynamics between plant species PERKs, the phylogenetic tree was produced with TaPERKs, AtPERKs OsPERKs, and GmPERK proteins (Table S2). The phylogenetic analysis revealed that TaPERK proteins were classified into eight groups (Figure 1).

Group III was the biggest with 14 members, while Group I, II, IV, V, VI, VII, and VIII contained 10, 3, 0, 6, 0, 4, and 0 members, respectively (Figure S2).

### 2.2. Chromosomal Distribution, Gene Duplication, and Synteny Analysis

**Figure 2:**
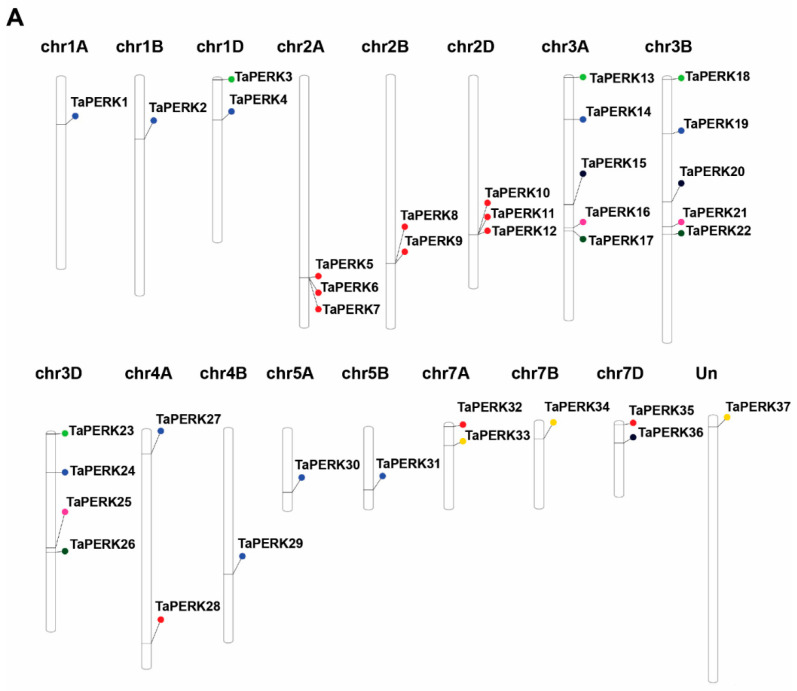

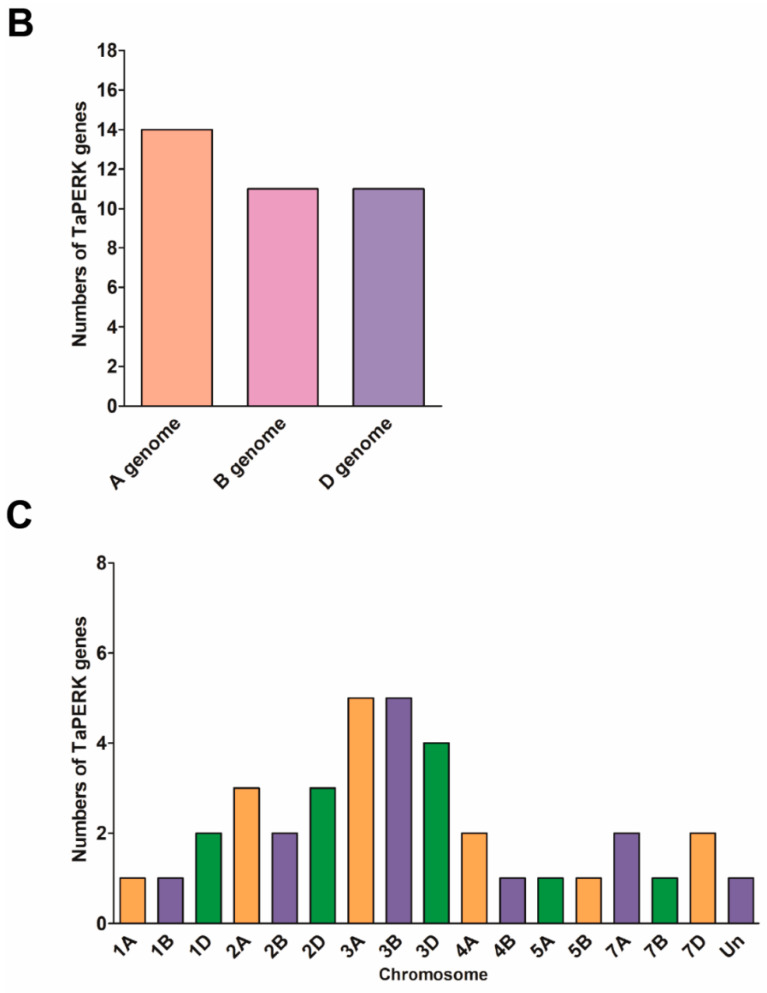
Genomic distribution of identified PERK genes on the 21 chromosomes of wheat and within the three sub-genomes. (**A**) Schematic representations of the chromosomal distribution of PERK genes on the 21 chromosomes of wheat and the name of the gene on the right. The colored circles on the chromosomes indicate the position of the PERK genes. The chromosome numbers of the three sub-genomes are indicated at the top of each bar. (**B**) Distribution of PERK genes in the three sub-genomes. (**C**) Distribution of PERK genes across 21 chromosomes, Un: unaligned contig.

To map the chromosomal distribution of the identified TaPERK genes in wheat, corresponding to chromosomal locations of PERK genes were determined using the PhenGram online server. The TaPERK genes were found on 17 wheat chromosomes (Figure 2A and Table 1). TaPERK genes showed a higher presence on A sub-genomes (Figure 2B). Maximum TaPERK genes (Fourteen) were located on the chromosomes of the A sub-genome.

The B and D sub-genome had a minimum number of TaPERK genes (Eleven). Five TaPERKs were mapped on chromosomes 3A and 3B (Figure 2C). The lowest number of TaPERKs was detected on the chromosomes 1A, 1B, 4B, 5A, 5B, and 7B (single gene, respectively). On the contrary, none of the TaPERK genes were located on the chromosomes 4D, 5D, and 6D. In addition, one TaPERK was located on an unaligned contig. Thus, all the PERK family members were uniformly distributed on the wheat’s A, B, and D sub-genome.

To explore why the wheat was polyploidy with the largest genome, we further investigated the duplication events in the TaPERK gene family. The phylogenetic analysis of the TaPERK genes also revealed many duplication events (Figure S3). We observed that 26 PERK genes in wheat involved duplication events (Figure S4 and Table S3), indicating expanding the PERK gene family in wheat. Furthermore, to examine the selective pressure on the duplicated TaPERK genes, we analyzed the synonymous substitution (Ks), non-synonymous (Ka), and the Ka/Ks ratios for the 13 TaPERK genes pairs (Table S3). The value of Ka/Ks = 1 indicates that genes underwent a neutral selection; <1 denotes negative selection or purifying, and >1 suggests a positive selection [44]. The Ka/Ks values for all 11 gene pairs were <1, which indicates that TaPERK genes experienced a robust purifying selection pressure with slight alteration after duplication. However, 2 gene pairs, *TaPERK1*/*TaPERK2* and *TaPERK17*/*TaPERK26,* had more than 1, which suggests that two pairs of TaPERK genes experienced a positive selection (Table S3). These findings showed the conserved evolution of TaPERKs.

**Figure 3:**
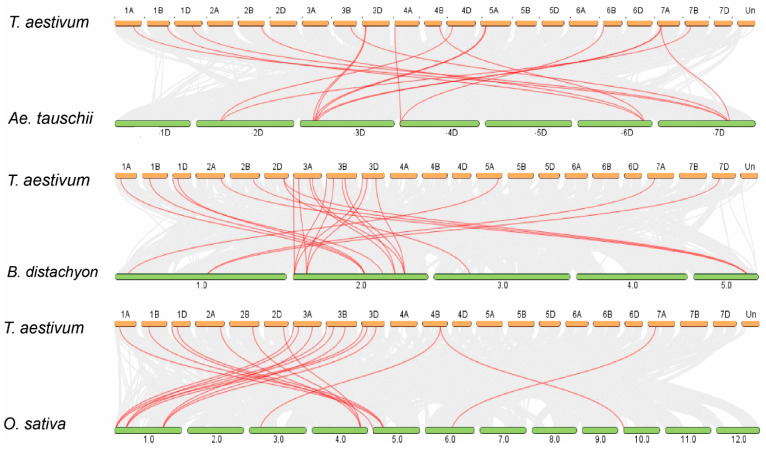
Syntenic relationships of TaPERK genes between *Aegilops tauschii*, *Brachypodium distachyon*, and *Oryza sativa*. The gray lines in the background represent the collinear blocks within *Triticum aestivum* and other plant genomes, while the red lines highlight the syntenic PERK gene pairs.

To further elucidate the synteny relationships of TaPERK genes with wheat relatives and other model plants, including *B. distachyon*, *Ae. tauschii*, *T. dicoccoides*, *O. sativa,* and *A. thaliana*, multiple collinearity scan tools were run to identify the orthologous genes between genomes of these plant species (Figure 3 and Table S4).

We found 35, 30, 61, 66, and 83 orthologous gene pairs between TaPERKs with other PERK genes in *B. distachyon*, *Ae. tauschii*, *T. dicoccoides*, *O. sativa,* and *A. thaliana*, respectively. The results showed that 26, 24, 37, 42, and 66 TaPERK genes were collinear with PERK genes in *B. distachyon*, *Ae. tauschii*, *T. dicoccoides*, *O. sativa,* and *A. thaliana*, respectively. Some of the TaPERK genes had five pairs of orthologous genes, for example; *TaPERK5*, *TaPERK6*, *TaPERK8*, *TaPERK9*, and *TaPERK11*, while few of the TaPERK genes had four pairs of orthologous genes, *TaPERK7*, *TaPERK10*, *TaPERK12*, *TaPERK15*, *TaPERK20*, *TaPERK28*, *TaPERK32* and *TaPERK35* that might have played an essential role in the evolution of PERK genes. Thus, these results indicated that PERK genes in wheat-derived from a common ancestor.

### 2.3. Exon/Intron Structure and Motif analysis of TaPERK Genes

**Figure 4:**
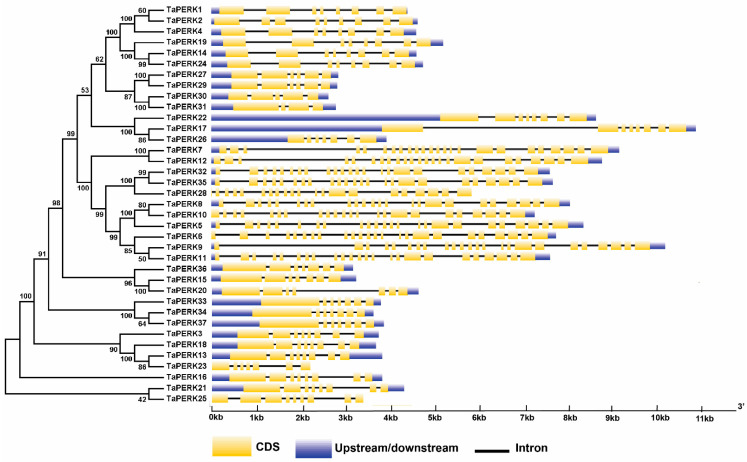
Diagrammatic representation of the exon–intron organization of the TaPERK genes. Yellow boxes represent exons, untranslated regions (UTRs) are indicated by blue boxes, and black lines represent introns. The lengths of the boxes and lines are scaled based on gene length. The exon and intron sizes can be estimated using the scale at the bottom.

To elucidate the structural character of the TaPERK genes, the exon/intron organization and conserved motifs (Figure 4) of TaPERK genes were examined.

**Figure 5:**
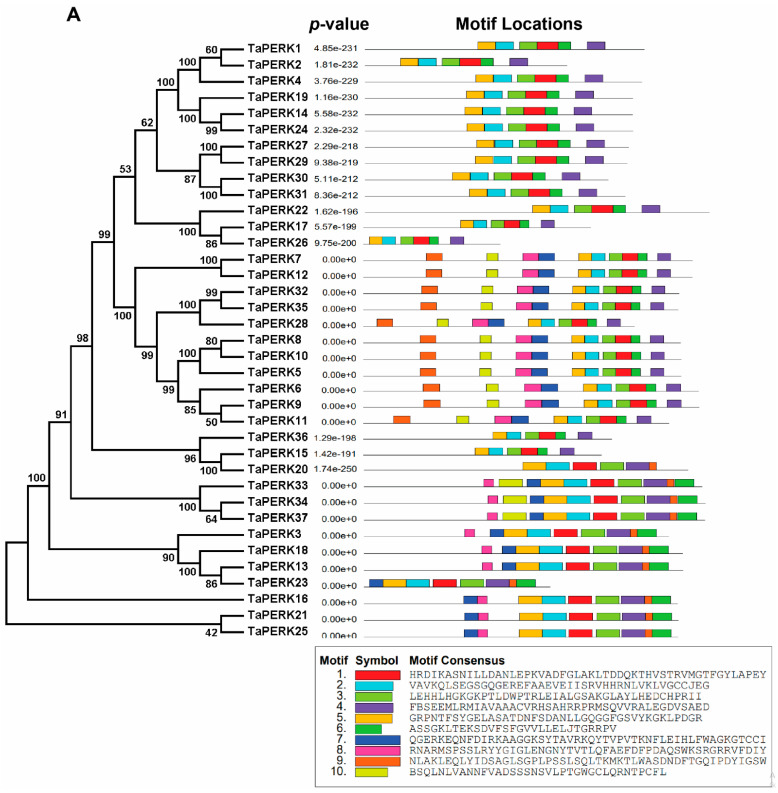

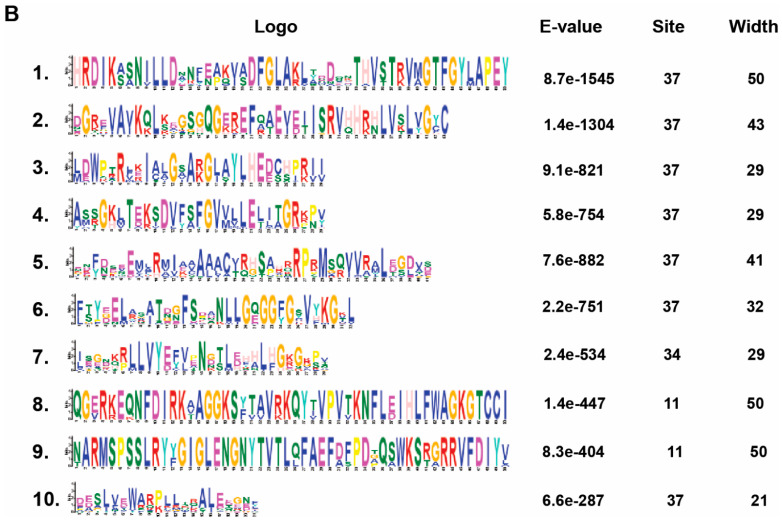
Conserved motifs of TaPERK genes elucidated by MEME. Up to 10 motifs were shown in different colors. (**A**) Colored boxes representing different conserved motifs with different sequences and sizes. (**B**) Sequence logo conserved motif of the wheat PERK proteins. The overall height of each stack represents the degree of conservation at this position, while the height of individual letters within each stack indicates the relative frequency of the corresponding amino acids. The sequence of each motif, combined *p*-value, and length are shown on the left side of the figure. MEME Parameters: number of repetitions, any; maximum number of motifs, 10; optimum motif width, between 6 and 50.

Exon–intron analysis showed that the TaPERK gene family greatly varied in terms of gene structure. For instance, most TaPERK genes contain 3–23 introns. Maximum twenty-three introns were detected in the *TaPERK5*, *TaPERK6*, *TaPERK7*, *TaPERK8*, *TaPERK9*, *TaPERK11*, *TaPERK12*, *TaPERK32,* and *TaPERK35*, while *TaPERK31* had three introns (Figure S5). Furthermore, we also analyzed the conserved motif of TaPERK genes using the Multiple Em for Motif Elicitation (MEME) webserver. Eventually, ten well-preserved motifs were found in 37 TaPERK genes (Figure 5A,B).

**Figure 6:**
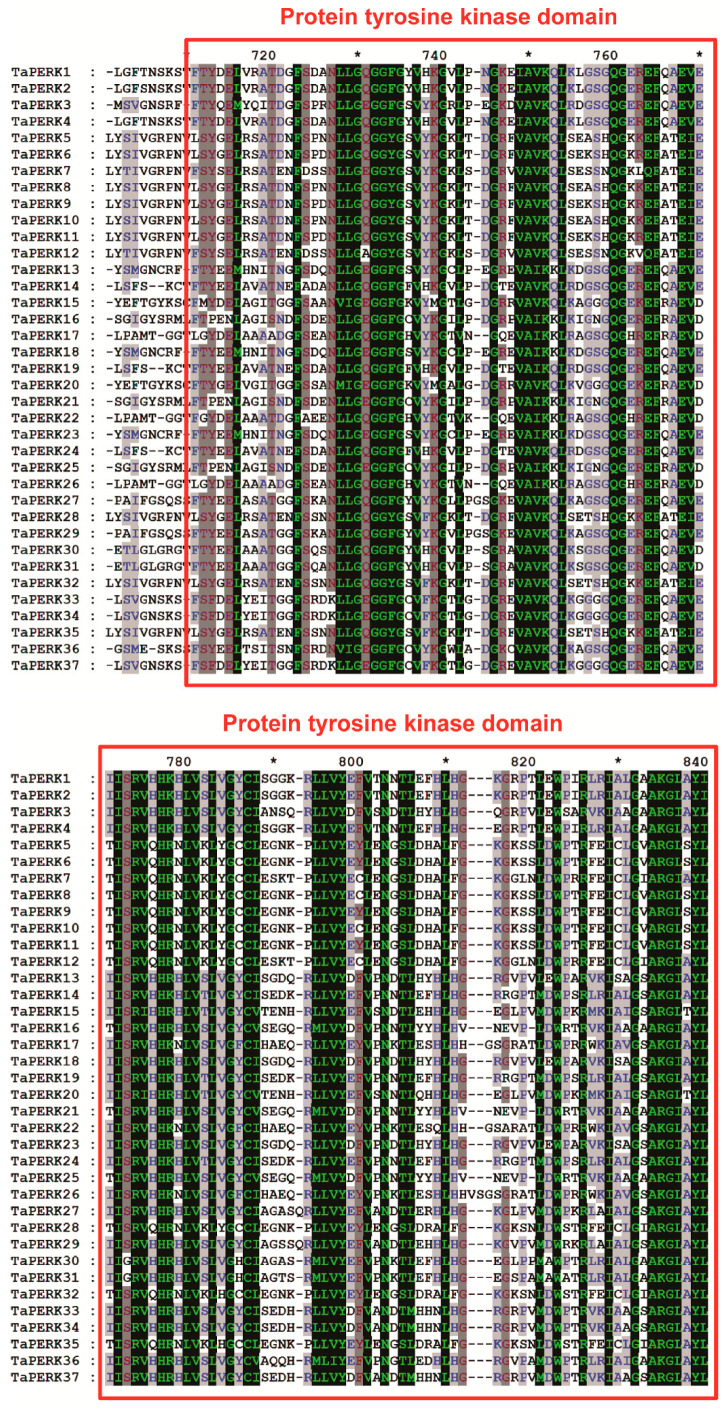

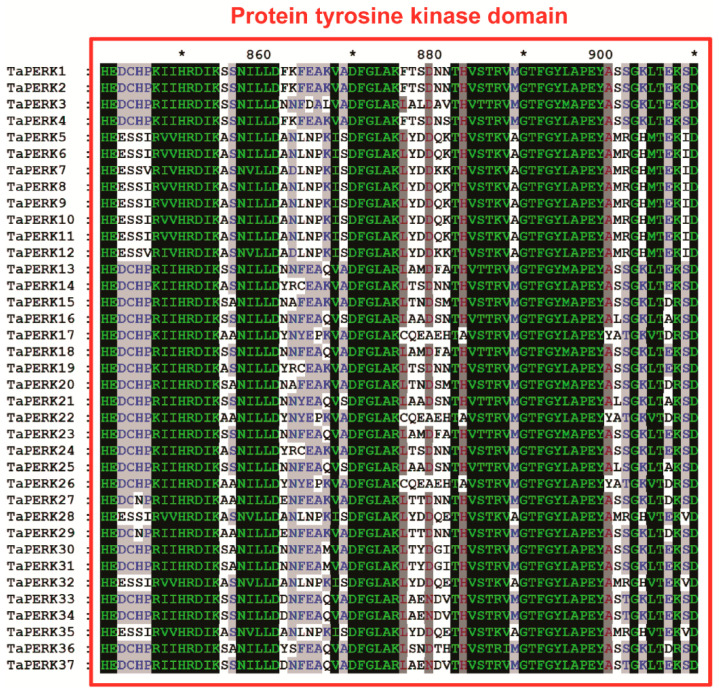
Multiple sequence alignment of the TaPERK protein sequences. The conserved protein tyrosine kinase domain is boxed in red. Colored and shaded amino acids are chemically similar residues. Dashes indicate gaps introduced to maximize the alignment of the homologous region. * indicates positions which have a single, fully conserved residue.

Furthermore, the TaPERK gene family was detected by the presence of the tyrosine kinase domain (Pfam PF07714), and all TaPERKs consist of at least one tyrosine kinase domain (Table S5) involved in signal transduction. Furthermore, to understand the biological function of TaPERK genes in wheat, 3D protein models of all TaPERKs were produced using a phyre2 webserver. TaPERKs 3D protein structure had two distinct subdomains, a smaller N-terminal lobe and a bigger C-terminal lobe connected by a small hinge loop (Figure S6B). In addition, protein sequence alignment also showed that all TaPERK proteins consisted of a conserved tyrosine kinase domain (Figure 6 and Figure S6A).

This result will help understand and explain the substrate specificity and molecular function of TaPERK genes in activating the PERK signal transduction pathway.

### 2.4. Cis-Acting Regulatory Elements (CAREs) Analysis of TaPERK Genes

**Figure 7:**
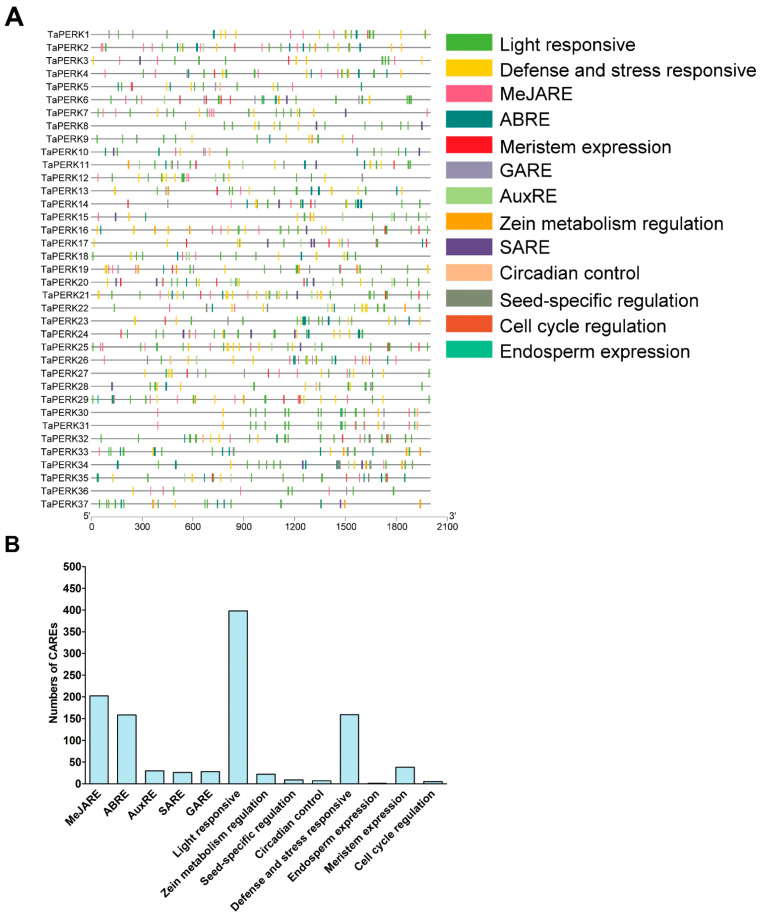
Cis-acting regulatory elements (CAREs) in the promoter region of the TaPERK genes family. The CAREs analysis was performed with a 2kb upstream region using PlantCARE online server. The different numbers of *cis*-regulatory elements represent different colors. (**A**) Hormone-responsive elements, stress-responsive elements, growth and development-related elements, light-responsive elements, and other elements with unknown functions are differentiated by color. (**B**) Most commonly occurring CAREs in TaPERKs.

To further understand the function of TaPERK genes, upstream 2000 bp sequences from the transcription start site of TaPERKs were analyzed using the PlantCARE web server. This analysis revealed that the promoter region of TaPERKs gene families contained the multiple *cis*-elements related to phytohormones, developmental processes, and different stresses (Figure 7A and Table S6).

The TaPERKs gene family consists of five hormone response elements, including the auxin response element (AuxRE), gibberellin response element (GARE), methyl jasmonate response element (MeJARE), abscisic acid response element (ABRE), and salicylic acid response element (SARE). The response elements belong to light responses, MeJARE, defense, and stress response and ABRE were the most abundant CAREs in the TaPERK gene family (Figure 7B). This result indicates that TaPERKs play a crucial role in plant growth and development.

Furthermore, TaPERKs contain *cis*-elements related to zein metabolism, endosperm expression, circadian control, meristem expression, seed-specific, and cell cycle regulation. Thus, the CAREs found in the TaPERK gene family indicate that TaPERKs might be participating in a wide range of biological processes. Furthermore, various types of CAREs in the TaPERK genes suggest that these genes might be involved in diverse developmental processes. Therefore, these results provide valuable insights to understand the regulatory mechanism of the TaPERK gene family in response to phytohormone, defense, stress, and various developmental processes.

### 2.5. Gene Ontology (GO) Enrichment of TaPERK Genes

Gene ontology (GO) assists in understanding the biological function of any genes by comparing their sequence similarity with the known function of genes and gene products with other species [40,42]. All TaPERKs were successfully annotated and allotted GO terms using AgriGO, and further verified using eggNOG-Mapper (Figure S7; Table S7 and Table S8), giving almost the same results as AgriGO. In the biological process category, TaPERK genes were enriched in cell communication (GO:0007154), signaling (GO:0023052), cellular process (GO:0009987), and regulation of biological process (GO:0050789) categories (Figure S7A). In the cellular component category, TaPERK displayed enrichment in the cell (GO:0005623), cell junction (GO:0030054), and membrane (GO:0016020) (Figure S7B). Furthermore, subcellular localization prediction (Table 1) also provided indistinguishable results. In the molecular function category, molecular transducer activity (GO:0060089) and catalytic activity (GO:0003824) were the most prevalent category which was primarily involved in signal transduction (Figure S7C). Apart from cell communication and signaling, the GO term analysis also suggested a variety of roles of TaPERK genes, such as maintenance of dormancy, tissue development, organ formation, post-embryonic organ development, gametophyte development, seedling development, and regulation of developmental process and metabolism. Thus, these results demonstrate that TaPERK genes play a critical role in plant growth and development.

### 2.6. Expression Profiling of TaPERK Genes in Various Developmental Stages and under Diverse Stress Conditions

**Figure 8:**
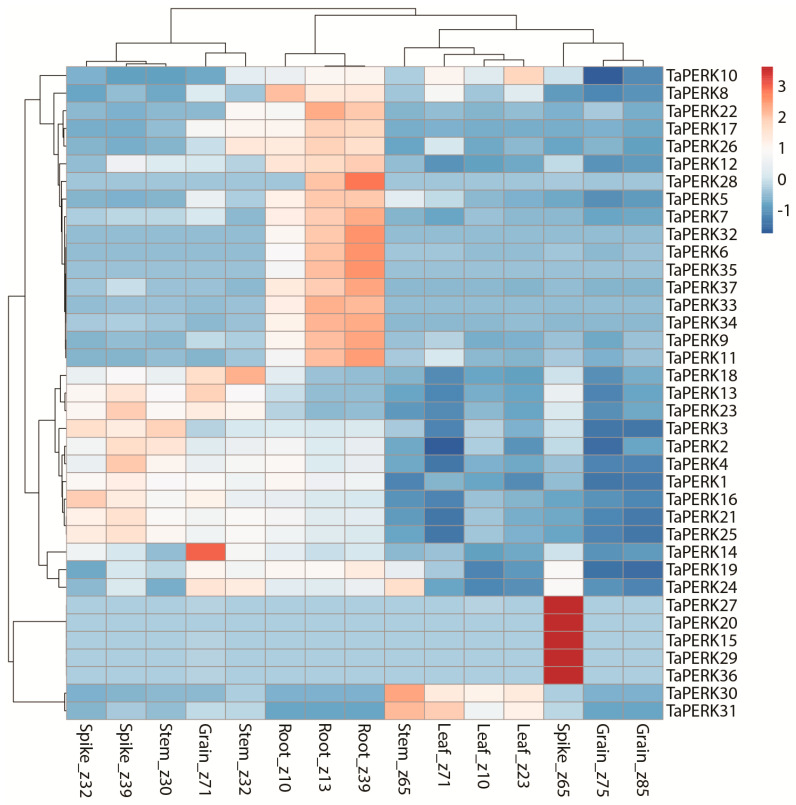
Heatmap representing expression profile of the TaPERK genes at various developmental stages. Columns represent genes, and rows represent different developmental stages. TPM values were used directly to create the heatmaps. The “z” nomenclature refers to Zadok’s growth stage.

**Figure 9:**
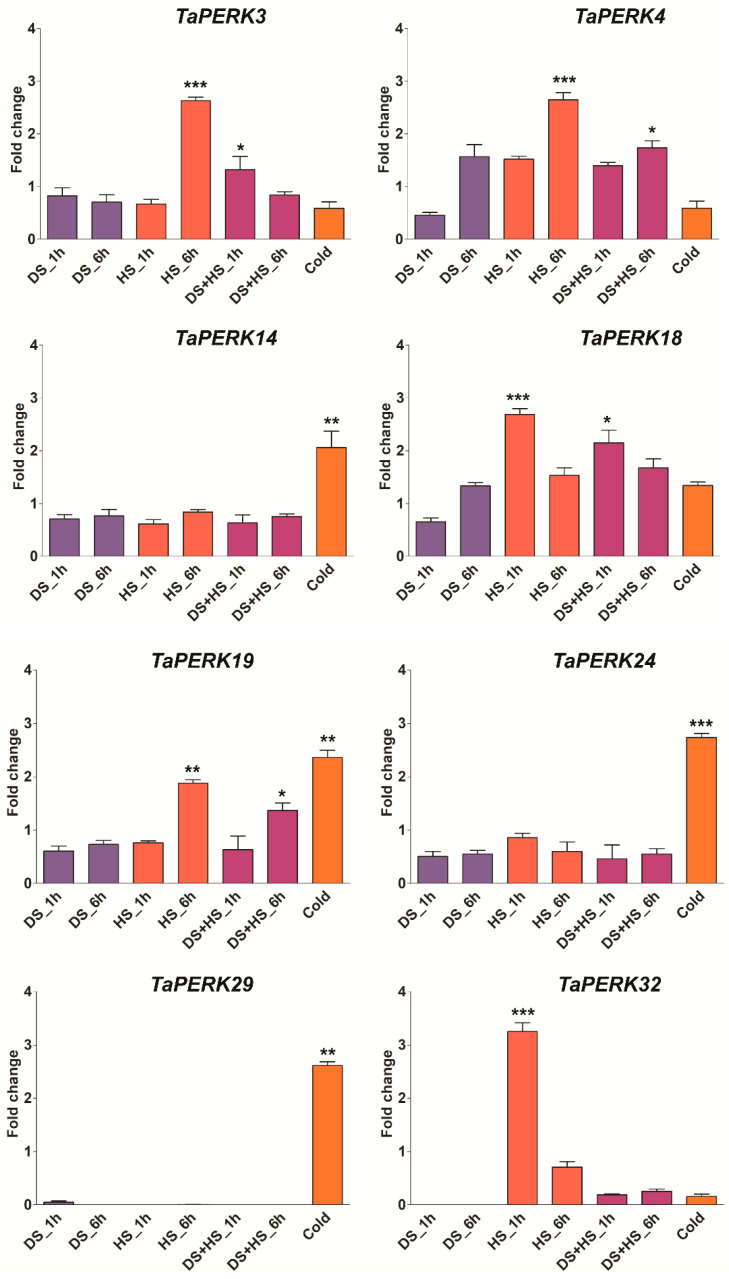
Quantitative real-time PCR analysis of selected TaPERK genes in response to drought stress (DS), heat stress (HS), and cold stress to verify RNA seq data. The wheat actin gene was used as the internal control to standardize the RNA samples for each reaction. Asterisks indicate significant differences compared with control. Bars represent results of Tukey’s HSD test at the <0.05 and <0.001 level (* *p* < 0.05, ** *p* lies in between the values of 0.05 and 0.001, and *** *p* < 0.001). Error bars show standard deviation. Data are mean ± SD (*n* = 3).

To investigate the precise function of TaPERK genes, the expression pattern of TaPERK genes was examined during different developmental and in diverse stress conditions. The TPM values of all TaPERKs were retrieved from the wheat gene expression database. These TPM values were directly used to generate the PCA and heatmaps (Figure S8A,B, Figure 8 and Figure 9).

To examine the expression pattern of TaPERKs, five tissues from three different developmental stages were taken in this work. The TaPERK genes displayed differential induction among the different tissues; for example, *TaPERK2*, *TaPERK4*, *TaPERK13*, *TaPERK21*, *TaPERK23,* and *TaPERK25* exhibited induction at the spike z39 stage, while *TaPERK15*, *TaPERK20*, *TaPERK27*, *TaPERK29,* and *TaPERK36* exhibited induction at spike z65 stage (Figure 8).

The expression of TaPERK5, TaPERK6, TaPERK7, TaPERK8, TaPERK9, TaPERK11, TaPERK12, TaPERK17, TaPERK22, TaPERK26, TaPERK28, TaPERK32, TaPERK33, TaPERK34, TaPERK35, and TaPERK37 were elevated in roots at z13 and z39 stage, respectively. TaPERK10, TaPERK30, and TaPERK31 were also up-regulated in the leaf at the z23 and z71 stages, respectively. In addition, TaPERK13, TaPERK14, TaPERK23, TaPERK24, and TaPERK31 showed induction at the grain z71 stage. TaPERK18 and TaPERK24 showed higher expression at the stem z30 stage, whereas TaPERK24, TaPERK30, and TaPERK31 expression was raised at the stem z65 stage (Figure 8). These results showed that the TaPERK gene family members might be involved in developing different tissues and stages.

Expression patterns of TaPERKs were also investigated under the different stress conditions, including septoria tritici blotch (STB), stripe rust, powdery mildew, drought, and heat stress. The expression of several members of the TaPERK gene family was elevated in biotic and abiotic stress (Figure S9). The expression of *TaPERK9*, *TaPERK13*, *TaPERK15*, *TaPERK17*, *TaPERK20*, *TaPERK22*, *TaPERK23*, *TaPERK26*, *TaPERK28*, *TaPERK33*, *TaPERK35,* and *TaPERK36* were induced during the septoria tritici blotch, while the expression of *TaPERK2*, *TaPERK6*, *TaPERK7*, *TaPERK8*, *TaPERK10*, *TaPERK11*, *TaPERK12 TaPERK27*, *TaPERK35,* and *TaPERK37* were significantly raised upon the powdery mildew infection. *TaPERK1*, *TaPERK8*, *TaPERK16*, *TaPERK21*, *TaPERK24*, *TaPERK25*, *TaPERK29*, *TaPERK30*, *TaPERK31,* and *TaPERK34* were highly elevated during the stripe rust infection. In the case of abiotic stress, the expression profile indicates the expression of a few members of the TaPERK family, for instance, *TaPERK3*, *TaPERK4*, *TaPERK18*, and *TaPERK32* were raised during the initial hours of heat stress. It seems that the TaPERK family does not participate in drought stress. However, only *TaPERK4* and *TaPERK18* genes were elevated during the combined drought and heat stress (Figure S9). The expression level of *TaPERK14*, *TaPERK19*, *TaPERK24,* and *TaPERK29* was significantly raised during cold stress. Furthermore, the expression patterns of a few selected TaPERK genes were validated through RT-qPCR, and the results displayed nearly similar expression patterns (Figure 9). Overall, these results demonstrated that different TaPERK genes respond to diverse stress conditions.

### 2.7. Protein–Protein Network Analysis of the TaPERK Family Genes

**Figure 10:**
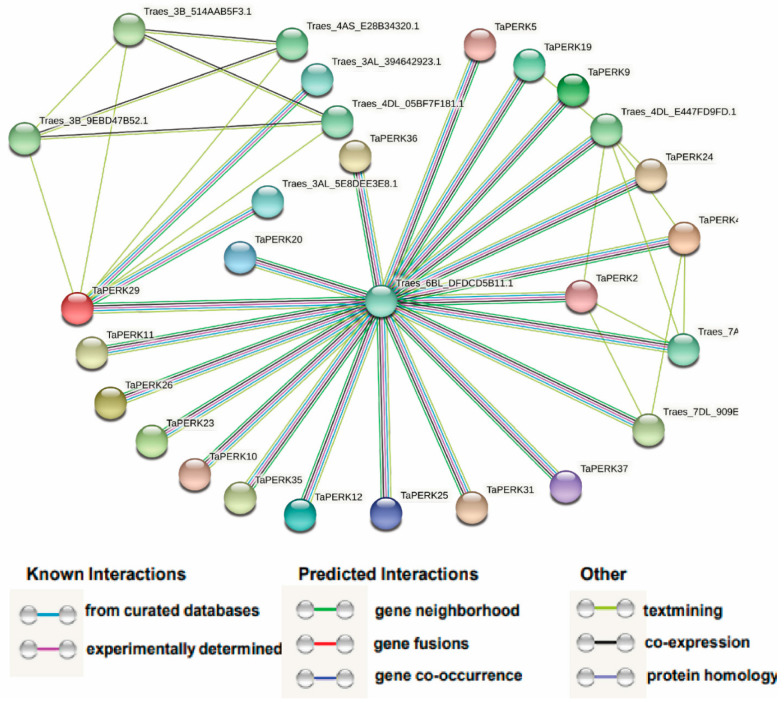
Protein–protein interaction analysis of TaPERKs proteins. Protein–protein interaction network produced by STRINGV9.1, each node represents a protein, and each edge represents an interaction, colored by evidence type. The figure highlights the connections between differentially represented proteins.

A protein network was produced using the STRING online webserver to examine the interactions between TaPERKs and other *T. aestivum* proteins (Figure 10 and Table S9).

We found eighteen TaPERKs interacting with 10 different wheat proteins according to the STRING results. TaPERK29 can interact with seven other wheat proteins (Traes_3AL_394642923.1, Traes_3AL_5E8DEE3E8.1, Traes_3B_514AAB5F3.1, Traes_3B_9EBD47B52.1, Traes_4AS_E28B34320.1, Traes_6BL_DFDCD5B11.1 and Traes_4DL_05BF7F181.1), which were cGMP-dependent protein kinase/PKG II, protein of unknown function (DUF1645) and BRASSINOSTEROID INSENSITIVE 1, and play critical roles in the Brassinosteroids signaling. TaPERK2 and TaPERK4 can interact with four other wheat proteins (Traes_7AL_5E0DD589E.1, Traes_4DL_E447FD9FD.1, Traes_6BL_DFDCD5B11.1 and Traes_7DL_909EA97B3.1) which were cGMP-dependent protein kinase/PKG II and non-specific serine/threonine-protein kinase. cGMP-dependent protein-kinase is a phosphorylated diverse biologically important pathway [45,46,47]. PKG is activated by cGMP and has been implicated in the regulation of cell division, nucleic acid synthesis response to biotic stress, stomata closure during osmotic stress, and development of adventitious roots [45,46,47,48,49]. These results provide important insight for further elucidating the complex biological functions of TaPERK genes.

## 3. Discussion

PERKs are a class of receptor kinases that have been implicated during various stages of growth and developments in plants, including cell differentiation, pollen tube growth, pollen development, symbiosis, pathogen recognition, phytohormone response, signal transduction, self-incompatibility, and response to internal and external stimuli [8,9,12,13,14,15,16]. PERKS gene family members have also been identified in other plant species, such as 15 genes in Arabidopsis, 8 in *O. sativa*, 23 in *Z. mays*, 16 in *G. max*, 15 in *S. bicolor*, 15 in *G. arboreum*, 16 in *G. raimondii*, and 33 from *G. hirsutum* [6,7,12]. However, this is the first time we have identified the PERK gene family in the wheat genome. Many studies have reported PERKS genes in ancient land plants, which have expanded during evolutionary processes [6,7,15]. However, in this study, we identified 37 TaPERK genes in the wheat genome (Table 1), which, upon phylogenetic analysis, classified the TaPERK gene family into eight subfamilies or groups (Figure 1). Phylogenetic analysis revealed that groups III and VII were monocot-specific TaPERKs, while groups IV, VI, and VIII contained dicot-specific TaPERKs (Figure 1). The evolution of this type of gene indicates the monocot’s specific functions that might play an essential role in establishing physiological and morphological development [40,42,50]. Although, TaPERK genes were distributed into the well-known rice, Arabidopsis, and the soybean cluster, indicating that TaPERKs might be derived from a common ancestor. In addition, most of the TaPERKs showed orthologous relationships with rice, Arabidopsis, and soybean PERKs.

Furthermore, the phylogenetic tree also displayed that all subfamilies have an expanded number of members (Figure 1 and Figure S2), suggesting that the duplication of TaPERKs results from a long course of evolution. Similar results were reported in Arabidopsis, *B. rapa,* and cotton [6,7,15]. Collectively, these results demonstrated a lineage-specific expansion of TaPERKs via the partial alteration of the genome to adapt to internal and external environments during evolution [40,42,50,51].

The wheat PERKS gene family was widely expanded and had comparatively more PERKs than the previously reported PERKs in *A. thaliana, O. sativa*, *G. max*, *S. bicolor*, *Z. mays*, *G. max*, *G. arboreum*, *G. raimondii,* and *G. hirsutum* [6,7,12]. Many previous studies have demonstrated that polyploidy enabled numerous plant species to adapt to adverse environmental conditions [44,52,53]. Mostly, polyploidy is linked with gene duplication and, in our study, we also found that tandem, segmental and whole-genome duplication was the critical driving force responsible for the duplication of TaPERK genes. Segmental duplication is the fundamental drive factor, and occurs in numerous plant genomes during evolution consisting of several duplicated chromosomal blocks [54]. For instance, several Arabidopsis gene families experienced coherent evolutionary dynamics directed to expanding the gene family [55,56]. Moreover, several gene families, including cotton GRAS, RH2FE3, MADS-Box, MIKC-Type, YABBY, WOX, sesame heat shock proteins, and soybean WRKY, experienced segmental expansion and whole-genome duplication events [7,57,58,59,60,61,62]. The chromosomal map of TaPERK genes revealed that the 37 TaPERKs were unequally distributed throughout chromosomes, excluding chromosome 6 (Figure 2). The gene number on each chromosome varied from one to five: chromosomes 3A and 3B had five genes; chromosome 3D had four genes; chromosome 2A and 2D contained three genes; chromosomes 1D, 2B, 4A,7A and 7D had two genes; and 1A, 1B, 4B, 5A, 5B and 7B consisted of a single gene. Hence, uneven distribution of the TaPERK genes on the 17 chromosomes of wheat indicates probable gene addition or loss via whole genome or segmental duplication events and errors during genome sequencing and assembly. Gene duplication analysis showed 13 pairs of duplicated genes, which shared high sequence similarity at the nucleotide level. The duplicated pairs were *TaPERK1:TaPERK2*, *TaPERK14:TaPERK24*, *TaPERK27:TaPERK29*, *TaPERK30:TaPERK31*, *TaPERK17:TaPERK26*, *TaPERK7:TaPERK12*, *TaPERK32:TaPERK35*, *TaPERK8:TaPERK10*, *TaPERK9:TaPERK11*, *TaPERK15:TaPERK20*, *TaPERK34:TaPERK37*, *TaPERK13:TaPERK23*, and *TaPERK16:TaPERK21*. Furthermore, the Ka/Ks value of 11 gene pairs was <1, indicating that TaPERK genes experienced a robust purifying selection pressure (Figure S3 and Table S3). However, two gene pairs, *TaPERK1*:*TaPERK2* and *TaPERK17*:*TaPERK26,* had more than 1, suggesting that two pairs of TaPERK genes underwent a positive selection. Therefore, these results indicate that TaPERK genes were not changed much in function after duplication and exhibited the conserved evolution of TaPERK genes. Qanmber and colleagues (2019) also reported similar results in cotton. Furthermore, ten gene pairs were the results of segmental duplications in cotton [7]. Furthermore, 146 out of 149 duplicated gene pairs had a Ka/Ks ratio of <1.0, and only three duplicated gene pairs displayed more than 1, which indicates the positive selection pressure. Similar type gene duplication events were also described in the BrPERKs genes [15]. Our gene duplication analysis also demonstrated that the TaPERK gene duplication events were similar, as previously reported in the cotton and *Brassica rapa* [7,15]. Thus, these results showed that segmental and whole-genome duplications might play a critical role in the evolution and expansion of the PERK genes in wheat.

To further elucidate the synteny relationships of TaPERK genes with wheat relatives and other model plants, we identified 35, 30, 61, 66, and 83 orthologous gene pairs between TaPERKs with other PERK genes in *B. distachyon*, *Ae. tauschii*, *T. dicoccoides*, *O. sativa,* and *A. thaliana*, respectively (Figure 3 and Table S4). Additionally, *Ae. speltoides* (BB, diploid) and *Ae. tauschii* (DD, diploid) were the foundation of B and D subgenomes of wheat. The synteny relationship displayed that nine orthologous gene pairs between *Ae. tauschii* with a wheat D subgenome were found on the same chromosomes with two on 1D, one on 2D, four on 3D, and two on 7D (Figure 3 and Table S4). Furthermore, twenty-two orthologous gene pairs between *T. dicoccoides* with a wheat AABB subgenome were detected on the same chromosomes with one on 1A, two on 2A, five on 3A, two on 4A, one on 5A, two on 7A, one on 1B, one on 2B, four on 3B, one on 4B, one on 5B, and one on 7B (Figure 3 and Table S4). These findings suggest that PERK genes might have come from *Ae. tauschii* and *T. dicoccoides* during natural hybridization events. Furthermore, more orthologous gene pairs were found in *T. aestivum* with *A. thaliana* and *O. sativa*, which exhibited that TaPERK and other PERKS genes might be derived from these orthologous genes during evolution.

The gene structure analysis of TaPERKs revealed that TaPERKs greatly varied in gene structure. The majority of the TaPERK genes contained more than five exons, except for TaPERK31 with four exons, while TaPERK30 had five exons (Figure 4). A maximum of twenty-four exons were detected in *TaPERK5*, *TaPERK6*, *TaPERK7*, *TaPERK8*, *TaPERK9*, *TaPERK11*, *TaPERK12*, *TaPERK32*, and *TaPERK35*. Furthermore, a maximum of twenty-three introns were found in *TaPERK5*, *TaPERK6*, *TaPERK7*, *TaPERK8*, *TaPERK9*, *TaPERK11*, *TaPERK12*, *TaPERK32*, and *TaPERK35*, while *TaPERK31* had a minimum of three introns (Figure S5). The size of an intron is a critical player that affects the gene size; for example, a notable difference in gene size was found between the biggest gene *TaPERK17* (4 kb) and the smallest gene *TaPERK23* (2.1 kb), and this was mainly caused by the total intron length (4 kb vs. 1.1 kb). Many studies have shown the significance of introns in the evolution of numerous plant genes [63,64]. Several gene families had less, lack, or more introns in their gene families [7,59,65,66]. The exon and intron differences might be due to deletion/insertion events, which would predict the evolutionary processes [67]. All PERK gene family members in cotton had no introns, indicating that GhPERK genes might have evolved comparatively quickly [7].

Furthermore, it has been established that gene families containing larger or more introns can acquire new functions during evolution processes. There were more intron gains than losses in the plant lineages and chordates, while in arthropods and fungi, losses prevailed over gains [63,64,67]. In our study, almost all TaPERK genes had more and larger introns. Hence, we can speculate that PERK genes gained new functions during evolution in wheat. Furthermore, conserved motif analysis showed ten different types of motif compositions amidst the TaPERK proteins. We observed that five motifs were found in all the TaTERK proteins (Motif 1, 2, 3, 4 and 6), and proteins of the same subfamilies usually shared the same motifs and were more conservative. Thus, we hypothesize that proteins of the same subfamilies may have the same function.

Additionally, amino acid sequence alignment of TaPERK with other plant species PERK proteins also showed that all TaPERK proteins consisted of a conserved tyrosine kinase domain (Figure 6 and Figure S6). The amino acid residues of PERK were highly conserved in rice, Arabidopsis, soybean, and wheat, which might be helpful to find the pattern of PERK protein sequence conservation in different plant species. Yang and colleagues also found that YABBY and WOX gene families were evolutionarily conserved in cotton [57,58]. Furthermore, 3D protein structure analysis revealed that TaPERKs had two distinct subdomains, a smaller N-terminal lobe, and a more prominent C-terminal lobe connected by a small hinge loop (Figure S6A,B). These findings will be helpful to understand and explain the substrate specificity and molecular function of TaPERK genes in activating the PERK signal transduction pathway.

The *cis*-acting regulatory element in the promoter plays an important role in regulating and functioning genes [68]. The promoter region of TaPERKs gene families contains the multiple cis-acting elements related to plant hormones, growth, development, defense, and stress-related functions (Figure 7A and Table S6). We predicted more than eight CAREs in the promoter region of each TaPERK (Table S6). A total of 15 CAREs related to light response were detected, including AE-box, Box 4 and ATCT motif, chs-Unit 1 m1, TCT-motif, I-box, chs-CMA1a, chs-CMA2a, GA-motif, GATA-motif, LAMP-element and TCCC-motif, ACE and GT1-motif, Sp1 and 3-AF1 binding site [69,70]. We also detected the six CAREs related to growth and development, such as MSA-like (cell cycle regulation), GCN4-motif (endosperm expression), O2-site (zein metabolism regulation), CAT-box (meristem expression), RY-element (seed-specific regulation) and CAAAGATATC-motif (circadian control) [71,72]. In addition, we also found the CARE related to phytohormone response, for instance, CGTCA-motif (MeJA-responsive element), ABRE (abscisic acid-responsive element), TCA-element (salicylic acid responsiveness), TGA-motif (auxin-responsive element), P-box, and GARE-motif (gibberellin-responsive element). The MeJA-responsive element was predicted in most TaPERK genes except *TaPERK8*, *TaPERK11*, *TaPERK23*, and *TaPERK28*. Moreover, we also predicted that other *cis*-elements had been involved in different stress conditions, such as LTR (low-temperature responsiveness), MBS (drought inducibility), and TC-rich repeats (defense and stress responsiveness) in the TaPERK promoters. Several studies have demonstrated that light plays a crucial role in plant growth and development processes [73]. Several CAREs related to low temperature, fungal elicitors, stress and defense, auxins, MeJA, gibberellin, ethylene abscisic acid, and the salicylic acid-responsive element were also predicted in *GhPERK* and *BrPERK* gene promoter regions [7,15]. In this study, almost all TaPERK genes contained the multiple CAREs involved in plant growth and the responses to diverse stress. *GhPERK8*, *GhPERK 9*, *GhPERK12*, *GhPERK23*, *GhPERK27*, and *GhPERK29* expression levels were elevated upon exposure to plant hormones such as indole-3-acetic acid, gibberellin, salicylic acid, and MeJA; however, the expression level of *GhPERK5* declined [7]. *PERK4* regulates the root growth function at an early stage of ABA signaling by perturbing calcium homeostasis in Arabidopsis [14]. *PERK1* rapidly induced early perception and response to a wound stimulus in Chinese cabbage [12]. Antisense suppression of *BnPERK1* exhibited various growth defects such as amplified secondary branching, loss of apical dominance, and defects in floral organ formation. At the same time, the overexpression line showed increased lateral shoot production, seed set, and unusual deposition of callose and cellulose in *Brassica napus* [21]. Collectively, these results showed that PERKS gene family members might regulate diverse biological processes, responses to phytohormones, and work against different biotic and abiotic stress. Of course, this needs to be established by experimental studies in the near future. Therefore, these data provide the valuable information to understand TaPERKs’ function in plant growth and development, response to phytohormones, and different stresses.

Receptor kinases play a critical role in different biological processes and responses to internal and external stimuli [8,9,12,13,14,15,16,21,25]. Different TaPERK genes displayed differential expressions in various tissues. For example, *TaPERK2*, *TaPERK4*, *TaPERK13*, *TaPERK21*, *TaPERK23*, and *TaPERK25* exhibited induction at the spike z39 stage, while *TaPERK15*, *TaPERK20*, *TaPERK27*, *TaPERK29*, and *TaPERK36* exhibited induction at spike z65 stage (Figure 8). The expression of *TaPERK5*, *TaPERK6*, *TaPERK7*, *TaPERK8*, *TaPERK9*, *TaPERK11*, *TaPERK12*, *TaPERK17*, *TaPERK22*, *TaPERK26*, *TaPERK28*, *TaPERK32*, *TaPERK33*, *TaPERK34*, *TaPERK35*, and *TaPERK37* were elevated in the roots at the z13 and z39 stages, respectively. *TaPERK10*, *TaPERK30*, and *TaPERK31* were also up-regulated in the leaf at the z23 and z71 stages, respectively. In addition, *TaPERK13*, *TaPERK14*, *TaPERK23*, *TaPERK24*, and *TaPERK31* showed induction at the grain z71 stage. *TaPERK18* and *TaPERK24* showed higher expression at the stem z30 stage, whereas *TaPERK24*, *TaPERK30*, and *TaPERK31* expressions were raised at the stem z65 stage. PERKs proteins have been involved in various developmental processes, including cell differentiation, pollen tube growth, pollen development, symbiosis, pathogen recognition, phytohormone response, signal transduction, and self-incompatibility [9,12,14,16,17,21]. AtPERK gene family members are ubiquitously expressed, while few genes are specifically expressed [6]. For instance, *AtPERK1* is broadly expressed, whereas *AtPERK2* is mainly expressed in rosette leaf veins, stems, and pollen [21,32]. *AtPERK8* and *AtPERK13* expression were found in the root hairs [6,22,23]. In addition, *AtPERK5*, *AtPERK6*, *AtPERK7*, *AtPERK11*, and *AtPERK12* expression were up-regulated in the pollens [23,24,25]. Furthermore, PERK4 modulates the root tip growth at an early stage of ABA signaling via the disruption of calcium homeostasis in Arabidopsis [14]. Some researchers have shown that increased calcium concentration in the cells also enhances antioxidant enzyme activities to regulate the lipid peroxidation of cell membranes and stomatal aperture [26,27,28]. *AtPERK5* and *AtPERK12* play an essential role in pollen tube growth in Arabidopsis [16]. Furthermore, *AtPERK8*, *AtPERK9*, and *AtPERK10* negatively regulate root growth in Arabidopsis [23]. The expression level of twelve GhPERK genes was significantly elevated in leaves and ovule development in cotton [7]. BrPERK genes were differentially expressed in various tissues of Chinese cabbage, but some BrPERK genes were specially expressed in reproductive organs [15]. Our GO analysis also indicated the critical roles of the TaPERK gene in the cell (Figure S7A–C). Thus, this spatial and temporal expression of the TaPERK genes suggests that these PERKs might have an essential function in different wheat tissue.

Receptor kinases are crucial in plant adaptations and responses to internal and external stimuli [8,9,12,13,14,15,74]. Our results also showed that several TaPERK gene family members’ expressions were elevated in different stress conditions (Figure S9). *TaPERK9*, *TaPERK13*, *TaPERK15*, *TaPERK17*, *TaPERK20*, *TaPERK22*, *TaPERK23*, *TaPERK26*, *TaPERK28*, *TaPERK33*, *TaPERK35,* and *TaPERK36* were induced during the septoria tritici blotch, while the expressions of *TaPERK2*, *TaPERK6*, *TaPERK7*, *TaPERK8*, *TaPERK10*, *TaPERK11*, *TaPERK12 TaPERK27*, *TaPERK35,* and *TaPERK37* were significantly raised after powdery mildew infection. *TaPERK1*, *TaPERK8*, *TaPERK16*, *TaPERK21*, *TaPERK24*, *TaPERK25*, *TaPERK29*, *TaPERK30*, *TaPERK31,* and *TaPERK34* were up-regulated during the stripe rust infection. Furthermore, *TaPERK3*, *TaPERK4*, *TaPERK18,* and *TaPERK32* were induced during the initial hours of heat stress. However, none of the genes were expressed in drought stress. It seems that the TaPERK family does not participate in drought stress, and only *TaPERK4* and *TaPERK18* genes were elevated during the combined drought and heat stress (Figure S9). Furthermore, *TaPERK14*, *TaPERK19*, *TaPERK24*, and *TaPERK29* expression levels were significantly elevated in cold stress. Most TaPERKs respond similarly to biotic and abiotic stress; hence, all stress-responsive genes cluster together (Figure S8B). Several PERKS genes in *A. thaliana, G. hirsutum,* and *B. rapa* were responsive to diverse abiotic stresses, including cold, salt, heat, and PEG, indicating that PERK genes play a critical role in other plant species to adapt to different stress conditions [6,7,15,23]. PERK1 rapidly induced early perception and response to a wound stimulus in Chinese cabbage [12]. A PERK-like receptor kinase specifically interacts with the nuclear shuttle protein (NSP), led viral infection, and positively regulates the NSP function in cabbage leaf curl virus and geminivirus [32]. The expression profile of TaPERK genes under different stresses indicated that they might participate in the diverse biotic and abiotic stress tolerance in wheat. Therefore, these findings demonstrated that TaPERK genes respond to various stresses, and this might be used for breeding wheat lines to develop stress-tolerant varieties in wheat.

Many studies have shown that receptor kinases at the cell surface perceive a sudden changing environment that activates the various signaling pathways, regulating growth, reproduction, and response to diverse stress conditions [2,5,75]. Our protein–protein network analysis revealed that eighteen TaPERKs interacted with 10 different wheat proteins (Figure 10 and Table S9). TaPERK29 can interact with seven other wheat proteins (Traes_3AL_394642923.1, Traes_3AL_5E8DEE3E8.1, Traes_3B_514AAB5F3.1, Traes_3B_9EBD47B52.1, Traes_4AS_E28B34320.1, Traes_6BL_DFDCD5B11.1 and Traes_4DL_05BF7F181.1), which were cGMP-dependent protein kinase/PKG II, protein of unknown function (DUF1645) and BRASSINOSTEROID INSENSITIVE 1, playing a critical role in Brassinosteroid signaling. Brassinosteroids play an essential role in various cellular processes, such as cell division, seed germination, vascular differentiation, flowering, xylem cell differentiation, stomata formation, photomorphogenesis, and pollen tube growth [76,77,78,79]. In addition, TaPERK2 and TaPERK4 can interact with four other wheat proteins (Traes_7AL_5E0DD589E.1, Traes_4DL_E447FD9FD.1, Traes_6BL_DFDCD5B11.1 and Traes_7DL_909EA97B3.1) which were cGMP-dependent protein kinase/PKG II and non-specific serine/threonine-protein kinase/Threonine-specific protein kinase. cGMP-dependent protein kinase is a phosphorylated diverse biologically important pathway [45,46,47]. PKG activated by cGMP has been implicated in cell division regulation, nucleic acid synthesis response to biotic stress, stomata closure during osmotic stress, and the development of adventitious roots [45,46,47,48,49]. TaPERK29 was highly elevated during the stripe rust infection, and cold stress might interact with Traes_6BL_DFDCD5B11.1, that is, cGMP-dependent protein kinase/PKG II specifically activates the signal transduction pathways implicated in different stress tolerance in wheat.

Moreover, TaPERK2 was co-expressed with TaPERK4 in spike development (Figure 8 and Figure S9), indicating that TaPERK2 and TaPERK4 might have an essential function in spike development through interacting with each other. These results provided valuable insight and the complex biological functions of TaPERK genes. In summary, this study provides valuable information about the TaPERK gene family, functions in plant growth, and response to phytohormones and different stress. Therefore, the outcome of this work is significant to dissect and understand the precise functions of TaPERK in developmental processes and various biotic and abiotic stress in wheat.

## 4. Materials and Methods

### 4.1. Identification of PERK Genes in Wheat

To carry out the genome-wide survey in bread wheat (*Triticum aestivum*) cv. *Chinese Spring*, genome data including genomic, CDS, and protein sequences of TaPERK genes were downloaded from the Ensembl plants biomart (http://plants.ensembl.org/biomart/martview, accessed on 27 September 2021). Two approaches were used to identify the PERK genes family in wheat. In the first method, we prepared a local database of the wheat protein sequences in BioEdit ver. 7.2.6 [80]. The sixty-two PERK genes from *A. thaliana*, *G. max*, *O. sativa*, and *Z. mays* were used for BLASTp against the local database. To find PERK genes, the e-value of 10^−5^ and >100-bit score were kept cut-off, and eventually, the BLASTp result was tabulated. In the second approach, the protein sequences of PERK from the above plant species were retrieved from the Ensembl Plants (http://plants.ensembl.org/index.html, accessed on 27 September 2021) and the BLASTp search was performed against the *T. aestivum* proteome with an e-value 10^−5^ and bit-score > 100. Based on the above method, putative PERK candidates were selected. Further putative PERK candidates were confirmed for the presence of protein tyrosine kinase domain using other online databases: InterPro (https://www.ebi.ac.uk/interpro, accessed on 25 September 2021), Simple Modular 132 Architecture Research Tool tool (SMART, http://smart.emblheidelberg.de/, accessed on 25 September 2021), HMMscan (https://www.ebi.ac.uk/Tools/hmmer/search/hmmscan, accessed on 25 September 2021) and NCBI CDD (https://www.ncbi.nlm.nih.gov/Structure/cdd/cdd.shtml, accessed on 25 September 2021). Finally, the protein sequences with protein tyrosine kinase domains were taken and renamed according to their chromosomal positions.

### 4.2. Genomic Localization, Gene Duplication, and Synteny Analysis

To map the chromosomal locations of TaPERK genes, genomic positions of PERK genes were downloaded from Ensembl plants biomart (http://plants.ensembl.org/biomart/martview, accessed on 26 September 2021). The PERK genes were named with a ‘Ta’ prefix and numbered according to their chromosomal positions. PhenoGram was used to map the TaPERK genes on the chromosomes (http://visualization.ritchielab.org/phenograms/plot, accessed on 26 September 2021). MCScanX tool kit was used to examine gene duplication events and synteny analysis within species and other plant species [81,82]. We used default parameters in MCScanx for synteny analysis. The non-synonymous (Ka) and synonymous substitution (Ks) ratio was calculated to estimate the selection pressure of duplicated TaPERK genes using the TBtools [82].

### 4.3. Biophysical Characteristics, Subcellular Localization, and 3D Structure

The biophysical characteristics of TaPERK proteins were predicted using ExPASy [83] and an isoelectric point calculator [84]. Subcellular localization was evaluated using CELLO [85], softberry (www.softberry.com, accessed on 27 September 2021), and BUSCA [86]. Finally, the three-dimensional structure of TaPERKs was generated using the Phyre2 web server [87].

### 4.4. Exon/intron Structure, Protein Motif, and Gene Ontology Analysis

The coding sequence, genomic and protein sequences of TaPERK genes were downloaded from the Ensembl plants biomart (http://plants.ensembl.org/biomart/martview, accessed on 27 September 2021). Exon, intron positions, and untranslated regions were elucidated using the Gene Structure Display Server 2.0 (http://gsds.gao-lab.org/, accessed on 27 September 2021). The protein motifs in the TaPERK were visualized using MEME (Multiple Em for Motif Elicitation ver.5.3.3; http://meme-suite.org/tools/meme, accessed on 27 September 2021) with default settings. TaPERK protein sequences were explored to detect GO terms enrichment using EggNOG (http://eggnogdb.embl.de/#/app/emapper, accessed on 27 September 2021) and agriGO [88].

### 4.5. Promoter Cis-Acting Regulatory Elements (CAREs) and Protein Interaction Network Analysis

To identify *cis*-elements, 2 kb upstream sequences of PERK genes were retrieved from Ensembl plants and examined using a PlantCARE online webserver (http://bioinformatics.psb.ugent.be/webtools/plantcare/html/, accessed on 28 September 2021). The number of occurrences for each *cis*-element motif was counted for TaPERK genes, and the most frequently occurring CAREs were used to generate Figure 7 using TBtools [82]. The TaPERK protein interaction network was predicted using the STRING webserver (https://string-db.org/cgi, accessed on 28 September 2021).

### 4.6. Expression Analysis of TaPERK Genes

Transcripts per million (TPM) values for five different tissues, including leaf, stem, root, spike, grain, and under various stress conditions, were downloaded from the Wheat Expression database (http://www.wheat-expression.com/, accessed on 30 September 2021). Heatmaps and principal component analysis (PCA) were performed using ClustVis [89] and TBtools software [82].

### 4.7. Plant Growth Conditions, Stress Treatment, and RT-qPCR Analysis

Wheat (*Triticum aestivum* L.) cv. HI 1612 was used for the experiments. Seeds of HI 1612 were sown on soil in plastic pots and reared in a greenhouse. Ten-day-old wheat seedlings were acclimatized for two days in growth chamber conditions. They were further subjected to drought and high-temperature stress (40 °C) for 1 h and 6 h [90], and cold stress for 3 days (4 °C). For the combined drought and high-temperature stress, first, wheat seedling was exposed for the drought stress, then given a heat shock for 1 h and 6 h at 40 °C in an incubator. Controls were kept at 25 °C. The cold, drought, and high-temperature stressed seedlings were collected for RNA extraction and stored at −80 °C. The RNA was isolated from control, drought, and heat-stressed seedlings, as described by [91,92]. cDNA was synthesized using the iScriptTM cDNA synthesis kit (Bio-Rad, Hercules, CA, USA). Quantitative real-time PCR (RT-qPCR) was performed using the Applied Biosystems 7500 Fast Real-Time PCR (Applied Biosystems) with the SYBR Premix (Toyobo, Osaka, Japan). Wheat actin (AB181991) was used as a control to normalize the gene expression data. Transcript abundance was analyzed using the RT-qPCR. Each qRT-PCR reaction was carried out with three biological samples with two technical replicates and repeated three times. The fold change was calculated based on mean 2^−ΔΔCT^ values and, eventually, this fold value was used to plot the graph [93,94]. Furthermore, one-way ANOVA, followed by Tukey’s HSD for multiple pairwise comparisons were applied. Means, standard errors and statistical significances for each sample were represented in figures (* *p* < 0.05, ** *p* < 0.01). All primers used in this study are mentioned in Table S10.

## 5. Conclusions

Wheat is the most important cereal crop and widely consumed staple food worldwide. However, global warming is becoming a severe threat to food security due to the constant climate changes, largely influencing plant development and productivity. This has raised a major challenge for plant biologists to increase yield and improve wheat’s quality, biotic and abiotic stress tolerance. The PERKS gene family plays a critical role in plant development and responses to various stresses. We identified and characterized the PERK gene family in wheat in this work. Expression patterns also revealed the role of TaPERKs in different developmental stages and stress conditions. Thus, this study facilitates a detailed understanding of PERK genes’ biological functions in wheat under different developmental processes and stress conditions.

## Figures and Tables

**Table 1:** Nomenclature and characteristics of the putative proline-rich extensin-like receptor kinases (PERKs) proteins in wheat were predicted using various computational tools.

Proposed Gene Name	Gene ID	Genomic Location	Orientation	CDS Length (bp)	Intron Number	Protein Length (aa)	Molecular Weight (KDa)	Isoelectric Point (pI)	GRAVY	Predicted Subcellular Localization
*TaPERK1*	TraesCS1A02G127900	1A:155693812–155696618	Forward	1977	7	658	69.44	7.53	−0.531	Nucleus
*TaPERK2*	TraesCS1B02G1470000	1B:209130189–209130266	Reverse	1431	8	476	52.14	6.17	−0.5	Nucleus
*TaPERK3*	TraesCS1D02G00430	1D:2110107–2112027	Forward	1971	7	656	68.93	9.04	−0.393	Chloroplast outer membrane
*TaPERK4*	TraesCS1D02G126300	1D:137437684–137440387	Reverse	1962	7	653	69.07	7.21	−0.52	Nucleus
*TaPERK5*	TraesCS2A02G418200	2A:674030843–674031911	Forward	3048	23	1015	110.33	6.33	−0.193	Plasma membrane
*TaPERK6*	TraesCS2A02G418300	2A:674050369–674051442	Forward	3042	23	1013	110.39	7.06	−0.135	Plasma membrane
*TaPERK7*	TraesCS2A02G418400	2A:674061248–674062244	Forward	3159	23	1052	113.73	5.96	−0.127	Plasma membrane
*TaPERK8*	TraesCS2B02G437200	2B:629023953–629025021	Forward	3045	23	1014	110.38	6.3	−0.178	Plasma membrane
*TaPERK9*	TraesCS2B02G437300	2B:629106216–629107285	Forward	3048	23	1015	110.62	6.61	−0.147	Plasma membrane
*TaPERK10*	TraesCS2D02G415600	2D:529537635–529538701	Forward	3048	22	1015	110.34	6.6	−0.167	Plasma membrane
*TaPERK11*	TraesCS2D02G415700	2D:529548057–529548998	Forward	2775	23	924	101.07	7.29	−0.182	Plasma membrane
*TaPERK12*	TraesCS2D02G415800	2D:529558487–529559547	Forward	3156	23	1051	113.45	6.11	−0.102	Plasma membrane
*TaPERK13*	TraesCS3A02G003900	3A:1925607–1927275	Reverse	2064	7	687	72.42	5.96	−0.429	Plasma membrane
*TaPERK14*	TraesCS3A02G152200	3A:142891955–142894634	Forward	1893	7	630	67.43	6.28	−0.569	Endomembrane system
*TaPERK15*	TraesCS3A02G229800	3A:429615911–429617422	Reverse	2163	6	720	74.97	7.93	−0.401	Chloroplast thylakoid lumen
*TaPERK16*	TraesCS3A02G278100	3A:507637093–507638935	Reverse	2028	7	675	72.42	7.31	−0.481	Plasma membrane
*TaPERK17*	TraesCS3A02G290300	3A:519244808–519246110	Reverse	2184	7	727	75.8	6.11	−0.535	Endomembrane system
*TaPERK18*	TraesCS3B02G008600	3B:4324660–4326408	Forward	2061	7	686	71.88	5.97	−0.437	Plasma membrane
*TaPERK19*	TraesCS3B02G179300	3B:187347873–187350697	Forward	1896	7	631	67.46	6.35	−0.569	Endomembrane system
*TaPERK20*	TraesCS3B02G259100	3B:416806224–416809608	Reverse	2097	6	698	72.98	7.63	−0.448	Plasma membrane
*TaPERK21*	TraesCS3B02G312300	3B:501498044–501499926	Reverse	2034	7	677	72.6	7.31	−0.493	Endomembrane system
*TaPERK22*	TraesCS3B02G325100	3B:525990462–525991846	Reverse	2436	7	811	84.77	6.09	−0.51	Plasma membrane
*TaPERK23*	TraesCS3D02G005400	3D:2141185–2143272	Forward	1206	6	401	44.37	5.55	−0.403	Nucleus
*TaPERK24*	TraesCS3D02G160000	3D:130928685–130931461	Forward	1899	7	632	67.49	6.36	−0.555	Endomembrane system
*TaPERK25*	TraesCS3D02G278400	3D:385473929–385474240	Reverse	2031	8	676	72.56	7.1	−0.471	Endomembrane system
*TaPERK26*	TraesCS3D02G290100	3D:400311470–400312883	Reverse	1317	6	438	47.14	5.97	−0.447	Nucleus
*TaPERK27*	TraesCS4A02G077500	4A:76627667–76628358	Forward	1866	5	621	64.49	5.58	−0.457	Endomembrane system
*TaPERK28*	TraesCS4A02G449700	4A:715718345–715719349	Forward	2604	19	867	94.75	7.03	−0.145	Plasma membrane
*TaPERK29*	TraesCS4B02G233600	4B:486206279–486206961	Reverse	1857	5	618	64.45	5.63	−0.487	Plasma membrane
*TaPERK30*	TraesCS5A02G411300	5A:599978835–599979642	Reverse	1722	4	573	60.33	7.96	−0.387	Chloroplast outer membrane
*TaPERK31*	TraesCS5B02G415000	5B:589228532–589228944	Reverse	1842	3	613	64.68	7.86	−0.368	Chloroplast outer membrane
*TaPERK32*	TraesCS7A02G038600	7A:17358644–17359648	Reverse	3030	23	1009	109.7	6.22	−0.108	Plasma membrane
*TaPERK33*	TraesCS7A02G231900	7A:202852283–202853761	Reverse	2187	6	728	76.24	5.32	−0.484	Nucleus
*TaPERK34*	TraesCS7B02G130400	7B:156752944–156754400	Reverse	2208	6	735	76.89	5.22	−0.49	Nucleus
*TaPERK35*	TraesCS7D02G034800	7D:17864178–17865182	Reverse	3021	23	1006	109.26	6.1	−0.09	Plasma membrane
*TaPERK36*	TraesCS7D02G232700	7D:194224547–194225929	Forward	2256	6	751	78.2	6.16	−0.645	Endomembrane system
*TaPERK37*	TraesCSU02G104700	Un:92294980–92296477	Reverse	2205	6	734	76.55	5.32	−0.483	Nucleus

ID: identity; bp: base pair; aa: amino acids; pI: isoelectric point; MW: molecular weight; KDa: Kilo dalton.

**Table 2:** Number of PERK proteins in different plant species.

Plant Species	Genome Size (Approx.)	Coding Genes	PERK Genes
*Triticum aestivum* (6n)	17 Gb	107,891	37
*Arabidopsis thaliana* (2n)	135 Mb	27,655	15
*Oryza sativa*	500 Mb	37,960	8
*Zea mays* (2n)	2.4 Gb	39,591	23
*Glycine max* (2n)	1.15 Gb	55,897	16
*Sorghum bicolor* (2n)	730 Mb	28,120	15
*Gossypium arboretum* (2n)	1746 Mb	41,330	15
*Gossypium raimondii* (2n)	885 Mb	40,976	16
*Gossypium hirsutum* (4n)	2.43 Gb	75,376	33

## Data Availability

Data is available in the manuscript and in the Supplementary Materials.

## Supplementary Materials

The following are available online at https://www.mdpi.com/article/10.3390/plants11040496/s1. Figure S1: Molecular weight (kDa) vs. isoelectric point plots of TaPERK genes. The distinct round shape colors represent the TaPERK gene family members. Figure S2: Distribution of TaPERKs in a different group of the phylogenetic tree. The *y*-axis indicates the number of TaPERK genes, and the *x*-axis indicates the phylogenetic groups. Figure S3: Phylogenetic analysis of TaPERK genes. A phylogenetic tree was constructed using MEGAX with the neighbor-joining (NJ) method and 1000 bootstrap replications. A black asterisk indicates the duplicated genes. Figure S4: Chromosomal distribution and duplicated PERK gene pairs in wheat. Duplicated PERK gene pairs are connected with lines with distinct colors. The figure was generated using TB tools. Figure S5: Distribution of exon and introns in TaPERKs gene family. The *y*-axis indicates the number of exons and introns, and the *x*-axis indicates the TaPERK genes. Exons and introns are represented by purple and orange boxes, respectively. Figure S6: Alignment and 3-dimensional structure of the TaPERK protein sequences. A. The conserved protein tyrosine kinase domain is boxed with red color. Colored and shaded amino acids are chemically similar residues. Dashes indicate gaps introduced to maximize the alignment of the homologous region. B. Predicted 3D structures TaPERK proteins. Figure S7: Gene ontology term distribution TaPERK gene family predicted using AgriGO A. Biological Process. B. Cellular component. C. Molecular function. Figure S8: PCA plots displaying grouping of different (A) developmental stages (B) biotic and abiotic stress conditions based on the TaPERK expression pattern. DS: drought stress, HS: heat stress, Zt: *Zymoseptoria tritici*, PM: powdery mildew; SR: stripe rust, h: hour and d: days. Figure S9: Heatmaps representing the expression pattern of TaPERK genes in different stress conditions. TPM values were directly used to construct the heatmaps. DS: drought stress, HS: heat stress, Zt: *Zymoseptoria tritici*, PM: powdery mildew; SR: stripe rust, h: hour and d: days, Table S1: TaPERK genomic, CDS protein and promoter sequence. Table S2: PERK proteins from Arabidopsis, rice, soybean, and wheat used to generate a phylogenetic tree. Table S3: Ratio of Ka/Ks and distribution of duplicate wheat PERK genes. Table S4: Orthologous relationships of TaPERK genes with other PERK genes in *B. distachyon*, *Ae. tauschii*, *T. dicoccoides*, *O. sativa* and *A. thaliana*. Table S5: Domain organization of *TaPERK* genes predicted using Pfam with default parameters. Table S6: *cis*-regulatory elements present in the *TaPERK* gene promoter region. Table S7: Significant Go term predicted in TaPERK gene family by AgriGo analysis. Table S8: *TaPERK* gene annotation using eggNOGmapper. Table S9: The protein-protein interaction network between TaPERK and other proteins in wheat. Table S10: qRT-PCR primers of TaPERK genes.
