# Supplementary material for: Genome-Wide Analysis and Characterization of the Proline-Rich Extensin-like Receptor Kinases (PERKs) Gene Family Reveals Their Role in Different Developmental Stages and Stress Conditions in Wheat (Triticum aestivum L.)
Source: Plants (Basel). 2022 Feb 11;11(4):496. doi: 10.3390/plants11040496 (PMC8880425; doi:10.3390/plants11040496)
Supplement: Supplementary file 1 [file plants-11-00496-s001.zip › sup/TaPERK supplmentary figures.pdf]

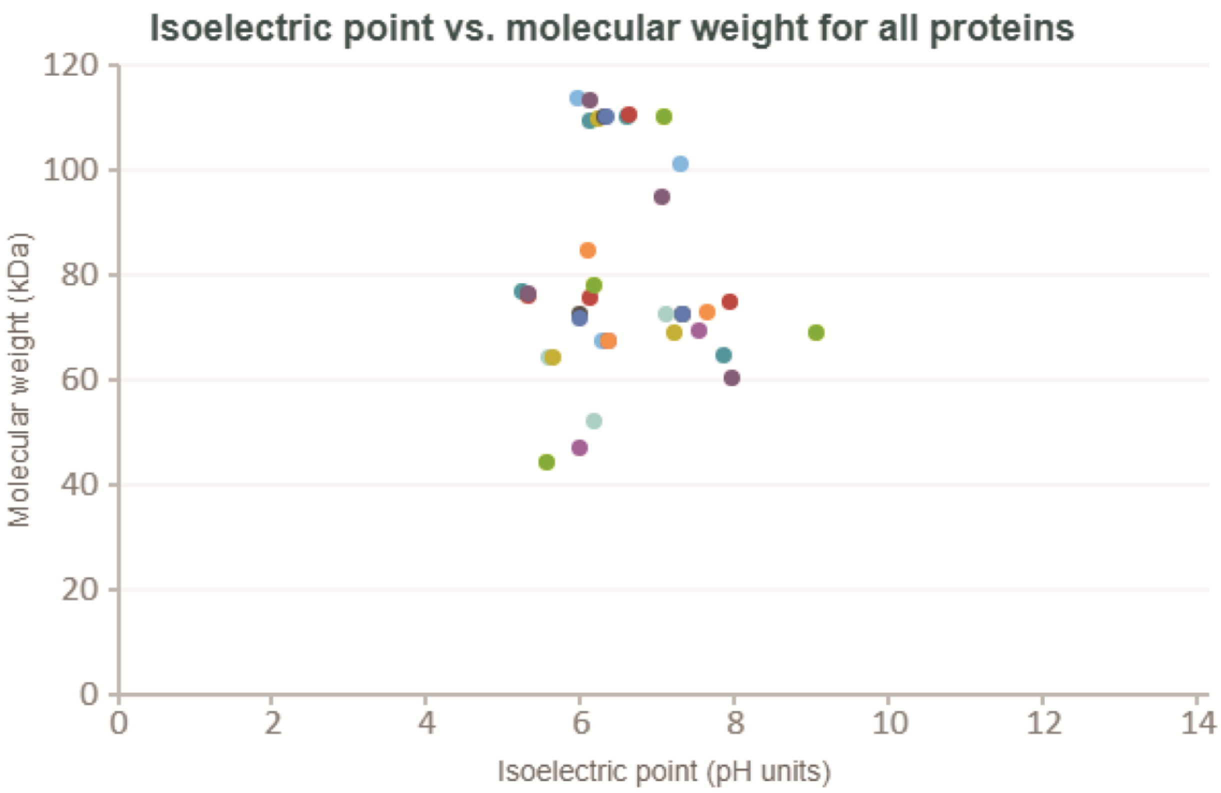

Figure S1. Molecular weight (kDa) vs. isoelectric point plots of TaPERK genes.

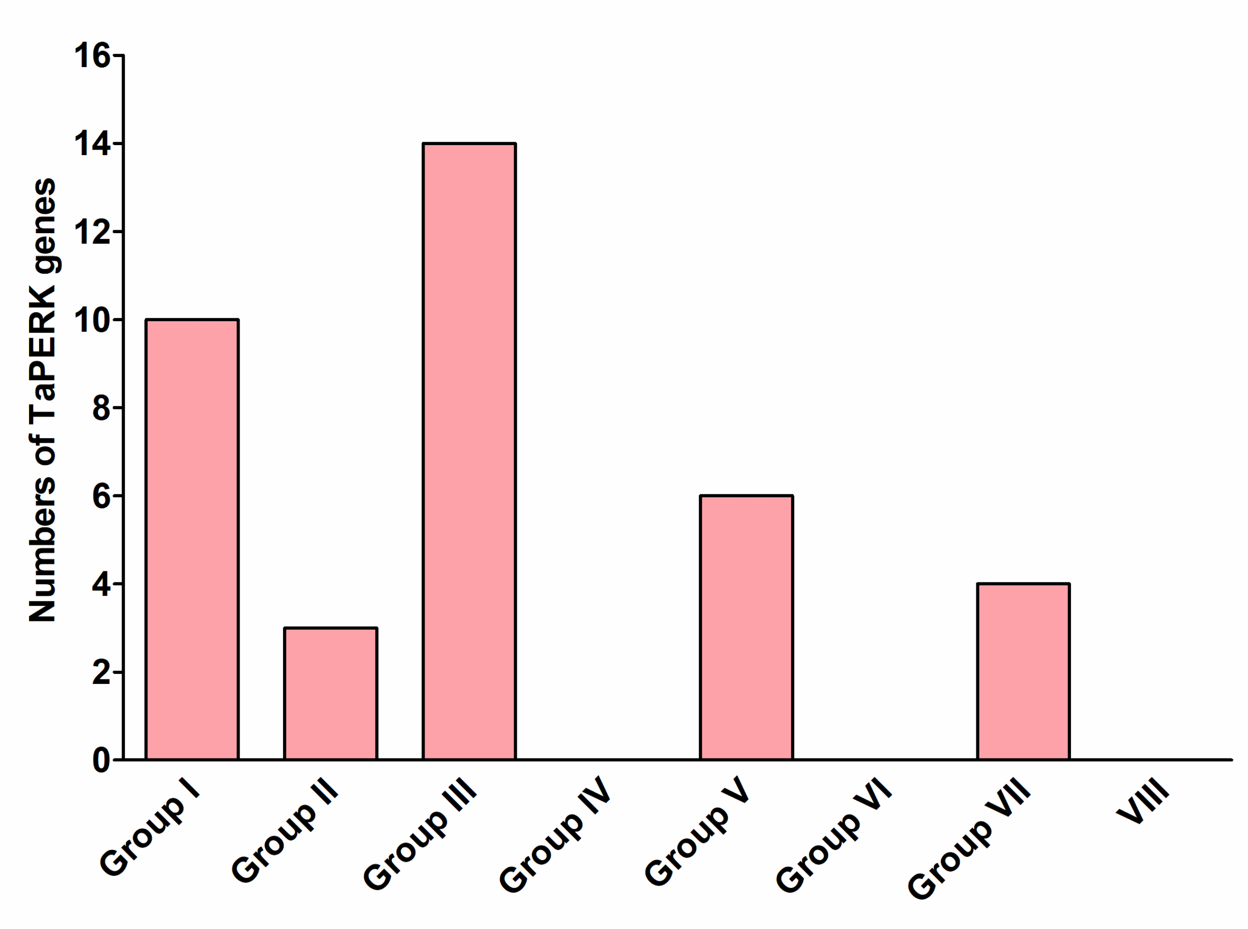

Figure S2. Distribution of TaPERKs in a different group of the phylogenetic tree.

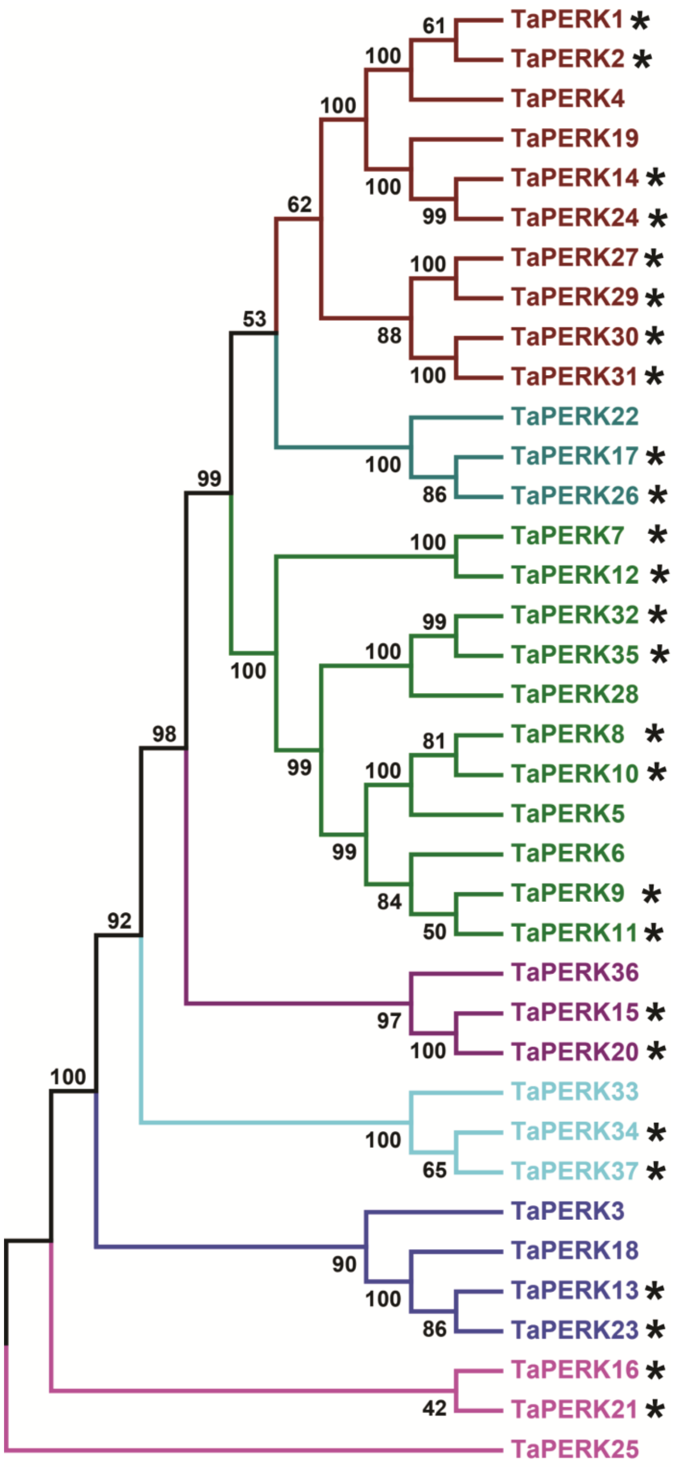

Figure S3. Phylogenetic analysis of TaPERK genes. A phylogenetic tree was constructed using MEGAX with the neighbor-joining (NJ) method and 1000 bootstrap replications. A black asterisk indicates the duplicated genes.

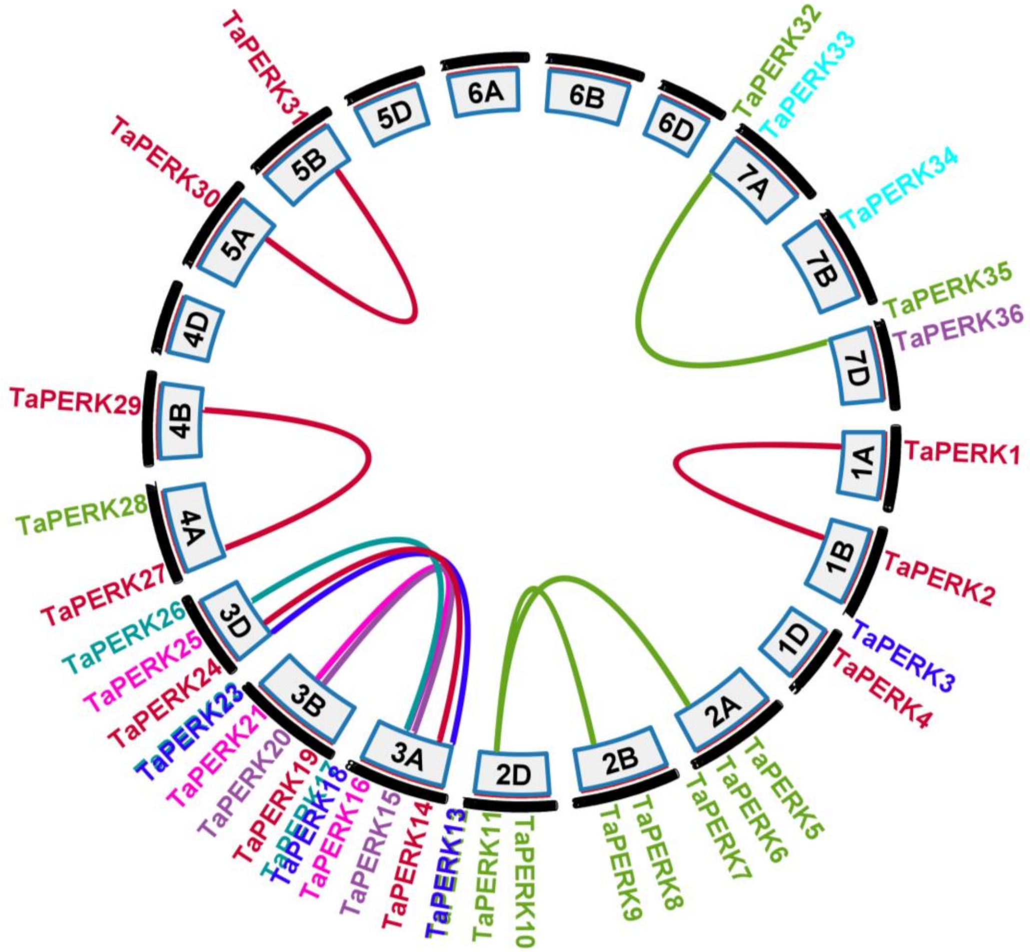

Figure S4. Chromosomal distribution and duplicated PERK gene pairs in wheat. Duplicated PERK gene pairs are connected with lines with distinct colors. The figure was generated using TB tools.

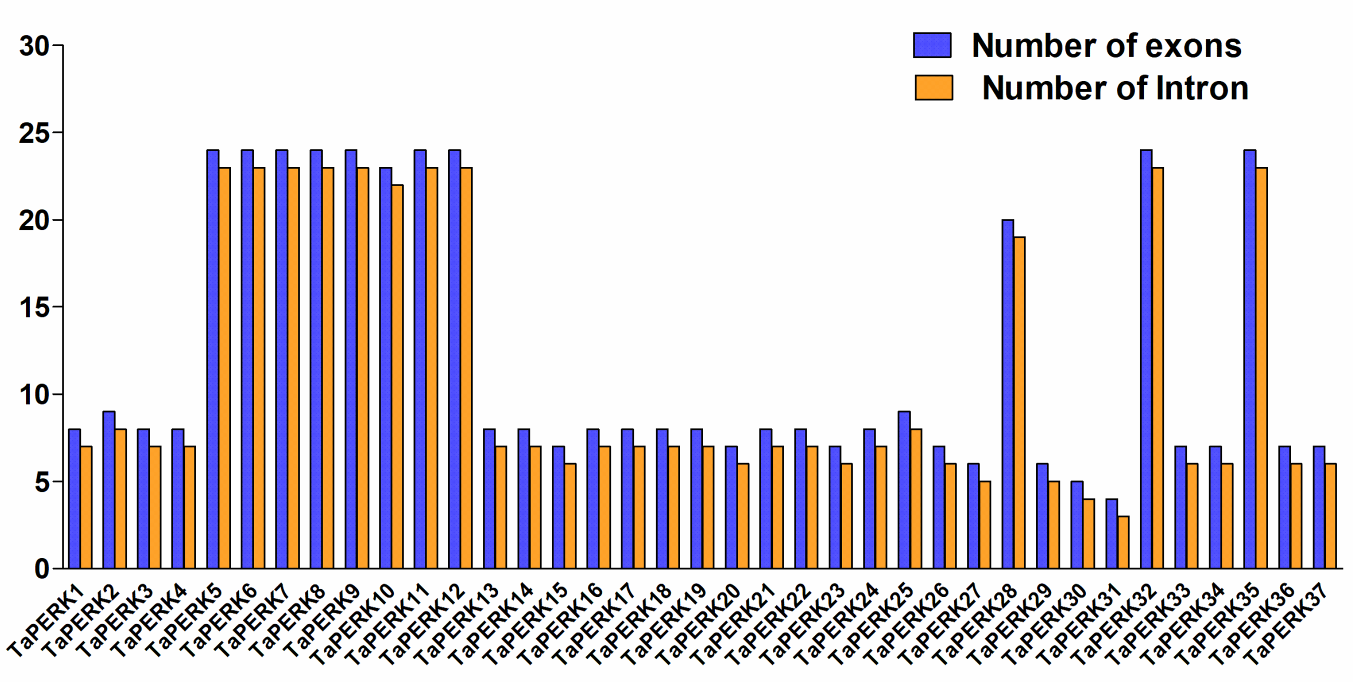

Figure S5. Distribution of exon and introns in TaPERKs gene family.

A

```
                   *        20         *        40         *        60         *        80
TaPERK1   : ------------------------------------------------------------------------------------- :  -
TaPERK2   : ------------------------------------------------------------------------------------- :  -
TaPERK3   : ------------------------------------------------------------------------------------- :  -
TaPERK4   : ------------------------------------------------------------------------------------- :  -
TaPERK5   : -----MGRRRQALHGAALSL--LLLAAA------AHAQQTDP-------GDAAALNAVFAKLGQKALSSWNISGDPCTGAATDNT : 65
TaPERK6   : ---------MKLLHGAALAL--LLLAAA------AHAQRTPRTH----AGDAAALNAVFAKLGQKASSSWNISGNLCTGAAIDDT : 64
TaPERK7   : MGPPSPRWSSCLRHGSVLLLPLLLTALSSSWRAAAQAQAPPPPPSQTDPTEAAAVNAILGKLGLSAPASWNISGNPCSGAATDDT : 85
TaPERK8   : -----MGRRRQVLHGAALSL--LLLAAV------ADAQQTDP-------GDAAALNAVFAKLGQKALSSWNISGDPCTGAATDNT : 65
TaPERK9   : ---------MKLLLGAALTLPLLLLTAA------AHAQRSPRTH----LGDAAALNAVFAKLGQKASSSWNISGNLCTGAATDDT : 66
TaPERK10  : -----MGRRSRLLHGAALSL--LLLAAA------AHAQQTDP-------GDAAALNAVFAKLGQKALPSWNISGDPCTGAATDNT : 65
TaPERK11  : --------------------------------------------------MSSQLSNVKWSLGQST------------------- : 16
TaPERK12  : MGRPSPGWSSCLRHGPVLLLPLLLSALCSSWRAAAQAQAPPPPP-QTEPTEAAAVNAILGKLGLSAPASWNISGNPCSGAATDDT : 84
TaPERK13  : ------------------------------------------------------------------------------------- :  -
TaPERK14  : ------------------------------------------------------------------------------------- :  -
TaPERK15  : ------------------------------------------------------------------------------------- :  -
TaPERK16  : ------------------------------------------------------------------------------------- :  -
TaPERK17  : ------------------------------------------------------------------------------------- :  -
TaPERK18  : ------------------------------------------------------------------------------------- :  -
TaPERK19  : ------------------------------------------------------------------------------------- :  -
TaPERK20  : ------------------------------------------------------------------------------------- :  -
TaPERK21  : ------------------------------------------------------------------------------------- :  -
TaPERK22  : ------------------------------------------------------------------------------------- :  -
TaPERK23  : ------------------------------------------------------------------------------------- :  -
TaPERK24  : ------------------------------------------------------------------------------------- :  -
TaPERK25  : ------------------------------------------------------------------------------------- :  -
TaPERK26  : ------------------------------------------------------------------------------------- :  -
TaPERK27  : ------------------------------------------------------------------------------------- :  -
TaPERK28  : ------------------------------------------------------------------------------------- :  -
TaPERK29  : ------------------------------------------------------------------------------------- :  -
TaPERK30  : ------------------------------------------------------------------------------------- :  -
TaPERK31  : ------------------------------------------------------------------------------------- :  -
TaPERK32  : ---MREMRLFMILHGAALLL---LLAAV------AHAQQTRTTD----PADAAALHAVFAKLGQKAGPQWNISGDPCTGAAIDNT : 69
TaPERK33  : ------------------------------------------------------------------------------------- :  -
TaPERK34  : ------------------------------------------------------------------------------------- :  -
TaPERK35  : ------MRLFMILHAVALLL---LLAAV------AHAQQTMRTD----PADAAALHAVFAKLGQKAGPQWNISGDPCTGAAIDNT : 66
TaPERK36  : ------------------------------------------------------------------------------------- :  -
TaPERK37  : ------------------------------------------------------------------------------------- :  -
AtPERK1   : ------------------------------------------------------------------------------------- :  -
AtPERK2   : ------------------------------------------------------------------------------------- :  -
AtPERK3   : ------------------------------------------------------------------------------------- :  -
AtPERK4   : ------------------------------------------------------------------------------------- :  -
AtPERK5   : ------------------------------------------------------------------------------------- :  -
AtPERK6   : ------------------------------------------------------------------------------------- :  -
AtPERK7   : ------------------------------------------------------------------------------------- :  -
AtPERK8   : ------------------------------------------------------------------------------------- :  -
AtPERK9   : ------------------------------------------------------------------------------------- :  -
AtPERK10  : ------------------------------------------------------------------------------------- :  -
AtPERK11  : ------------------------------------------------------------------------------------- :  -
AtPERK12  : ------------------------------------------------------------------------------------- :  -
AtPERK13  : ------------------------------------------------------------------------------------- :  -
AtPERK14  : ------------------------------------------------------------------------------------- :  -
AtPERK15  : ------------------------------------------------------------------------------------- :  -
OsPERK1   : ------------------------------------------------------------------------------------- :  -
OsPERK2   : ------------------------------------------------------------------------------------- :  -
OsPERK3   : ------------------------------------------------------------------------------------- :  -
OsPERK4   : ------------------------------------------------------------------------------------- :  -
OsPERK5   : ------------------------------------------------------------------------------------- :  -
OsPERK6   : ------------------------------------------------------------------------------------- :  -
OsPERK7   : ------------------------------------------------------------------------------------- :  -
OsPERK8   : ------------------------------------------------------------------------------------- :  -
GmPERK1   : ------------------------------------------------------------------------------------- :  -
GmPERK2   : ------------------------------------------------------------------------------------- :  -
GmPERK3   : ------------------------------------------------------------------------------------- :  -
GmPERK4   : ------------------------------------------------------------------------------------- :  -
GmPERK5   : ------------------------------------------------------------------------------------- :  -
GmPERK6   : ------------------------------------------------------------------------------------- :  -
GmPERK7   : ------------------------------------------------------------------------------------- :  -
GmPERK8   : ------------------------------------------------------------------------------------- :  -
GmPERK9   : ------------------------------------------------------------------------------------- :  -
GmPERK10  : ------------------------------------------------------------------------------------- :  -
GmPERK11  : ------------------------------------------------------------------------------------- :  -
GmPERK12  : ------------------------------------------------------------------------------------- :  -
GmPERK13  : ------------------------------------------------------------------------------------- :  -
GmPERK14  : ------------------------------------------------------------------------------------- :  -
GmPERK15  : ------------------------------------------------------------------------------------- :  -
GmPERK16  : ------------------------------------------------------------------------------------- :  -
```

```
                   *      100         *       120         *       140         *       160         *
TaPERK1  : ----------------------------------------------------------------------------------MS- :   2
TaPERK2  : ------------------------------------------------------------------------------------- :   -
TaPERK3  : ------------------------------------------------------------------------------MAAKAPA :   7
TaPERK4  : ----------------------------------------------------------------------------------MS- :   2
TaPERK5  : NIDNNPPFNPAIKCECSGG-NASVCRVTRLKIYALDAVGPIPEELRNLTALTNLDLSQNYLTGPLPSFIGELTRMQYMSFGINAL : 149
TaPERK6  : DIDNDPNFNPAIKCLCSTG-NASLCRITRLKIYALDAVGPIPEELWNLTSLTNLNLAQNYLTGPIPSFIGKLAQMQYLSMGINAL : 148
TaPERK7  : PLDDNPNFNPAIKCDCTDQ-NGTLCHVTRLKINSLDAAGPIPEELRNLTHLIKLDFRKNSLTGPLPAFIGELTALKYITVGTNAL : 169
TaPERK8  : NIDNNPPFNPAIKCECTGG-NASVCRVTRLKIYALDAVGPIPEELRNLTALTNLDLSQNYLTGPLPSFIGELTRMQYMSFGINAL : 149
TaPERK9  : DIDNDPNFNPAIKCLCSTG-NASLCRITRLKIYALDAVGPIPEELWNLTSLTNLNLAQNYLTGPIPSFIEKLDQMQYLSLGINAL : 150
TaPERK10 : NIDNNPPFNPAIKCECTGG-NASVCRVTRLKIYALDAVGSIPEELRNLTALTNLDLSQNYLTGPLPSFIGELTRMQYMSFGINAL : 149
TaPERK11 : -------------VFQAPA-ELAVCR----------------------------NLAQNYLTGPIPSFIGNLAQMQYLSLGINAL :  59
TaPERK12 : PLDDNPNFNPAIKCDCTDQ-NGTLCHVTRLKINSLDAAGPIPEELRNLTHLIKLDFRKNSLTGLLPAFIGELTSLKYITVGTNAL : 168
TaPERK13 : ---------------------------------------------------------------------------------MASS :   4
TaPERK14 : ----------------------------------------------------------------------------------MS- :   2
TaPERK15 : --------------------------------------------------------------------MPRILVESVEHLATAPV :  17
TaPERK16 : ------------------------------------------------------------------MGGYADPFMPPQPASSSSY :  19
TaPERK17 : ---------------------------------------------------------------------------------MSGP :   4
TaPERK18 : ---------------------------------------------------------------------------------MASS :   4
TaPERK19 : ----------------------------------------------------------------------------------MS- :   2
TaPERK20 : --------------------------------------------------------------------MPRVFVESVEHLANAPA :  17
TaPERK21 : ------------------------------------------------------------------MGGYADPFMPPQPASSSSY :  19
TaPERK22 : ------------------------------------------------------------------MSSSPPPDAGGSPPVTPSP :  19
TaPERK23 : ------------------------------------------------------------------------------------- :   -
TaPERK24 : ---------------------------------------------------------------------------------MSS- :   3
TaPERK25 : ------------------------------------------------------------------MGGYADPFMPPQPASSSSY :  19
TaPERK26 : ------------------------------------------------------------------------------------- :   -
TaPERK27 : ------------------------------------------------------------------------------------- :   -
TaPERK28 : --------------------------------------------------------------------------MKFMTFGINAL :  11
TaPERK29 : ------------------------------------------------------------------------------------- :   -
TaPERK30 : ------------------------------------------------------------------------------------- :   -
TaPERK31 : ------------------------------------------------------------------------------------- :   -
TaPERK32 : NIDNNDIFKAAIKCEVCTGGNTSVCRITRLKIYALDAVGPIPEELRNLTALTDLDLGQNYLTGPLPSFIGELTAMKSMTFGINAL : 154
TaPERK33 : ----------------------------------------MRTLLQAKGGGRGAGAG----AADAAADAVDTVTG-TTPPPSSTD :  40
TaPERK34 : ----------------------------------------MRTLLQAKGGGRGPGGG-AAEAADEAADAVDTVTGTITPPPSSTD :  44
TaPERK35 : NIDNNDIFKAAIKCEVCTGGNTSVCRITRLKIYALDAVGPIPEELRNLTALTDLDLGQNYLTGPLPSFIGELTDMKFMTFGINAL : 151
TaPERK36 : ---------------------------------------------------------------------MPRLAPSPGSSSEDSS :  16
TaPERK37 : ----------------------------------------MRTLLQAKGGGRGGGGGGGGGAADAAADAVDTVTGTITPPPSSTD :  45
AtPERK1  : ----------------------------------------------------------------------------MSTAPSPG- :   8
AtPERK2  : ----------------------------------------------------------------------------MSSAPPPGG :   9
AtPERK3  : ----------------------------------------------------------------------------MARSNR--- :   6
AtPERK4  : ------------------------------------------------------------------------------------- :   -
AtPERK5  : ------------------------------------------------------------------------------------- :   -
AtPERK6  : ------------------------------------------------------------------------------------- :   -
AtPERK7  : ------------------------------------------------------------------------------------- :   -
AtPERK8  : -------------------------------------------------------------------------MSLVPPLP---- :   8
AtPERK9  : -----------------------------------------------------------------------MATTPVQPP---VS :  11
AtPERK10 : ------------------------------------------------------------------------MTTPAQAPREEVS :  13
AtPERK11 : ------------------------------------------------------------------MDKVQQQADLFGKTISPFV :  19
AtPERK12 : ------------------------------------------------------------------MSDLGESPSSSPPAPPADT :  19
AtPERK13 : ---------------------------------------------------------------------MSDSPTSSPPAPSADS :  16
AtPERK14 : ----------------------------------------------------------------------------MSLSPSSSP :   9
AtPERK15 : ------------------------------------------------------------------------------------- :   -
OsPERK1  : ----------------------------------------------------------------------------------MS- :   2
OsPERK2  : ------------------------------------------------------------------------------------- :   -
OsPERK3  : ------------------------------------------------------------------------------------- :   -
OsPERK4  : ------------------------------------------------------------------------------------- :   -
OsPERK5  : ------------------------------------------------------------------------------------- :   -
OsPERK6  : ------------------------------------------------------------------MSSESDQAPSPSSPSSSSS :  19
OsPERK7  : ------------------------------------------------------------------------------------- :   -
OsPERK8  : ------------------------------------------------------------------------------------- :   -
GmPERK1  : -------------------------------------------------------------------------MSAASPSPATST :  12
GmPERK2  : ------------------------------------------------------------------------------------- :   -
GmPERK3  : ------------------------------------------------------------------------------------- :   -
GmPERK4  : -------------------------------------------------------------------------MSAVSPSPSTST :  12
GmPERK5  : ------------------------------------------------------------------------------------- :   -
GmPERK6  : ---------------------------------------------------------------------------MFAVAYYSPA :  10
GmPERK7  : ---------------------------------------------------------------------------MLAVAPYSPA :  10
GmPERK8  : ----------------------------------------------------------------------------MSTAPAPS- :   8
GmPERK9  : -------------------------------------------------------------------------MTSTNPSPNASP :  12
GmPERK10 : ---------------------------------------------------------------MSTKHTEEDNDSSYLTIPNKDK :  22
GmPERK11 : ----------------------------------------------------------------------------MSTAPAPS- :   8
GmPERK12 : ------------------------------------------------------------------------------------- :   -
GmPERK13 : -------------------------------------------------------------------------MSAPSPS---SS :   9
GmPERK14 : ------------------------------------------------------------------------------------- :   -
GmPERK15 : ------------------------------------------------------------------------------------- :   -
GmPERK16 : -------------------------------------------------------------------------MASANPSPNASP :  12
```

```
                   180       *       200       *       220       *       240       *
TaPERK1  : ------------SPTAAPAPTTPPAPPAN----------------------ATAPPPATPSAPPPAIPSPSPPAPANPPPVSV-- :  51
TaPERK2  : ------------------------------------------------------------------------------------- :   -
TaPERK3  : PSARSRALASASAPAPASSRRPNATPAA------DTAAPSTSAT--------TSPPPSSPPSAPPPKLAAS-PPPASPPP----- :  72
TaPERK4  : ------------SPTAAPAPTTPPAPPAN----------------------ATAPPPATPSAPPPAIPSPSPPAPANPPPASV-- :  51
TaPERK5  : SGPLPKELGNLTDLVSLSVSSNNFSGSLPSELGNLAKLEQLYIDSAGFSGPLPSSLSKLTKMKTLWASDNDFTGQIPDYIGSWN- : 233
TaPERK6  : SGPLPKELGNLTNLVSLGIGSNKFNGSLPLELGNLVKLEQMYIDSAGLSGPLPASLSRLTKMKILWASDNDFTGQIPDYIGSWS- : 232
TaPERK7  : SGPVPKELWNLTDLVSVALGSNHFNGSLPDELGKLTKLQQLYIDSNDFTGPLPEALSQLTNLSTLWASDNSFTGQIPDFLGSLTN : 254
TaPERK8  : SGPLPKELRNLTDLVSLSVSSNNFSGSLPSELGDLAKLEQLYIDSAGFSGPLPSSLSKLTKMKTLWASDNDFTGQIPDYIGTWN- : 233
TaPERK9  : SGPLPKELGNLTNLVSLGIGSNKFNGSLPSELGNLAKLEQMYIDSAGLSGPLPASLSSLTKMKILWASDNDFTGQIPDYIGSWS- : 234
TaPERK10 : SGPLPKELGNLTDLVSLSVSSNNFSGSLPSELGNLAKLEQLYIDSAGFSGPLPSTLSKLTKMKTLWASDNDFTGQIPDYIGSWN- : 233
TaPERK11 : SGPLPKELGNLTNLVSLGIGSNKFNGSLPSELGNLAKLEQMYIDSAGLTGPLPASLSKLTKMKILWASDNDFTGQIPDYIGSWS- : 143
TaPERK12 : SGPVPKELWNLTDLVSLALGSNHFNGSLPDELGKLTKLQQLYIDSNDFTGPLPAALSQLTNLSTLWASDNSFTGQIPDFLGSLTN : 253
TaPERK13 : PSPSPGATKASPADSVAPATSSPFSPAP------VKLPNDTPAD--------PPAAPPAPSAAVPPQTPES-PPPPSPSP----- :  69
TaPERK14 : ------------SPPAAPGPASPPGNRT-----------------------APPPASAPPATNSTPPSPPAP-----PPSSPL-- :  45
TaPERK15 : VGGPPPPPPKSSASPPTPARSSPPPPTP--------PPKSPPPS---------SPPPASSGNNQSANLPPGSPPAKKSPAS---- :  81
TaPERK16 : AVPQGGHGQPQP--APRPPGCPYSSSAS--------APPVSTSY--------HSLPPAASPPPVSS-PPPASPPPEPLPS----- :  80
TaPERK17 : SPPVTPVS----PPAPDAGETPRVTPAPTPPVVTPVSPPPPDA---------AGTPPPPDASGTPPVTPPPPDAAATPPPPVNPP :  76
TaPERK18 : PSPSPGATKASPAASVAPATSSPASPAP------VKLPNETPAD--------PPAAPPAPSAAVPPQTPES-PPPPSPSP----- :  69
TaPERK19 : ------------SPPAAPGPASPPGNRT-----------------------APPPASAPPATNSTPPSPPAPAASSPPPSSPL-- :  50
TaPERK20 : VGGPPAPPPQPSASPPPQARPSPPPPTP--------PPKSPPPS---------SPPPASSGNNQSPNLPPGSPPPEKSPAS---- :  81
TaPERK21 : AVPQGGHGQPQPQPAPRPPGCPYSSSAS--------APPVSTSY--------HSLPPAASPPPVSS-PPPASPPPETLPS----- :  82
TaPERK22 : TPPVTPVTPSPTPPVTPVTPSPPDNPVVTPPVAPVNSPPPPDTSMFNPPPPVTPSPPPPEATPPPPVTPSPPPPVAIPPTPVTPS : 104
TaPERK23 : ------------------------------------------------------------------------------------- :   -
TaPERK24 : ------------SPPAAPGPASPPGNRT-----------------------APPPASAPPATNSTPPSPPAPPASAPPPSSPV-- :  51
TaPERK25 : AVPQGGHGQPQPQPAPRPPGCPYSSSAS--------APPVSTSY--------HSLPPAASPPPVSS-PPPASPPPEPLPS----- :  82
TaPERK26 : ------------------------------------------------------------------------------------- :   -
TaPERK27 : ----------------MDDSPLSRGPLGPLLGSGPLDPSS-SAADSDGSGGSSSADSDSSGGS----SSSGSPPSPSSPQ----- :  59
TaPERK28 : SGPLPKELGNLKNLIKLGLGGNNFSGSLPSELGNLAKLEELYIDSSGLSGPLPSSLSQLTKMKKLWASDNDFTGQIPDYIGTWSS :  96
TaPERK29 : ----------------MDDSPLSRGPLAPLLGSGPLDPSSSAAADSDGSGGSSSGASDSSGES----SSSGSPPSPSGPQ----- :  60
TaPERK30 : ------------------------------------------------------------------MSSNSSSAAPPPPS----- :  14
TaPERK31 : ------------------------------------------------------------------MSSNSSSAAPPPPS----- :  14
TaPERK32 : SGPVPKELGNLKNLIKLGLGGNNFSGSLPTELGNLAKLEELYIDSSGLSGPLPSSLFQLTKMKKLWASDNDFTGKIPDYIGSWSS : 239
TaPERK33 : DSPPPPASD----PPPSPSSSSSPPPPS------PSSPSDPPPS--------PSSPPPSPPLAPPPSPAASSPPPPEVSP----- : 102
TaPERK34 : DSPPPPTPDSSSDPPPSPSSSSSPPPPS------PSAPSDPPPS--------PSSPPPSQPLAPPPSPAASSPPPPEALP----- : 110
TaPERK35 : SGPVPKELGNLKNLIKLGLGGNNFSGSLPSELGNLAKLEELYIDSSGLSGPLPSSLSQLTKMKKVWASDNDFTGQIPDYIGSWSS : 236
TaPERK36 : DDRSDGSKSKSKSKAPPPRRSSRSSGSSSDENGDDSSSPSPPSS---DDEDGGSRSSSSSSDSQDSSPSDSKSSQSSRRS----- :  93
TaPERK37 : DSPPPPASDSSSDPPPSPSSSSSPPPPT------PSSPSDPPPA--------PSSPPPSPPLAPPPSPAASSPPPPEALP----- : 111
AtPERK1  : ------------TTPSPSPPSPPTNSTTT----------------------TPPPAASSPPPTTTPSSPPPSPSTNSTS------ :  53
AtPERK2  : TPSPPPQPLPIPPPPQPLPVTPPPPPTAL----------------------PPALPPPPPPTALPPALPPPPPPTTVPPIPPSTP :  72
AtPERK3  : ------------CVPQNSSIVQIPIEEVF----------------------KTQLKSRWQQITMSSASSPPPPQVFVP------- :  50
AtPERK4  : ----------------MAS-SPESAPP----TNSTSSPSPPSNTN----------STTSSPPA----PSPPSPTPPQGDS----- :  45
AtPERK5  : ----------------MADSPVDSSPAPETSNGTPPSNGTSPSNESSPPTPPSSPPPSSISAPPPDISASFSPPPAPPTQ----- :  64
AtPERK6  : ----------------MAEGQSPENSPPSP---TPPSPSSSDNQQQSSPPPSDSSSPSPPAPPPPDDSSNGSPQPPSSDS----- :  61
AtPERK7  : ----------------MAEGQSPENSPPAPPPPSPPSPPSSNDQQTTSPPPSDNQETTSPPPP----SSPDIAPPPQQQQ----- :  60
AtPERK8  : ---------ILSPPSS-----NSSTTAP--------PPLQTQPT-------TPSAPPPVTP--PPS--PPQSPPPVVSSS----- :  55
AtPERK9  : NSPPVTSP-----PPPLN-----NATSP------ATPPPVTSPL-------PPSAPPPNRAPPPPP-PVTTSPPPVANGA----- :  67
AtPERK10 : LSPSLASPPLMALPPPQPSFPGDNATSPTREPTNGNPPETTNTP-------AQSSPPPETPLSSPP-PEPSPPSPSLTGP----- :  85
AtPERK11 : ASQP-TNVGGFTDQKIIGGSETTQPPATSPP----SPPSPDTQT------SPPPATAAQPPPNQPP---NTTPPPTPPSS----- :  85
AtPERK12 : APPPETPSENSALPP-VDSSPP-SPPADSSS----TPPLSEPST------PPPDSQLPPLPSILPP--LTDSPPPPSDSS----- :  85
AtPERK13 : APPPDTSSDGSAAPPPTDSAPPPSPPADSS------PPPALPSL------PPAVFSPPPTVSSPPPPPLDSSPPPPPDLT----- :  84
AtPERK14 : APATSPPAMSLPPADSVPDTSSPPAPPLSPLPPPLSSPPPLPSPPPLSAPTASPPPLPVESPPSPPIESPPPPLLESPPPPPLES :  94
AtPERK15 : -----------MSTDTIPSLSSPPAPEF--------------------------------------------------------- :  17
OsPERK1  : ------------SPSSPP-----PANQT-----------------------ATP----PPANQTAAP------------------ :  25
OsPERK2  : ------------MWARFWYSKPAERARP------------------------AAFVPPPPPPTPPQYVPPEEPSAFAKLYA---- :  45
OsPERK3  : ------------------------MARP------------------------AAFVPPPP-----QYVPPEEPSAFARLY----- :  27
OsPERK4  : -----------------------------------------------------------------------MSIVPPP------- :   7
OsPERK5  : ------------------------------------------------------------------------------------- :   -
OsPERK6  : SSSGNGS-----NKAPPPEESDNSSS-----NGSSSSSPTPPSSQSSDSDSGGGSSSPSQGSSSPSPPPPSGSSSESHSS----- :  89
OsPERK7  : ------------------------------------------------------------------------------------- :   -
OsPERK8  : --------------------------------------------------MAASPESSPSSAN----STTPSAPSPSSSL----- :  26
GmPERK1  : AAPPLQTPPSSNNTSPSPSTA--TTPPLQQTPPSTSPPQQPASP-------PPSTPSPANPPPSSP---PSSPPPPMSGT----- :  80
GmPERK2  : ----------------MSS-PHNSSPD-----NETPDNSTPDDADNSSSPSSQSPPPSSPPPS----SPPPSSPPPSSPP----- :  54
GmPERK3  : ----------------MSS-PHNSAPH-----NSSPDNSTPDD---------------ATPNN----SIPDNSTPDNSIP----- :  39
GmPERK4  : AAPPPQTSPSANTTSPPPSTV--TTPPPQQTPSSTSSPQQPASP-------PPTTPSPANPPPSPPSPPPSSPPPSMSGT----- :  83
GmPERK5  : ----------------MASNSDKDSP-------TPPSPDSS-SSQSPPSSPPPSQDSSSPPPS----SQDNSSPPPSSPP----- :  52
GmPERK6  : ASPPLPP-FDITPPPSPSMYPTPPAPPVT--------PPP-----------SAVTPLPLTEPLLP---SPSPVSLPPPFP----- :  67
GmPERK7  : APPPLSLLFDITPPPSPSMYPTPPAPPVT--------PPPETLLVQDSPPPSSVTPLPLTEPLLP---SPSPESLPPPLPSSTVT :  84
GmPERK8  : ------------SPPPTNGTAPPPSTPSA----------------------PPPATPSAPPPSTPSSPPPATPSSPPPATPSS-- :  57
GmPERK9  : VAVPPALGGILSPPPSSSSPTNSSKSPS--------PPNSPQPN----QTQTPNSPAPSSPSAPPP--SPPSPPQAVPVT----- :  78
GmPERK10 : RKQKSSHDNDKESAPSPKPPDLDTQESAPSN----SPPPSPPPP------PPQLKLPILSPPRLKPSSISLLSPPSKDLSGSPSW :  97
GmPERK11 : ------------SPPANNGTAPPPSTP---------------------------ATPSAPPPATPSAPPPSTPSSPPPATPSS-- :  52
GmPERK12 : ----------------MSS-PHNSSPDDSSPDNATPDNSTPDDSDNSSPPS-QSPPPSSPPPS----SPPPSSPPPSSPP----- :  58
GmPERK13 : PAPP---------------------------------PELSSSA-------PPSSTSPLPLP---------SPSPSLS------- :  38
GmPERK14 : ----------------MSS-PHNSAPH-----NSTPDNSSPDD---------------ATPDN----SIPDNSTPDDATP----- :  39
GmPERK15 : ----------------------------------------------------------MSPP------------PSKYLS----- :  10
GmPERK16 : VAVPPALGAILSPPPSSSSPTNTSTPPS--------PPSSSQPNQTQTQTQTPNSPAPSSPSDPSA--PPPSPPQAVPLT----- :  82
```

```
               260         *       280         *       300         *       320         *       340
TaPERK1    : -----------------------------PPPAAPAASPP------------------------------------APSSTPATP :   71
TaPERK2    : ------------------------------------------------------------------------------------- :    -
TaPERK3    : --TP-PAALPTPKP-----------APSTSTPPPTTTSPPAPPPKRSP------------------------AAAPPPTAPAPRP :  119
TaPERK4    : -----------------------------PPPAAPAASPP------------------------------------APSSTPATP :   71
TaPERK5    : LTDLRFQGNSFQGPLPATLSNLVQLTNLRIGDILNGSSSSLAFISNMTSLNSLVLRNCRISDTLLSVNFSKFTSLNLLDLSFNNI :  318
TaPERK6    : LTELRFQGNSFEGPIPATLSNLAQLTSLRIGDILNGSSSSLAFVNNMTSLSTLVLRNCRISDKLSSIDFSKLTSLNLLDLSFNNI :  317
TaPERK7    : MTQLRLQGNSFQGPIPRSLSNLIKLTSLRIGDIVNGSSS-MEFVGNMTSLGELVLRNSKISDTLASVDFSKFVNLTLLDLSFNNI :  338
TaPERK8    : LTDLRFQGNSFQGPLPATLSNLIQLTSLRIGDIVNGSSSSLAFISNMTSLNTLVLRNCRISDTLLSVNFSKFTSLNLLDLSFNNI :  318
TaPERK9    : LTELRFQGNSFEGPIPATLSNLAQLTSLRIGDILNGSSSSLAFVNNMTSLSTLVLRNCRISDKLSSIDFSKLTSLDLLDLSFNNI :  319
TaPERK10   : LTDLRFQGNSFQGPLPATLSNLVQLTSLRIGDIVNGSSSSLAFISNMTSLNTLVLRNCRISDTLLSVNFSKFTSLNLLDLSFNNI :  318
TaPERK11   : LTELRFQGNSFEGPIPATLSNLAQLTSLRIGDILNGSSSSLAFVNNMTSLSTLVLRNCRISDKLSSIDFSKLTSLNLLDLSFNNI :  228
TaPERK12   : MTQLRLQGNSFQGPIPRSLSNLIKLTSLRIGDIVNGSSS-MAFVGNMTSLGELVLRNSKISDTLASVDFSKFVNLTLLDLSFNNI :  337
TaPERK13   : --PPDPVASPPPVP-----------VASPPPTP-PATMPPAQVPPPPA------------------------PAADPPKQSPLRP :  116
TaPERK14   : -----------------------------PPPSTPAATPP------------------------------------SPGATPATP :   65
TaPERK15   : --SPPPPASPPPST------------PPASRSPPPSSPPPSPPSQ----------------------------QSSPPPAQSTPP :  124
TaPERK16   : --PPPALPSSPPPPALS---------PPPPDAPPPSLPPPSPSPS----------------------------PPPAEVQAPPPP :  126
TaPERK17   : PPD---------------------GASTPPP----------------------------------VNPPVNPPPPPRPTP----- :  101
TaPERK18   : --PPDPTASPPPVP-----------VASPPPAALPPPSPPTAVPPPPA------------------------PAADPPKQSPLRP :  117
TaPERK19   : -----------------------------PPPSTPAAPPP------------------------------------SSGTTPATP :   70
TaPERK20   : --SPPPPASPPPST------------PPASRSPPPSSPPPSPPSQ----------------------------QSSPPPAQSTPP :  124
TaPERK21   : --PPPALPSSPPPPALS---------PPPPDAPPPSLPPPSPSPS----------------------------PPPAEVQAPPPP :  128
TaPERK22   : PPPPPPVAIPPSP----------VTAPPPPPPVAIPPSLPPQVAAVPPTTAKPPTIVNPPAPEPSVQPPVNSPPPPPPSPPPPRP :  179
TaPERK23   : ------------------------------------------------------------------------------------- :    -
TaPERK24   : -----------------------------PPPSTPAAPPP------------------------------------SSGTTPATP :   71
TaPERK25   : --PPPALPSSPPPPALS---------PPPPDAPPPSLPPPSPSPS----------------------------PPPAEVQAPPPP :  128
TaPERK26   : ------------------------------------------------------------------------------------- :    -
TaPERK27   : -------------------------------------------------------------------------SST--QSTPPPG :   69
TaPERK28   : LTELRFQGNSFQGPIPATLSNLGQLASLRIGDILNGSSSSLAFISNLTSLNTLVLRNCRISDKLVSIDFSKFASLNLLDLSFNNI :  181
TaPERK29   : -------------------------------------------------------------------------SST--QSTPPPG :   70
TaPERK30   : -------------------------------------------------------------------------KSSPPPSAAPPP :   26
TaPERK31   : -------------------------------------------------------------------------KSSPPPSAAQPP :   26
TaPERK32   : LTELRFQGNSFQGPIPATLSNLSQLASLRIGDILSGSSSSLAFINNLTSLNTLVLRNCRISDKLVSIDFSKFTSLNLLDLSFNNI :  324
TaPERK33   : --PPPPVASPPPPPDAFPPPPPSPVSAAPPPAD-AAPPPPDNAPLPPD------------------------NAAAPPPSPTQAA :  160
TaPERK34   : --PPPPVASPPPPPDALPPPPPSPVSAAPPPADTAAPPPPDNAPPPPD------------------------NAAAPPTSPTQAP :  169
TaPERK35   : LTELRLQGNSFQGPIPATLSNLGQLASLRIGDILNGSSSSLAFVNNLTSLNTLVLRNCRISDKLVSIDFSKFTSLNLLDLSFNNI :  321
TaPERK36   : --PPPSKSKSSPSPSSS-----PSRPSSSRNGGKSDSSPPPPTDESSG------------------DGEDTDSSPPPKRSSPPEQ :  153
TaPERK37   : --PPPPVASPPPPPDALPPPPPSPVSAAPPPAD-AAPPPPDNTPPPPD------------------------NAAAPPPSSTQAP :  169
AtPERK1    : -----------------------------PPPSSPLPP-------------------------------------SLPPPSPPGS :   72
AtPERK2    : SPPPPLTPSPLPPSPTTPSPPLTPSPTTPSPPLTPSPPPAITPSPPLTPSPLPPSPTTPSPPPPSPSIPSPPLTPSPPPSSPLRP :  157
AtPERK3    : ------------------------------EPLFSEPP--------------------------------------PPPKAPVNV :   67
AtPERK4    : -------------------------------------------------------------------------SSSPPPDSTSPP :   57
AtPERK5    : -------------------------------------------------------------------------ETSPPTSPSSSP :   76
AtPERK6    : -------------------------------------------------------------------------QSPPSPQGNNNN :   73
AtPERK7    : -------------------------------------------------------------------------ESPPPPLPENSS :   72
AtPERK8    : --PPPPVVSSPPPS-----------SSPPPSPPVITSPPPTVASS----------------------------PPPPVVIASPPP :   99
AtPERK9    : --PPPPLPK--PPESSS--PPPQPVIPSPP--PSTSPPPQPVIPSPPP-----SASPP--PALVPPLPS---SPPPPASVPPPRP :  134
AtPERK10   : --PPTTIPVSPPPEPSP--PPPLPTEAPPPANPVSSPPPESSPPPPPP-----TEAPPTTPITSPSPPT---NPPPPPESPPSLP :  158
AtPERK11   : ---PPPSITPPPSP--------------PQPQPPPQSTPTGDSPVVIP----------------------FPKPQLPPPSLFPPP :  131
AtPERK12   : ---PPVDSTPSP----------------PPPTSNESPSPPEDSETPP-------------------------APPNESNDNNPPP :  126
AtPERK13   : ---PPPSSPPPPDA--------------PPPIPIVFP-PPIDS------------------------------PPPES-TNSPPP :  120
AtPERK14   : PSPPSPH----------------VSAPSGSPPLPFLPAKPSPPPS--------------SPPSETVPPGNTISPPPRSLPSESTP :  149
AtPERK15   : -----------------------------------------------------------------------------PSTTPDTA :   25
OsPERK1    : -----------------------------PPASNNSSSPP------------------------------------APG-SLSPP :   44
OsPERK2    : ----------------------------------------------------------------------------VAGDVVGRA :   54
OsPERK3    : -------------------------------------------------------------------------------DVVDRA :   33
OsPERK4    : -------------------------------------------------------------------------KSSPPLMAAS-P :   18
OsPERK5    : ------------------------------------------------------------------------------------- :    -
OsPERK6    : --PPPAPPQSSSSSSSS-----SSSGGGSKSSPEAPSPPSESSGNGGG------------------GGGGGRSSPPPNWSPPPQQ :  149
OsPERK7    : ------------------------------------------------------------------------------------- :    -
OsPERK8    : -------------------------------------------------------------------------SKS--PSPPSP- :   35
GmPERK1    : --PPPLVPPPSPPPSPP----PSTPAAQPAVPPSSPPPPPSPSPSVP------SSPPPSNPRNNTSPPP---SPPPQPPAPPPQP :  150
GmPERK2    : -------------------------------------------------------------------------PSSPPPSSS--S :   64
GmPERK3    : -------------------------------------------------------------------------DNSTPDNSTSNK :   51
GmPERK4    : --PPPLVPPPSPPPSPPSSPPPSSPAAPPPVPPSSPPPP-SPSAPVPPNPPNNTSPPPSQPPQSPAPPPQPPAPPPQPPAPPPQP :  165
GmPERK5    : -------------------------------------------------------------------------PSSPPPS-SSPP :   63
GmPERK6    : LSPSTP------------------TAASQPPPLPFATA----------------------PATTTFPPFAVALSPP--TPTATFP :  110
GmPERK7    : PSPPTP------------------TAESQPPPFPFATA----------------------PAPTTFPPFAVAPSPP--TPTATFP :  127
GmPERK8    : -----------------------------PPPATPSSPPPS----------------------------------TPSSPPPATP :   79
GmPERK9    : --PPPSISLSPPP------------TLPPPSPP--DSPPPLPPAS----------------------------PPSPPATTAPPP :  119
GmPERK10   : SPPPPPSRSPSPPS--------------SLSLLSSSSPPPSPS-------------------------------SPPPPPSSPPP :  137
GmPERK11   : -----------------------------PPPATPSSPPPA----------------------------------TPSSPPPATP :   74
GmPERK12   : -------------------------------------------------------------------------PSSPPPSSPPPS :   70
GmPERK13   : --------------------------------PAN-PLP---------------------------------APPPQSPASISSP :   57
GmPERK14   : -------------------------------------------------------------------------DNSIPDN----- :   46
GmPERK15   : ---PPPSPRPPP-----------------LPLPSPSLRPPSPL-------------------------------PPSPP---PPS :   41
GmPERK16   : --PPPSISLSPPPSSTP------PPTLPPPSPP--DSPPPLPPASPTP-----------PVTTSPPSPPVTTSPPSPPATTSPPP :  146
```

```
                        *        360         *        380         *        400         *        420
TaPERK1   : SAPSPSPPATPSPPSDTPSS---------PSPEGRSPPSPAGGGRSPSTPGHNNP----------------SPKSPPHSSGGGGG :  131
TaPERK2   : ------------------------------------------------------------------------------------- :    -
TaPERK3   : TPPAPAPRTAPTSQSPAASPPAHAAPHAKPSPPPAAGHHPQLPTPATAVDPAN-----------------QHGRSPNKSS----- :  182
TaPERK4   : SAPSPSPPATPSPPSDTPSP---------PSPEGRAPPSPGGGGRSPFTPGHNNP----------------SPKSPPHSSGGG-- :  129
TaPERK5   : TGQVPQTLLNLNSLGFLFLGNNSLSGSLPSSVGPMLKNLDFSYNQLTGNVPSWG---NSQLNLVANNFGADISSNSGLPTGLDCL :  400
TaPERK6   : TGQVPQTLLNLKLINFLFLGNNSLSGNLPSSIGSSLKNLDFSYNQLSGTVPSWAK--DSQLNLVTNNFVADSSSNSVLPTGWGCL :  400
TaPERK7   : TGQMPRSIFDLPMLSYLFLGNNSLSGSLPATKSPLLANLDFSYNHLSGSFPSWVTQKNLQINLVANDFVIDSSNNSVLPFGLNCL :  423
TaPERK8   : TGQVPQTLLNLNSLGFLFLGNNSLSGSLPSSVGSMLKNLDFSYNQLTGSVPSWAR--NSQLNLVANNFGADISSNSALPTGLDCL :  401
TaPERK9   : TGQVPQTLLNLNSLKFLFLGNNSLSGNLPSSIGSSLKNLDFSYNQLSGTVPSWAK--DSQLNLVTNNFVADSSSNSVLPTGWGCL :  402
TaPERK10  : TGQVPQTLLNLNSLGFLFLGNNSLSGSLPSSVGPMLKNLDFSYNQLTGSVPSWAR--NSQLNLVANNFGADISSNSALPTGLDCL :  401
TaPERK11  : TGQVPQTLLNLKLINFLFLGNNSLSGNLPSSIGSSLKNLDFSYNQLSGTVPSWAK--DSQLNLVTNNFVADSSSNSVLPTGWGCL :  311
TaPERK12  : TGQMPQSIFDLPMLSYLFLGNNSLSGSLPATKSPLLANLDFSYNHLSGSFPSWVSQKNLQINLVANDFVIDSSNNSVLPFGLNCL :  422
TaPERK13  : PAPAASPPPPKSTSPPPSPPDRSTAPPVPRSPPPRHPGSPPPPTEPQAPPPSV-----------------SPIIKPPTSP----- :  179
TaPERK14  : SLPPPTAPGVPAAPSPPSTT---------PSPPTDRPTPSAQASPPPPT------------------------------------ :  105
TaPERK15  : SPGVSTRPSPGATTSPSQQSPPKATTSPPASPPPSQPKNPAPATPTPSPPLNA------------------KSATKPPAG----- :  186
TaPERK16  : MAADQPRVQPRVYPSPPPPSLPPPPPVVLSPPAPPPS-----HPPSPSPTPAP---------------------APAPTP----- :  180
TaPERK17  : --TPNPPPVTPSPPPPRPTPTPPNPTPVTPSPPPPRPTPTPTPTPT--PSRPPPS----------------SPPPPTNVPRSSPP :  166
TaPERK18  : PAPAASPPPPKTTSPPPSPPDRSTAPPVARSPPPRHPGSPPPPTEPQAPPPSV-----------------SPIIKPPTSP----- :  180
TaPERK19  : SLPPPTAPGAPAAPSPPSTT---------PSPPTDRPTPSAQASPPPP------------------------------------- :  109
TaPERK20  : SPGVSTRPSPGATTSPSQQSPPKATTSPPASPSPSQPKNPAPATPTPSPPLNA------------------ESATKPPTG----- :  186
TaPERK21  : MAADQPRVQPRVYPSPPPPSLPPPPPVVLSPPAPPPS-----HPPSPSPTPAP---------------------APAPTP----- :  182
TaPERK22  : PTRPNPTPVTPSPPPPRTTPTP-NPTPATPSPPPPRPTPTPTPTPTPTPPRPPPS----------------SPAPPTDVPRSFPP :  247
TaPERK23  : ------------------------------------------------------------------------------------- :    -
TaPERK24  : SLPPPTAPGAPAAPSPPSTT---------PSPPTDRPTPSAQASPPPP------------------------------------- :  110
TaPERK25  : XXXXXXXXXXXVYPSPPPPSLPPPPPVVLSPPAPPPS-----HPPSPSPTPAP---------------------APAPTP----- :  182
TaPERK26  : ------------------------------------------------------------------------------------- :    -
TaPERK27  : SSDSAPSPPSPSQSPPPATPAGTSGPPPAQVSPPGSNAIPSPQAPKSNGGGGGGGG----------------------------- :  125
TaPERK28  : TGQVPQTLLNLNSLAFLFLGNNSLSGSLPSSVGSLLKTLDFSYNQLSGSIPSWAK--NSQLNLVANNFVADSSSNSVLPAGWGCL :  264
TaPERK29  : SEESAPSPPSPSLSPPPATPAGTSGSPPGQSSPPGSNAIPSPQAPKSNGGGGG-------------------------------- :  123
TaPERK30  : SP----SISNSSSPAPSSSNDPSPPPAPPKSNGAPAS-PARVPSPPAQSS----------------------------------- :   71
TaPERK31  : SPGSSPSTSNSSSPAPSSSNDPSPPPAPPKSRGAPVP-PGRNPSPPAQPS----------------------------------- :   75
TaPERK32  : TGQVPQTLLNLNSLAFLFLGNNSLSGSLPSSVGPLLKNLDFSYNQLSGSIPSWAK--DSQLNLVANNFVADSSSNSVLPAGWGCL :  407
TaPERK33  : PPTQSQAKPPSSKASPPPAAEEPTASPVVDATPPPKQPAVDYAPPPSARTSSS-----------------ATAHSPPASA----- :  223
TaPERK34  : PPSQSQAKPPSSKASPPPAAEEPTASPVVNATPPPKQSAVDYAPPPSARTSSS-----------------ATAHSPPASA----- :  232
TaPERK35  : TGQVPQTLLNLNSLAFLFLGNNSLSGSLPSSVGPLLKNLDFSYNLLSGSIPSWAK--NSQLNLVANNFVADSSSNSVLPAGWGCL :  404
TaPERK36  : SEDSEPSPSSSSSRSPPPRSSPPPPPGWNGNVDQAGSSPPDGSQPPPSPSRSPP------------------PPPAPANHSVVFI :  220
TaPERK37  : PPSQSQAKPPSSKASPPPAAEEPTASPVVDATPPPKKSAVDYAPPPSARTSSS-----------------ATAHSPPASA----- :  232
AtPERK1   : LTPPLPQPSPSAPITPSPPSPTTPSNPRSPPSPNQGPPNTPSG-STPRTPSNTK-----------------PSPPSDS------- :  132
AtPERK2   : SSPPPPSPATPSTPPRSPPPPSTPTPPPRVGSLSPPPPASPSGGRSPSTPSTTPG----------------SSPPAQS------- :  219
AtPERK3   : SLSPPP-------PPRSP---STSTPP-RLGNRNPPPPASPSG-QEPTTPTMTPG----------------FSLSPPS------- :  117
AtPERK4   : APQAPNPPNSSNNSPS-PPSQGGGGERGNGGNNGGNDTPPSRGSPPSPPSRSNGDN----------------GGSRSS------- :  118
AtPERK5   : PVVANPSPQTPENPSPPAPEGSTPVTPPAPPQTP--SNQSPERPTPPSPGANDDRNRTNGGNNNR----DGSTPSPPSSGN---- :  151
AtPERK6   : DGNNGNNNNDNNNNNNGNNNNDNNNGNNKDNNNNGNNNNGNNNNGNDNNGNNNNGNNNDNNNQNNGGGSNNRSPPPPS------- :  151
AtPERK7   : DGSSSSSPPPPSDSSSQSQSPPPPSTSPPQQS----DNNGNKGNNNENN-KGNDGSSGDGGNKN-----MSHTPPPPS------- :  140
AtPERK8   : STPATTPPAPPQTVSPPPPPDASPSPPAPTTTNPPPK----PSPSPPGETPSP-----PGETPSP----PKPSPSTPTP------ :  165
AtPERK9   : SPSPPILVRSPPPSVRPIQSPP-PPPSDRPTQSPPPP--SPPSPPSERPTQSP-----PSPPS--------ERPTQSPPP----- :  198
AtPERK10  : APDPPSNPLPPPKLVPPSHSPPRHLPSPPASEIPPPPRHLPSPPASERPSTPPSDSEHPSPPPPG----HPKRREQPPPPGSKRP :  239
AtPERK11  : SLVNQLPDPRPNDNNILEPINNPISLPSPPSTPFSPPSQENSGSQGSPPLSSL------------------LPPMLPLNPN---- :  194
AtPERK12  : SQDLQSPPPSSPSPNVGPTNPESPPLQSPPAPPASDPTNSPPASPLDPTNPPP------------------IQPSGPATS----- :  188
AtPERK13  : PEVFEPPPPPADEDESPPAPPPPEQLPPPASSPQGGPKKPKKHHPGPATSPP--------------------APSAPATS----- :  180
AtPERK14  : PVNTASPP-PPSPPRRRSGPKPSFPPPINSSPPNPSPNTPSLPETSPPPKPPLST----------------TPFPSSSTPPPKKS :  217
AtPERK15  : TSPAPSQPSIIGPSSLAPFP--------------ETTTNIDGGS----------------------------------------- :   55
OsPERK1   : SLTP------PAAPSPPSTT---------PSS----PAAAAAAS----------------------------------------- :   69
OsPERK2   : KALLT--TGGP-------VTPSDDGQRVRRALAQLT--APPSDPAPAAPQKDS-------------------------------- :   96
OsPERK3   : KAALVTITGGPTTVHGVVVISSGHGQRARRAVTEIITTAPASDPAPAAPQTGS-------------------------------- :   86
OsPERK4   : SP---TNSSSSSSPAPSVSPPPAPSNPHGGGGGAPPPSPARVPSPPSRSSGGG-------------------------------- :   68
OsPERK5   : ------------------------------------------------------------------------------------- :    -
OsPERK6   : QQQHQ---SGGSTPSPPPSPSSNQPPPSSGSSASSSEPSPPRSSPPPSPPQSSGG--------------NNGQPPKPSGGQQQAP :  217
OsPERK7   : -------MADFYSP----------------------------------------------------------------------- :    7
OsPERK8   : ---SSPPPPASTNAPPKSAGGVSSSTQAQPSSESSSESTTTPSAPAER------------------------------------- :   80
GmPERK1   : PAPPANNTSPPPQRNSPPPPAATPPRTSPPQTSPSPP----AAPPPRNSTRLPPPVNSPPPPVDA----PPPRSSSPPAP----- :  222
GmPERK2   : SPPPSSPPPSSSSSPP-SPSPPTSNNPSPPSPNGSSQSPSPPGSSRSPPSFE--------------------SPSPPH------- :  121
GmPERK3   : SAPHNSTPDNA-----------TPDNSTPNNSNKSHKS-----SSVSPPSME--------------------GQSTPT------- :   93
GmPERK4   : PAPPPNNTSPSPPRNSPPSPAATPPRASPPQNSPSPP----AVPPPRNSTRSPPSANSPPPPVNA----PPPRSSAPPAP----- :  237
GmPERK5   : PSPPPPSSNDQKSPPPPSDSSSSPPSPPPPPPPD--KDSGSHRSPPPPPHSRHEFS----------------PPPPPN------- :  123
GmPERK6   : PALPEMPSRPPATVATVTVP-----PPFMTEPTLVTPWFPALQSSAPEVN-----------------------LPSTTR------ :  161
GmPERK7   : PALPEMP---PATAATVTVPSPPQHPPFMTEPTLATPWFPALPASAPEVN-----------------------FPSTAR------ :  180
GmPERK8   : SASPPSTPSASPPPSTPTTPSTSPPSTSPPSPPSHSPPSPPSGGGGGSTPSPPSR----------------SSPSPPSGSRPTTP :  148
GmPERK9   : ---AETPPSLPN-LSPPSPPDGSPPPQSPPTTIPPPSRPVSPSPPPPANVPRPPSTSTPPQKQSP----PKTTPSHASPP----- :  191
GmPERK10  : PPPPSSPPPPPSSPAPPQSVPLRN-NGNPSPPPPYYPRKQ---------SPPQ------------------FHAPPPPS------ :  188
GmPERK11  : SSPPPSTPSASPPPSTPTTPSTSPP-----SPPSTSPPSPPSG---GSTPSPPSR----------------SSPSPPSGSSPTTP :  135
GmPERK12  : SPPPLSPPPSSSSSPPPSPSPPTSNSPSPPSPNGSSQSPSPPGSSHSPPSFE--------------------SPSPPH------- :  128
GmPERK13  : PA-----TLPPPHSSSP----------SPPSTTTISS----SAPPP--------------------------------------- :   84
GmPERK14  : -----FTPDNS-----------TPDNSTPDNSNGSHKS-----SSMSPPSME--------------------RHNTPT------- :   83
GmPERK15  : LPPPSSPPPPQS-----QSVPLRNGNPSPPPPPPYYPRKQWPPPLQPLSSPPQ------------------FRAPPPPDR----- :   98
GmPERK16  : ---AETPPSLPN-LSPPSPPAGSPPPQSPPTTIPPPSRPISPSPPPPANFPRPPTTRTPPEKENP----PKTTPSHASPP----- :  218
```

```
                *       440       *       460       *       480       *       500       *
TaPERK1   : G----GGSGVSTSVVVGVAVGGFVLLLLATFVCLCCLRKKRRRQ------------------------PPPPHYGY-------PP :  181
TaPERK2   : ---------------------------------MFCLT----------------------------------------------- :    5
TaPERK3   : --TQSAGERGRKASPSPGSSNGGASNGVVI--AVGAVLAFLVVTLIAAAVLYTKKRRRGRDDYRAGFRSPPYSSELP-------- :  255
TaPERK4   : ------GSGVSTSVVVGVAVGGFVLLLLATFVCLCCLRKKRRRQ------------------------PPPPHYGY-------PP :  177
TaPERK5   : QRNTPCFFGSPKSSSFAVDCGSDRPISGSDNSLYQPDAATLGAASYYVTGEPTWGASNVGRFMDASNGSSIIYSSHQFLNTLDTE :  485
TaPERK6   : QRNTPCFLGLPHSSSFAVDSGSRRPVSGSDNSFYESDDASLGPASFYITGAQTWGVSNVGRFMDAQNGSYIIYSSRQFLNTLDTE :  485
TaPERK7   : QRNTPCSLGSPHSSSLAVDCGGSRTISGSDNAMYQADNANLGAASYYVGGAPIWGVSSSGRFMDPPNGSYIIYSSRQFQNTLDSG :  508
TaPERK8   : QRNTPCFLGSPKSSSFAVDCGSDRPISGSDNSLYEPDAVTLGAASYYVTGEPTWGASNVGRFMDASNGSSIIYSSHQFLNTLDTE :  486
TaPERK9   : QRNTPCFLGLPQSSSFAVDSGSRRPVSGSDNSFYESDDASLGPASFYVTGAQTWGVSNVGRFMDAQNGSYIIYSSRQFLNTLDTE :  487
TaPERK10  : QRNTPCFLGSPKSSSFAVDCGSDRPISGSDNSLYQPDAATLGAASYYVTGEPTWGASNVGRFMDASNVSSIIYSSHQFLNTLDTE :  486
TaPERK11  : QRNTPCFLGSPQSSSFAVDSGSRRPVSGSDNSFYESDDASLGPASFYVTGAQTWGVSNVGRFMDAQNGSYIIYSSRQFLNTLDTE :  396
TaPERK12  : QRNTPCSLGSPQSSSLAVDCGGSRTISGSDNAMYQADNANLGAASHYVGGAPIWGVSSSGRFMDPPNGSYIIYSSRQFQNTLDSG :  507
TaPERK13  : --SPTPVDPTTPTAPTTPSPPSTPGFGPPS--VPTSPTTTPPTPIAPN-------------------IPQVPSWQDS-------- :  233
TaPERK14  : ------SSALNTATVAGIAVGGLIALLLASLLCFCMFKKKRRHHPHHPPP------------------PPPPHHLHYYGHPPPPP :  166
TaPERK15  : ----AGTSPPPIETPGTPTSVPAAAGQVPAAPGTPSAASLLLPSSIMPAS----------RPPPGTWQSPPGPAVARASPPSLAP :  257
TaPERK16  : ----VAVYSPPPPRIASPPPPPRHHFK----------PHYAPP------------------------RSPGRPHSNSTR-----S :  222
TaPERK17  : LPDATPP---PPEDVPSSPPPPNDAPAAAAPPPSPLTLSPPPPPQSASAP------------------STSAKSSSSSGTAV--G :  228
TaPERK18  : --SPT--SPTVPATPTAPSPPSTPGFGPPS--VPSAPTTTPPTPIGPN-------------------MPQDPSWQDS-------- :  232
TaPERK19  : ------SSALNTATVAGIAVGGLIALLLASLLCFCMLKKKRRHHPHHPPP------------------PPPPHHLHYYGHPPPPP :  170
TaPERK20  : ----AGTSPPPLETPGTPTSVPAAAGQVPAAPGTPSSSSLLPPSGIMPAS----------GRPPGSWQSPPGPAAARASPPSLAP :  257
TaPERK21  : ----VAAYSPPPPRIASPPPPPRHHTK----------PHYAPP------------------------RSPGRPHSNSTR-----S :  224
TaPERK22  : PPDATPP---PPEDVPYSPPAPNDAPAEQAPPPSALTWSPPPPPLSTDVP------------------ATLAKSSSSSRTTVGLG :  311
TaPERK23  : ------------------------------------------------------------------------SAHYS-------- :    5
TaPERK24  : ------SSALNTATVAGIAVGGLIALLLASLLCFCMLKKKRRHHPHHPPP------------------PPPPHHLHYYGHPPPPP :  171
TaPERK25  : ----VAVYSPPPPRVASPPPP-RHHTK----------PHYAPP------------------------RSPGRSHLNSTR-----S :  223
TaPERK26  : ------------------------------------------------------------------------------------- :    -
TaPERK27  : -------SSESGGGSKGGGGSSGRGKGGSKQDDSPPVEAVVVG-----------------------------------------V :  162
TaPERK28  : QRNTPCFLDSPKSSSFAVDSG--KSIVGPDNSVYEPDRASLGAASLYVTGAPTWGVSNVGKFMEANNGSYIIHSPGQFLNTLEPE :  347
TaPERK29  : -------SSESGGGSKGGGGS--KGKGGSKQDGSPPVEAVVVG-----------------------------------------V :  158
TaPERK30  : --------RGSGDSSRSSQTSAHRVS-------PNTTAEIIFA-----------------------------------------A :  100
TaPERK31  : --------RGSDESSRSSSQASHRVS-------SNTTAEIIFA-----------------------------------------A :  104
TaPERK32  : QRNTPCFLDSPKSSSFAVDSG--KSIVGPDNSVYQPDRASLGAASLYVTGAPTWGVSNVGKFMEANNGSYIIHSPGQFLNTLDPE :  490
TaPERK33  : --VTPPTADRSVTAPAPPSTGGGMSSGATAGVAVVAVIAFLCFAGVFVCLTKRRKRKYSDQYYPGFAAPPYTPQHMSGEAPFLRV :  306
TaPERK34  : --VTPPTADRSVTAPAPPSTGGGMSSGATAGVAVVAVIAFLCFAGVFVCLTKRRKRKYSDQYYPGFAAPPYTPQHMSGEAPFLRV :  315
TaPERK35  : QRNTPCFLDSPKSSSFAVDSG--KSIVGPDNSVYQPDRASLGAASLYVTGAPTWGVSNVGKFMDANNGSYIIHSPGQFLNTLDPE :  487
TaPERK36  : VNSHPGAP-PGLVSIAPPGTVGVMSAQLPGAQG-GATGAGGMQAAGSSAS-----------------TSSTNGAGGSAASASAQQ :  286
TaPERK37  : --VTPPTADRSVTAPAPPSTGG-MSSGATAGVAVVAVIAFLCFAGVFVCLTKRRKRKYSDQYYPGFAAPPYTPQHMSGEAPFLRV :  314
AtPERK1   : ------SDGLSTGVVVGIAIGGVA-ILVILTLICLLCKKKRRRRHDDEAA------------------YYVP-----------PP :  181
AtPERK2   : ------SKELSKGAMVGIAIGGGFVLLVALALIFFLCKKKRRR--DNEA------------------------------------ :  260
AtPERK3   : ------PSRLSTGAVVGISIGGG---VFVLTLIFFLCKKKRPR--DDKA------------------------------------ :  155
AtPERK4   : ------PPGDTGGSRSDNPPSSGGSSGGGGGGRSNTNTAIIVG-----------------------------------------V :  156
AtPERK5   : -RTSGDGGSPSPPRSISPPQNSGDSDSSSGNHP-QANIGLIIG-----------------------------------------V :  193
AtPERK6   : -RNS-DRNSPSPPRALAPPRSSGGGSNSSGNN--EPNTAAIVG-----------------------------------------I :  191
AtPERK7   : -KTS-DHSSHSQPRSLAPP-TSNSGSNSSSND--GLNIGAVIG-----------------------------------------L :  179
AtPERK8   : ---TTTTSPPPPPATSASPPSSNP----------TDPSTLAPPPT----------PLPVVPREKPIAKPTGPASNNGN---NTLP :  224
AtPERK9   : -PSPPSPP-SDRPSQ-SPPPPPEDTKPQP-----PRRSPNSPPPTFSSP--------PRSPPEILVPGSNNPSQNNPTLRPPLDA :  267
AtPERK10  : TPSPPSPSDSKRPVHPSPPSPPEETLPPPKPSPDPLPSNSSSPPTLLPPS---SVVSPPSPPRKSVSGPDNPSPNNPTP-----V :  316
AtPERK11  : ---SPGNPLQPLDSPLGGESNRVPSSSSSPSPPSLSGSNNHSGGSNRH-------------------NANSNGDGGTSQQSNESN :  257
AtPERK12  : ---PPANPNAPP-SPFPTVPPKTPSSGPVVS-PSLTSPSKGTPTPNQG-------------------NGDGGGGGGG-------- :  241
AtPERK13  : ---PPAPPNAPPRNSSHALPPKSTAAG-----GPLTSPSRGVPSSGNS-------------------VPPPANSGGG-------- :  230
AtPERK14  : PAAVTLPFFGPAGQLPDGTVAPPIGPVIEPKTSPAESISPGTP--QPLVP------------------KSLPVTTSYHRSSAGFL :  282
AtPERK15  : ------RNVALTGLITGVVLGATFVLLGVCIFVCFYKRKKRKLK----------------------------------------- :   93
OsPERK1   : ------SSGLTTPVVVGIVVGGLVVLLLVTILLVCLFKKKKRHHHHHPPP------------------PPPPHLLHYYGHPPPPP :  130
OsPERK2   : ------SSGLSSTAVVWIIVAAGVVGAVLALCVLTLWIRRCRRQ----------------------------------------- :  134
OsPERK3   : ------PSFLNSVAVK---VAAGVVGLVLGLCVLALWIRRWRR------------------------------------------ :  120
OsPERK4   : --------SGSEDVARSALASARRGG-------YNAMVEIVFA-----------------------------------------A :   97
OsPERK5   : -------------------------------------------------------------------MSP--TSPLS-------- :    8
OsPERK6   : PQSPPSAANQSVVFIPVPVASNSPPGMLPPPQVIDATPSGAISSTNFPGG-----------------RNSTAGSSNTSLSQQQHT :  285
OsPERK7   : ----------SHASRRLSTSITFAGG-------LDVTSELVIA-----------------------------------------A :   34
OsPERK8   : ---------KSGGSS--------RGESGRYRGDVTAGVFVGLF-----------------------------------------V :  107
GmPERK1   : APSTPPSRTSPPPTPSSQPAPPSNSTPRSSPPSPPSITNLAPS-----------------PPSRILSSPPPPPAQNGTE----NS :  286
GmPERK2   : ------KSLDSPPSSRNSGSGSDSGSRDSNGGGGDDSSKAIVG-----------------------------------------A :  159
GmPERK3   : ------SGKDSSNKSLSNEKSLGSNN--------DSSNKVAMG-----------------------------------------A :  123
GmPERK4   : EPSTPPSRTSTPPTPSSQPAPTSNSTPRSSPPSPPSTTNLAPP-----------------PPSRVLSSPLPSPAQNGTK----NP :  301
GmPERK5   : -------HRLSPPKSSSSG--SSDGLKDSESH--SLSMPVIIG-----------------------------------------I :  156
GmPERK6   : -----------SGSLP--------------------------------VA------------------VNVQKPSIFHVSSG--L :  183
GmPERK7   : -----------GGSLP--------------------------------VA------------------VDVQKPSTFHVSSG--L :  202
GmPERK8   : S---SSSSSISTGVVVGIAVGAVAVLLVLSILCICCRKKKRRRDEEYYAP------------------PPQ-------------P :  199
GmPERK9   : ---SVSESPPKPPSSDVHPPSTLP----SDTSGSSPPATLPDPPTNTTAAQGPTVSLPSLPTEKPTARPTNDGTNSMSS--NNTP :  267
GmPERK10  : ----SLPRNAPSNDSTPRTSDK-PRSGHHGKHGGATSSHTNSTGSSPN-------------------QTSSDDNSEK-------- :  241
GmPERK11  : SPPSSSSSSISTGVVVGIAVGAVAVLLVLSILCICCRKKKRRRDEEYHAP------------------PPQQQ-----------P :  192
GmPERK12  : ------KSLDSPPSSRNSGSGSGSSNGGGNDSGGDGSSKAIVG-----------------------------------------A :  166
GmPERK13  : -----------PPTTRKNPFDSHHSTP-------SHTSGLAPP-----------------PPS----------AQN--------- :  115
GmPERK14  : ------SGKDSPNKSLN-AKSSGSNN--------DSGDKAAMG-----------------------------------------A :  112
GmPERK15  : ---PSSRRNAPSNDSTQGTSEK-SRSGHH-KHG--VASHTNSTGSSPN-------------------QTSSGDNSGK-------- :  149
GmPERK16  : ---SVSETPPKPPSSDVPPPSTLPSTPPSDPSGSSPPASLPDPPTNKTVVGGPKVSLPSLPTEKPTARPTNDGTNSMSS--NNTP :  298
```

```
                  520         *       540         *       560         *       580         *
TaPERK1  : PPPPQYKEDHYGATYQNWQNNAPPPPPDHVVKMHPSPPPAYANR--------PPQA----------------------------- :  229
TaPERK2  : ------AEDHYGATYQNWQNNAPPPPPDHVVKMHPSPPPAYANR--------PPQA----------------------------- :   47
TaPERK3  : PSVPSGGGSANAAMSSS-------------------------------------------------------------------- :  272
TaPERK4  : PPPPQYKEDHYGATYQNWQNNAPPPP-DHVVKMHPSPPPAYANR--------PPQA----------------------------- :  224
TaPERK5  : LFRNARMSPSSLRYYGIGLENGNYTVTLQFAEFAFPDAQSWKSRGRRVFDIYAQGERKEQNFDIRKVAGGKSYTAVRKQYTVPVT :  570
TaPERK6  : LFRNARMSPSSLRYFGIGLENGNYTVTLQFAEFDFPDGQSWKSTGRRVFDIYVQGVRKEQNFDIRKTAGGKSYTAVRKQYIVPVT :  570
TaPERK7  : LFQTARMSPSSLRYYGIGLENGNYTVTLQFAEFDSPDPQAWKSRARRVFDIYLQGERREKNFDIRKAAGGKSFVVVKKQYVVPVV :  593
TaPERK8  : LFRNARMSPSSLRYYGIGLENGNYTVTLQFAEFAFPDAQSWKSRGRRVFDIYVQGERKEQNFDIRKVAGGKSYTAVRKQYTVPVT :  571
TaPERK9  : LFRNARMSPSSLRYFGIGLENGNYTVTLQFAEFDFPDGQSWKSTGRRVFDIYVQGVRKEQNFDIRKTAGGKSYTAVRKQYIVPVT :  572
TaPERK10 : LFRNARMSPSSLRYYGIGLENGNYTVTLQFAEFAFPDAQSWKSRGRRVFDIYVQGERKEQNFDIRKVAGGKSYTAVRKQYTVPVT :  571
TaPERK11 : LFRNARMSPSSLRYFGIGLENGNYTVTLQFAEFDFPDGQSWKSTGRRVFDIYVQGVRKEQNFDIRKTAGGKSYTAVRKQYIVPVT :  481
TaPERK12 : LFQTARMSPSSLRYYGIGLENGNYTVTLQFAEFDSPDPQAWKSRARRVFDIYLQGERREQNFDIRKAAGGKSFVVVKKQYVVPVV :  592
TaPERK13 : RPSPSSGGLSNGAKAGIGIVVAILVLSLIGAGCWYKKKRRRMTGYHAGFVMPSPSPS---------------------------- :  290
TaPERK14 : PPPP-FKGDQYG-----WQHNAPPPPPDHVVKMN-SHPST--------------------------------------------- :  199
TaPERK15 : PARALGENLPIGVIVGVAVAGFLMALASLFIVACLSDRGKKRRPPMRKRG----------------------------------- :  307
TaPERK16 : NGSGKNIEISRETATTIVAIAGLAMLSFIGATIWLVKKKRRRAEPPSALPTQQQPAPPP-------------------------- :  281
TaPERK17 : VGVAVGTVVLLGLAAALIFCFVSRRRRLRRPE--TRADFFYDP------------RQP--------------------------- :  272
TaPERK18 : GPSPSSRGLNDGAKAGIGVVVVILVLSLLGAGCWYKKKRRRMTGYHAGFVMPSPSPS---------------------------- :  289
TaPERK19 : PPPP-FKGDQYG-----WQHNAPPPPPDHVVKMN-SHPSS--------------------------------------------- :  203
TaPERK20 : PARALGENLPIGVIVGVAVAGFLMALASLFMVACLSDWGKKRRPPMRKRR----------------------------------- :  307
TaPERK21 : NGSGKNIEISRETATTIVAIAGLAMLSFIGATIWLVKKKRRRAEPPSALPIQQQPAPPP-------------------------- :  283
TaPERK22 : VGVAVGAVVLLGLAAVIFFFLARRRRRPRVPPSGTPADFFYDP------------RQP--------------------------- :  357
TaPERK23 : KPNFCSG------------------------------------------------------------------------------ :   12
TaPERK24 : PPPP-FKGDQYG-----WQHNAPPPPPDHVVKMN-SHPST--------------------------------------------- :  204
TaPERK25 : NGSGKNIEISRETATTIVAIAGLAMLSFIGATIWLVKKKRRRAEPPSALPTQQQPAPPP-------------------------- :  282
TaPERK26 : ------------------------------------------------------------------------------------- :    -
TaPERK27 : VIGVLVFALLLCIAACVCCARRKKKK--PPMAMPFYTDQH--GNVYYANN-MPSPWQ---------------------------- :  214
TaPERK28 : LFQNARMSPSSLRYYGIGLENGNYTVTLLFAEFDFPDTQSWKSRGRRVFDIYVQGERKEQNFDIRKAAGGKSFTAVRKQYTVPVT :  432
TaPERK29 : VIGVLVFALLLCIAACVCCARRRKKKR-PPMAMPFYTDQH--GNVYYANN-MPSPWQ---------------------------- :  211
TaPERK30 : AGAAMLLALLIAACVCCS----RRTAPRRRRK--PHNP-MHFYADTSGNK----------------------------------- :  143
TaPERK31 : AGAAMLLALLIAACVCCS----RRTAPRRRRK--PHNP-MHFYADTSGNKGKTATLSRSPSIALACPVVLFLLLLICVRVVNHHC :  182
TaPERK32 : LFQNARMSPSSLRYYGIGLENGNYTVTLLFAEFDFPDTQSWKSRGRRVFDIYVQGERKEQNFDIRKAAGGKSFTAVRKQYTVPVT :  575
TaPERK33 : PSAPGSGNFSQPGMSAMMSQSYGNQQQQQQQQYQYQQQPP--------------------------------------------- :  346
TaPERK34 : PSAPGSGNFSQPGMSAMMSQSYGNQQQQQQ--YQYQQQPP--------------------------------------------- :  353
TaPERK35 : LFQNARMSPSSLRYFGIGLENGNYTVTLLFAEFDFPDTQSWKSRGRRVFDIYVQGERKEQNFDIRKAAGGKSFTAVRKQYTVPVT :  572
TaPERK36 : LHSSQVN----GQVVAAIAGASVAGLLFIIVAIFFIVTRRRRKMMGGMVYHSDGSRSYY-------------------------- :  341
TaPERK37 : PSAPGSGNFSQPGMSAMMSQSYGNQQQQQQ--YQYQQQPP--------------------------------------------- :  352
AtPERK1  : PPSGPKAGGPYGGQQQYWQQQNASRPSDNHVVTSLPPPKPPSP------------------------------------------ :  224
AtPERK2  : -PPAPIDGVPYGGQQ----QQNASRRSDHVVMSVPPPKSPSSA------------------------------------------ :  298
AtPERK3  : -LPAPIG------------------------------------------------------------------------------ :  161
AtPERK4  : LVGAG-LLMIVLIIVCLRRK-KKRKDSFYPEPMKGNQYQYYGNNNNNNASQNYPNWHLN-------------------------- :  213
AtPERK5  : LVGAG-LLLLLAVCICICCNRKKKKKSPQVNHMHYYNNNP-----YGGAPSGNGGY----------------------------- :  243
AtPERK6  : VAGAG-LLFLVMILFCVCCCRKKKKK----HQMPYYAGNG-----YATGK--GDQY----------------------------- :  235
AtPERK7  : VAAAG-ILFIVMILLCVCCFRKKKKKS-KLDQMPYYGSNA-----YPAGKTGGDQYYNQNAAT---------------------- :  235
AtPERK8  : SSSPGKSEVGTGGIVAIGVIVGLVFLSLFVMGVWFTRKRKRKDPGTFVGYTMPPSAY---------------------------- :  281
AtPERK9  : PNSTNNSGIGTGAVVGISVAVALVVFTLFGIFVWCLRKREKRLSAVSGGDVTPSPM----------------------------- :  323
AtPERK10 : TDNSSSSGISIAAVVGVSIGVALVLLTLIGVVVCCLKKRKKRLSTIGGGYVMPTPME---------------------------- :  373
AtPERK11 : YTEKTVIGIGIAGVLVILFIAGVFFVRRKQKKGSSSPRSNQYLPPANVSVNT--EGFIH-------------------------- :  314
AtPERK12 : YQGKTMVGMAVAGFAIMALIGVVFLVRRKKKRNIDSYNHSQYLPHPNFSVKS--DGFL--------------------------- :  297
AtPERK13 : YQGKTMAGFAIAGFAVIALMAVVFLVRRKKKRNIDAYSDSQYLPPSNFSIKS--DGFL--------------------------- :  286
AtPERK14 : FGGVIVGALLLILLGLLFVFYRATR--NRNNNSSSAHHQSKTP------SKVQHHRGG--------------------------- :  332
AtPERK15 : ------------------------------------------------------------------------------------- :    -
OsPERK1  : PPPPPFKGDHYGGVYQNWQQNGPPPPPDHVLKKVPSHPSP--------------------------------------------- :  170
OsPERK2  : -RRRRRQAQPFPLPPP-IYNPNPYYKGDLPPQPFVAQQPPSDHY----------------------------------------- :  176
OsPERK3  : -RRRRQQAQPLPLPPPMLYNPNPYYKGDQPPLPFVFMQQ---------------------------------------------- :  158
OsPERK4  : VGAAALLVLLVAACLCCS----RKTAPRRKRKKKPHNPVTHFDADTSGSK----------------------------------- :  143
OsPERK5  : SHHPSSGSGASANVGSS-------------------------------------------------------------------- :   25
OsPERK6  : TVSSTAQASSSGHIAAAIAGAAVTGLLCAIVAIYLIVSSRRKKKMDGLVYHYDGN-NYF-------------------------- :  343
OsPERK7  : VAAALLIVLLFAACACCSWCCRRRSATQRWQN--HHAAAFGYQGNTTAYY----------------------------------- :   82
OsPERK8  : VAMVAVLAGVVAIVVCCCIKITKKKKRPPPPNMPFFTDEK--GNVYYATGGLPPMWQ---------------------------- :  162
GmPERK1  : SPDGGGDGIGIGGVVAISVVAGFLLLGFIGVLIWCMRRKKRKVL-VSGDYVMPSTL----------------------------- :  341
GmPERK2  : VLGIG-SVLLILVIVCVVCSRKKKKNRMYYYAGEQSMGKGNNNNYYNSGQH--PNYYGG-------------------------- :  215
GmPERK3  : GIGVG-FILLVLVIVCFIWSCRKKKRKQKYYY--PNPSHGKASNYYNSGQHPLPSWQAGN------------------------- :  180
GmPERK4  : SPDGGGDGIGTGGVVAISVVAGFLLLGFIGVLIWCMRRQKRKLP-VSGGYVMPSTL----------------------------- :  356
GmPERK5  : AAGAG-ILLLLLLVVVCACSRRKKRSQ---PSVSYYANQPSDDGYYKRPRGGQNQYG---------------------------- :  209
GmPERK6  : IVGFVIGAIVFVFVFTVGSVLRWNRKKNKHKNLSTPHW-----------SHVIRVPS---------------------------- :  229
GmPERK7  : IFGLVIAAIVFVFVFTVGSVLRWNRKKNKHNNLSTPHYKESSPSLGSKGSHVIRVSP---------------------------- :  259
GmPERK8  : PRGP--KDDAYGGPPRQWQHN-VPPPQDHVVSMMPPKPSPPPAP--------PAYAAQ--------------------------- :  246
GmPERK9  : SHSGGLS---TGGSVAIGIVVGFTVLS-LVMAVWFVQKKKKKGTGSRGGYAAA-SPF---------------------------- :  319
GmPERK10 : YIGYTLIG--IFAVALVALAVSLVFVFKKKKSRGDAHVTP-YMPPLNIHVKSGVNGHY--------------------------- :  296
GmPERK11 : PRGP--KDGAYGGPPGQWQHN-VPPPQDHVVSMMPPKPSPPPAP--------PAYAGQ--------------------------- :  239
GmPERK12 : VLGVG-SVLLILVIVCVVCNRKKKK---------KNTNTWNHNNYYNSGQH--PSYYGG-------------------------- :  213
GmPERK13 : --SGPGSSSGTSAVAIVGVLTGVLLLGFIGIAIWCLRRQKERVS-KSGAYDLP-------------------------------- :  165
GmPERK14 : GIGVG-FVLLVLVIVCFIWRCKKKKRKQKYYY--PDPSQGKASNYYNSGQHPLPSWQAG-------------------------- :  168
GmPERK15 : YVGYTLAG--IFAVALVAVAVALVFVFKKKKSRRDAYTTP-YMPPFKIHVKSGANGHY--------------------------- :  204
GmPERK16 : SHSGGLS---TGGSVAIGIVVGFIVLSLLVMAVWFAQKKKKKGTGSRGSYAAP-SPF---------------------------- :  351
```

```
             600        *        620        *        640        *        660        *        680
TaPERK1  : --------------------------------PPPPPPPMINNSGGSGSNYSGG-EI--------------------------LP : 255
TaPERK2  : --------------------------------PPPPPPPMINSSGGSVSNYSGG-EI--------------------------LP :  73
TaPERK3  : -------------------------------------ADPSVHTNYS-----AG-----------------------------SP : 286
TaPERK4  : --------------------------------PPPPPPPMINSSGGSGSNYSGG-EI--------------------------LP : 250
TaPERK5  : KNFLEIHLFWAGKGTCCIPDQGYYGPAISALSATPNFTPTVRSAVAKKNDSKTGVIAGVVVGIAVLGLVVLAGIFLWRRRQKRRK : 655
TaPERK6  : KNFLEIHLFWAGKGTCCIPTQGYYGPAISALSATPNFTPTVRNAAAKKNGSKTGVIVGVIVGLAVLGLVAFAAIFVWR--QKKRK : 653
TaPERK7  : KNFLEIHLFWAGKGTCCIPTQGYYGPAISALSATPNFIPTVHYSVDNKSSSKTGVIVGVVIGVAVCLLAALAGVFVWR--QKRKK : 676
TaPERK8  : KNFLEIHLFWAGKGTCCIPDQGYYGPAISALSATPNFTPTVRSAAAKK--SKTGVIAGVVVGVAVLGFVVLAGIFLWRRRQKRRK : 654
TaPERK9  : KNFLEIHLFWAGKGTCCIPTQGYYGPAISALSATPNFTPTVRNAAAKKNGSKTGVIVGVIVGLAVLGLVAFAAIFFWR--QKKRK : 655
TaPERK10 : KNFLEIHLFWAGKGTCCIPDQGYYGPAISALSATPNFTPTVRSAAAKKNGSKTGVIAGVVVGVAVLGLVVLAGIFLWRRRQKRRK : 656
TaPERK11 : KNFLEIHLFWAGKGTCCIPTQGYYGPAISALSATPNFTPTVRNAAAKKNGSKTGMIVGVIVGLAVLGLVAFAAIFVWR--QKKRK : 564
TaPERK12 : KNFLEIHLFWAGKGTCCIPTQGYYGPAISALSATPNFIPTVHYSVDKKSSSKTGVIVGVVIGVAVCLLAALAGVFVWR--QKRKK : 675
TaPERK13 : ----------ASPQVLLG-------------------HSEKTKTNYS-----AG-----------------------------SP : 312
TaPERK14 : ---------------------------------KQPPPPVNVNSSGSGSHFSGGGEN--------------------------RP : 225
TaPERK15 : -------------------------------------TMVVPARAPDSYPPSNG-----------------------------PA : 326
TaPERK16 : --PPPNYIPSSAGSSLAS------------------DGFYLRSPGYPFMRSSTG-----------------------------SH : 317
TaPERK17 : --------------------------------TTPLQQSHALSTPSSTPPLMHSWAQ--------------------------SS : 299
TaPERK18 : ----------ASPQVLLG------------------GHSEKTKANYS-----AG-----------------------------SP : 312
TaPERK19 : ---------------------------------KPPPPPVNVNSSGSGSHFSGGGEN--------------------------RP : 229
TaPERK20 : -------------------------------------TMVVLAPAPDSYPPSNG-----------------------------PV : 326
TaPERK21 : --PPPNYIPSSAGSSLAS------------------DGFYLRSPGYPFMRSSTG-----------------------------SH : 319
TaPERK22 : --------------------------------TTPLQQSHPLSTPSSTPPLMHSWAQ--------------------------SS : 384
TaPERK23 : -------------------------------------HSEKTKTNYS-----AG-----------------------------SP :  26
TaPERK24 : ---------------------------------KLPPPPVNVNSSGSGSHFSGGGEN--------------------------RP : 230
TaPERK25 : --PPPNYIPSSAGSSLAS------------------DGFYLRSPGYPFMRSSTG-----------------------------SH : 318
TaPERK26 : ---------------------------------------------------MHSWAQ--------------------------SS :   8
TaPERK27 : ------QSGGP---VDGHG------------------GVGWHPQYPLGQGPLSEEMI------------------MSGSQG-TGS : 253
TaPERK28 : KNFLDIHLFWAGKGTCCIPTQGYYGPAISALSATPNFTPTVRNAAVKKKGSKTGVIAGAIVGVVVLGLLAFVGIFVWR--QKRRK : 515
TaPERK29 : ------QSGGP---SDGHG------------------GVGWHLQYPPGQGPLSEEMT------------------MSGSQG-SGS : 250
TaPERK30 : ------AGSTYYTGGPQPQ----------------CQSGTGAGPSTSTSRPPGAAWPLPPG------------MNSGVYSSPRDQ : 194
TaPERK31 : HGVATTASSTYYTGGPQPQ----------------WQSGTGAGPSTSTFDPPGGPW-----------------HNSGVHSSPRDQ : 234
TaPERK32 : KNFLDIHLFWAGKGTCCIPTQGYYGPAISALSATPNFTPTVRNAVIKK-GSKTGVIAGAIVGVVVLGLLAFAGIFVWR--QKKRK : 657
TaPERK33 : -------------------------------------PPQFASANYSSTMGSQG-----------------------------PA : 365
TaPERK34 : -------------------------------------PPQFASANYSSTMGSQG-----------------------------PA : 372
TaPERK35 : KNFLDIHLFWAGKGTCCIPTQGYYGPAISALSATPNFTPTVRNAVVKK-GSKTGVIAGAIVGVVVLGLLAFAGIFVWR--QKKRK : 654
TaPERK36 : --MHSGQLTGSNHPSGAF----------------YGPPPPGASGGFSYGSSTPG----------------------------PTD : 380
TaPERK37 : -------------------------------------PPQFASANYSSTMGSQG-----------------------------PA : 371
AtPERK1  : --------------------------------PRKPPPPPPPPAFMSSSGGSDYSDL--------------------------PV : 251
AtPERK2  : --------------------------------PPRPPHFMSSGSSGDYDSNYSDQSV--------------------------LP : 325
AtPERK3  : ------------------------------------------------------------------------------------- :   -
AtPERK4  : -----SQG----QNQQSTG--------------GWGGG--PSPPPPPPRMPTSGED-------------------SSMYSGPSRP : 254
AtPERK5  : ---YKGTPQDHVVNMAGQ------------------GGGNWGPQ-----QPVSG-------------------PHSDASNLTGRT : 283
AtPERK6  : QQQQYNNQSDHVMNLSQQY-PGSNGNNNWMNSPPPPPPGSWQPSPPPPPPPVSGGMNGNSS-----------DFSSNYS-GPHGP : 307
AtPERK7  : QQQQHYNQNDHIVNLPPP--PGSMG-TNWVSSPPPPPPGNWQPMPSPP-APVSGGANVIQS----------GEMSSNFSSGPYAP : 306
AtPERK8  : --------SSPQGSDVV--------------------LFNSRSSAPPKMRSHSG-----------------------------SD : 309
AtPERK9  : --------SSTARSDSA--------------------FFRMQSSAP-VGASKRS-----------------------------G- : 349
AtPERK10 : --------SSSPRSDSA--------------------LLKTQSSAP-LVGNRSS-----------------------------NR : 400
AtPERK11 : --YRQKPGNGNSSAQNS--------------------SPDTNSLGNPKHGRG--------------------------------- : 344
AtPERK12 : --YGQDPGKGYSSGPNGSM----------YNNSQQQQSSMGNSYGTAGGGYPHH-----------------------------QM : 341
AtPERK13 : --YGQNPTKGYS-GPGG------------YNS--QQQSNSGNSFGSQRGGGGYT-----------------------------RS : 325
AtPERK14 : --------------------------------NAGTNQAHVITMPPPIHAKYISSGG--------------------------CD : 359
AtPERK15 : ---------------------------------KKKKEDIEASINRDSLDPKDDSNN--------------------------LQ : 119
OsPERK1  : ---------------------------------PPPPAPLNVHSGGSGSNYSGGDNS--------------------------QP : 196
OsPERK2  : -----------------------------------FIQHQHPTPPQTSG-TFSDAG----------------------------S : 197
OsPERK3  : -------------------------------------HHHHPTAPQTSGGTFSDAG----------------------------S : 178
OsPERK4  : ------GGGGRDTSGPKP--------------------------------PPPPPW--------------------------LAE : 164
OsPERK5  : -------------------------------------LDPSFKTNYS-----AG-----------------------------SP :  39
OsPERK6  : --VPSSQFGGSSR---------------------NHHPPPSAIMLNSGGASADGGGYY------------------------NSG : 381
OsPERK7  : ---------YHHTGGARPQ----------------WAATKTGAPSTPPNMMMHPTN------------------MTGPHVVVRPP : 124
OsPERK8  : ------QHGSSNYSIPPPP------------------PPGWHMSSSAGG--FSGEMG------------------MGYSSGPYGP : 203
GmPERK1  : --------ASSPESDSS--------------------FFKTHSSAP-LVQSGSG-----------------------------SD : 368
GmPERK2  : -----PHG-DHVVRMQQNG-------------MGPGGGG-WGAPPPPPPMMMSSAE-------------------FSSNYSTGPA : 261
GmPERK3  : ---SGPHGGDHVMRIQQQQ-MGMGMGMG---GMGPHSGG-WGAP--PPHMMNSGEM-------------------MSSNYSLG-M : 235
GmPERK4  : --------ASSPESDSS--------------------FFKTHSSAP-LVQSGSG-----------------------------SD : 383
GmPERK5  : ----YNNNGEHVLNIPPP------------------PGAGWGAAPQPP-QMISS------------------DMSNSSFSGSHGP : 253
GmPERK6  : --------------------------------NPKLPPRP-LTEGGGASCSFNSVS----------------------------VK : 254
GmPERK7  : --------------------------------NPTLPPPPPLTEGGGASGSFNSVS----------------------------VK : 285
GmPERK8  : --------------------------------PPPPPPPFIISSGGSGSNYSGG-EF--------------------------LP : 272
GmPERK9  : --------TSSHNSGTL--------------------FLRSQSPAN-FLGSGSG-----------------------------SD : 346
GmPERK10 : --YVQQP------IPSP---------------------PLANNYGNGNASMHHL-----------------------------G- : 322
GmPERK11 : --------------------------------PPPPPPPFISSSGGSGSNNSGG-EF--------------------------LP : 265
GmPERK12 : -----PHG-DHVVRMQQNG-------------MGPGGGGGWGAPPPPP-MMMNSAD-------------------FSSNYSTGPP : 259
GmPERK13 : -----------PESDMP--------------------LHKIRSSAP-LIERASG-----------------------------G- : 188
GmPERK14 : ---SGPHGGDHVMRVQQQQGMGMGMGMGMGMGMGPHSGG-WGAP--PHPMMSSGE--------------------MSSNYSLG-M : 226
GmPERK15 : --YVQQP------IPSP---------------------PLANNYGNGNMSMQHL-----------------------------G- : 230
GmPERK16 : --------TSSHNSGTL--------------------FLRPQSPAN-FLGSGSG-----------------------------SD : 378
```

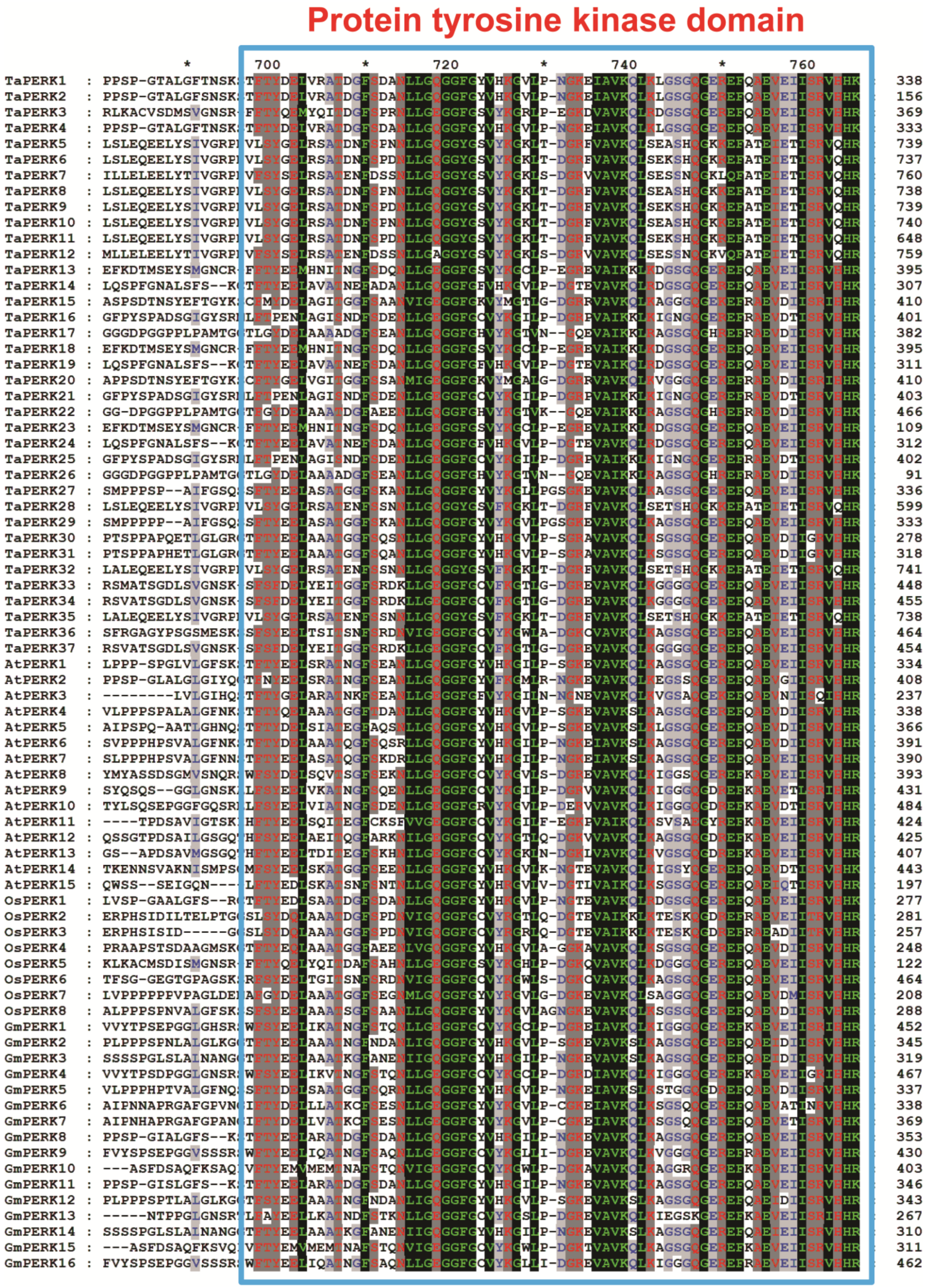
Protein tyrosine kinase domain
700
720
740
760

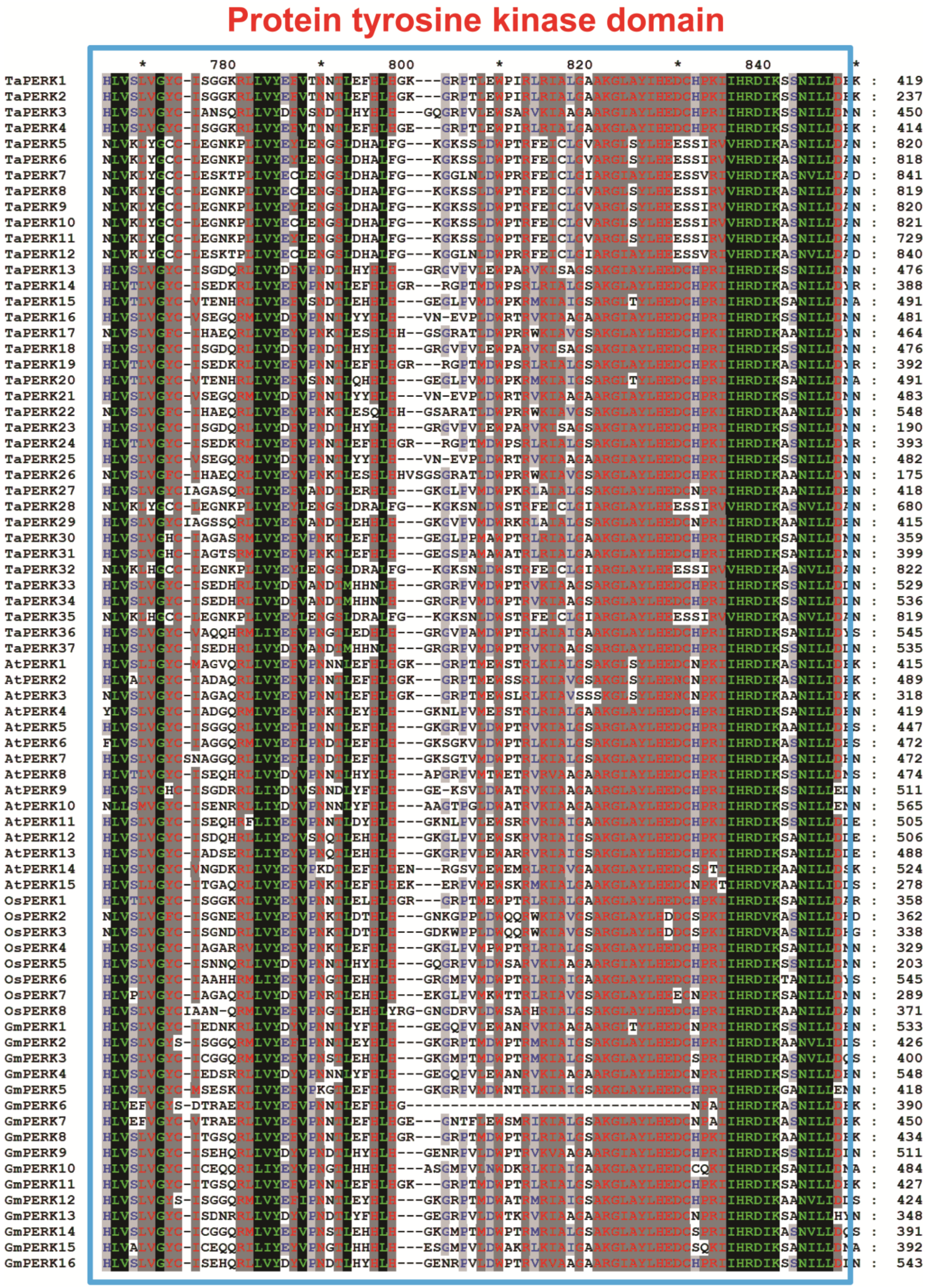
Protein tyrosine kinase domain
780
800
820
840
TaPERK1 419
TaPERK2 237
TaPERK3 450
TaPERK4 414
TaPERK5 820
TaPERK6 818
TaPERK7 841
TaPERK8 819
TaPERK9 820
TaPERK10 821
TaPERK11 729
TaPERK12 840
TaPERK13 476
TaPERK14 388
TaPERK15 491
TaPERK16 481
TaPERK17 464
TaPERK18 476
TaPERK19 392
TaPERK20 491
TaPERK21 483
TaPERK22 548
TaPERK23 190
TaPERK24 393
TaPERK25 482
TaPERK26 175
TaPERK27 418
TaPERK28 680
TaPERK29 415
TaPERK30 359
TaPERK31 399
TaPERK32 822
TaPERK33 529
TaPERK34 536
TaPERK35 819
TaPERK36 545
TaPERK37 535
AtPERK1 415
AtPERK2 489
AtPERK3 318
AtPERK4 419
AtPERK5 447
AtPERK6 472
AtPERK7 472
AtPERK8 474
AtPERK9 511
AtPERK10 565
AtPERK11 505
AtPERK12 506
AtPERK13 488
AtPERK14 524
AtPERK15 278
OsPERK1 358
OsPERK2 362
OsPERK3 338
OsPERK4 329
OsPERK5 203
OsPERK6 545
OsPERK7 289
OsPERK8 371
GmPERK1 533
GmPERK2 426
GmPERK3 400
GmPERK4 548
GmPERK5 418
GmPERK6 390
GmPERK7 450
GmPERK8 434
GmPERK9 511
GmPERK10 484
GmPERK11 427
GmPERK12 424
GmPERK13 348
GmPERK14 391
GmPERK15 392
GmPERK16 543

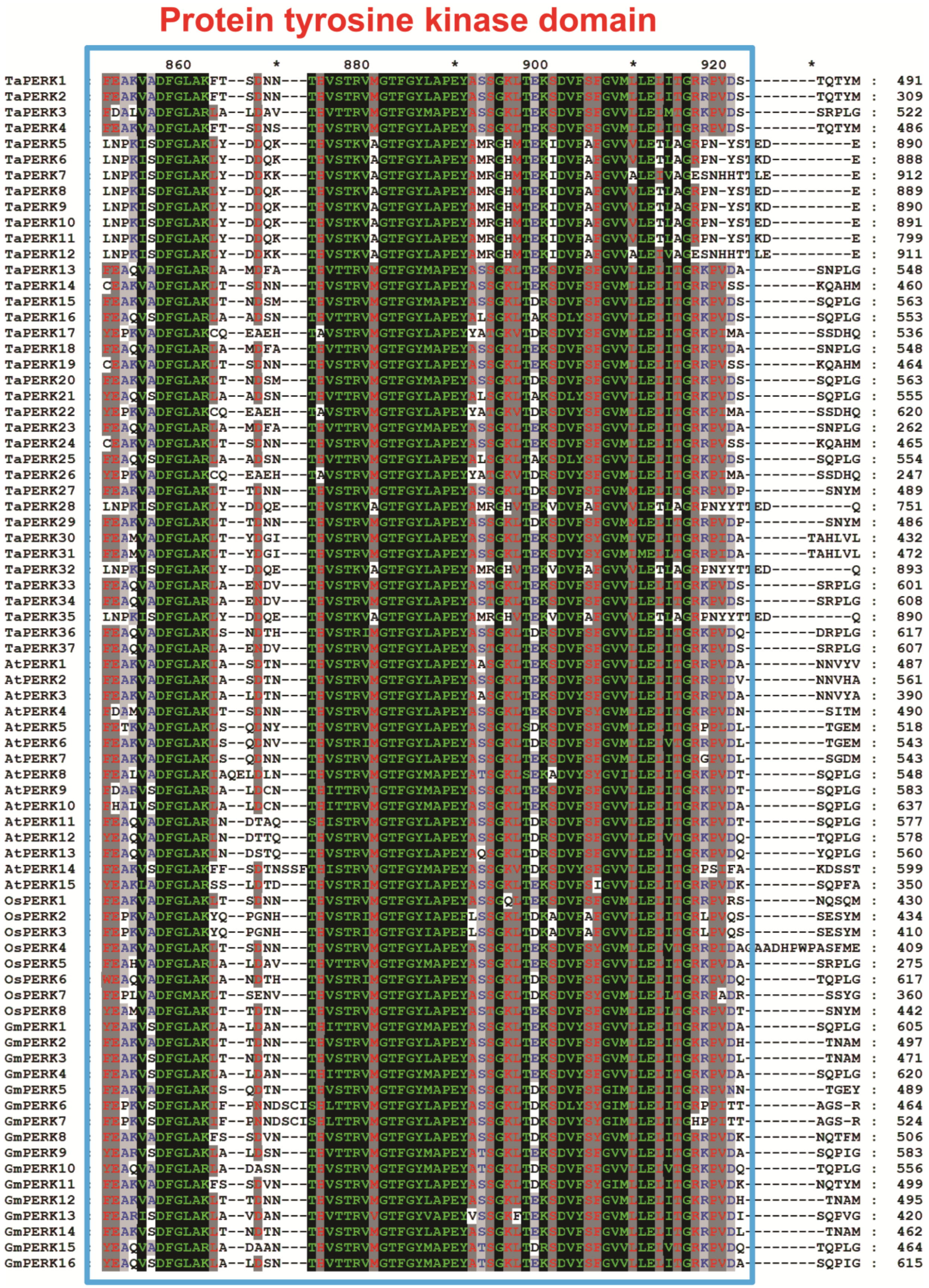
Protein tyrosine kinase domain
860
880
900
920

Protein tyrosine kinase domain

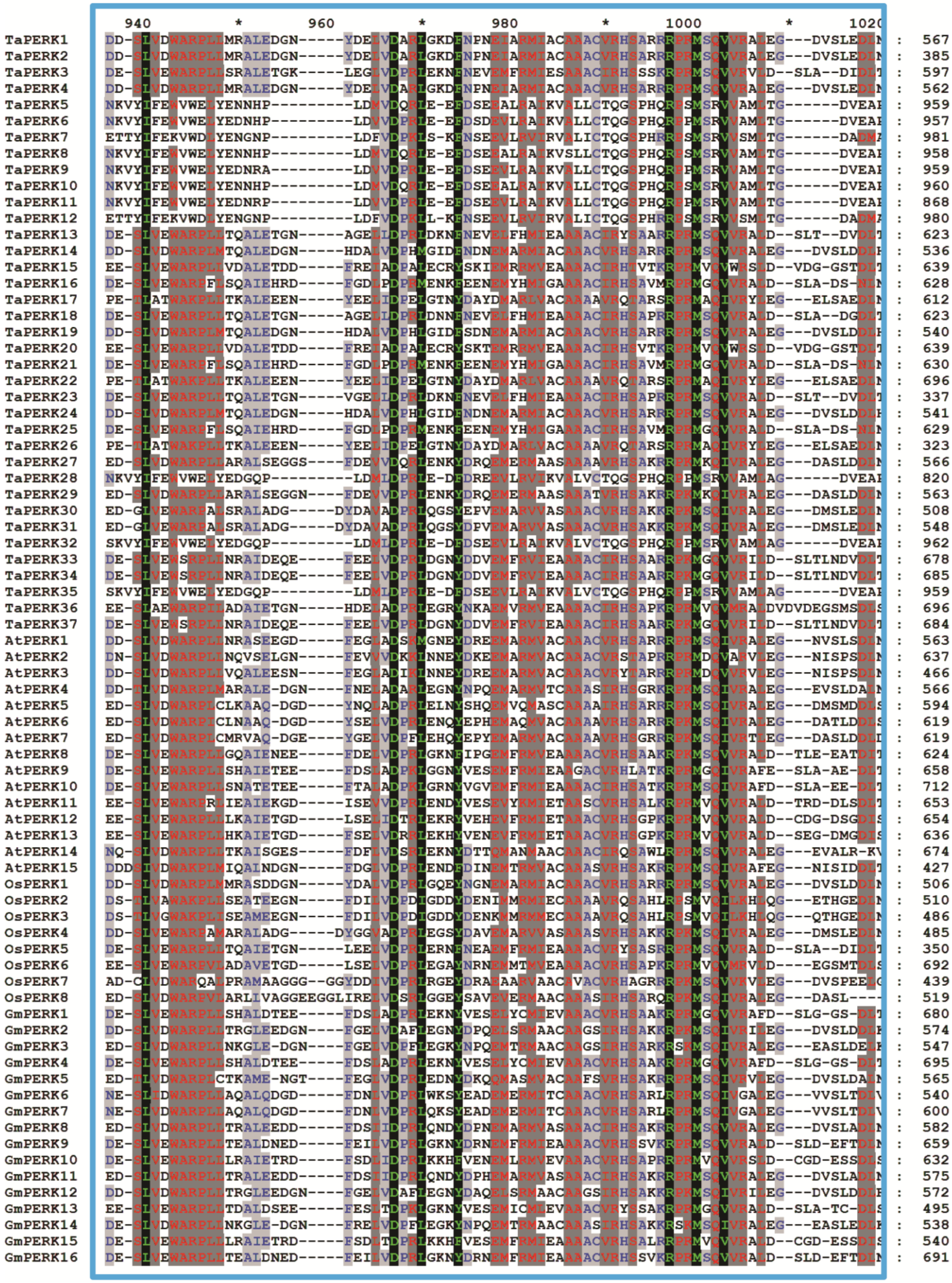

```
                         *      1040         *       1060         *       1080         *       1100
TaPERK1   : -EGVRP-----GHSRFFGSYS---SSDYDSGQYNEDMKKFKK--MAFTTNDY------------TSSQYSAPTS---EYGQIPS- :  625
TaPERK2   : -EGVRP-----GHSRFFGSYS---SSDYDSGQYNEDMKKFKK--MAFTTNDY------------TSSQYSAPTS---EYGQIPS- :  443
TaPERK3   : -NGVQP-----GQSELFN------------VANTADIRMFQQMVQGNQDD---------------SSAFSQYS----WISRSRAD :  645
TaPERK4   : -EGVRP-----GHSRFFGSYS---SSDYDSGQYNEDMKKFKK--MAFTTNDY------------TSSQYSAPTS---EYGQIPS- :  620
TaPERK5   : -DVVTK------------------------PSYITEWQITGG-NTTYMST-------------DVSGQPSSAPRPNSPSSRTSSP : 1005
TaPERK6   : -DVVTK------------------------PSYITEWQIKGG-NTTYMST-------------DVSGQSSSAPRPHSSTSQTSSP : 1003
TaPERK7   : GEEVIK------------------------PSYITEWQVKAGSSSSYTGTSSQAGLSSSSTTQQSSGGGHAAGGDATPATGNPSP : 1042
TaPERK8   : -DVVTK------------------------PSYITEWQITGG-NTTYMST-------------DVSGQPSSAPRPNSPSSRTSSP : 1004
TaPERK9   : -DVVTK------------------------PSYITEWQIKGG-NTTYMST-------------DVSGQSSSAPRPHSSTSQTSSP : 1005
TaPERK10  : -DVVTK------------------------PSYITEWQITGG-NTTYMST-------------DVSGQPSSAPRANSPSSRTSSP : 1006
TaPERK11  : -DVVTK------------------------PSYITEWQIKGG-NTSYMST-------------DVSGQSSSAPRPNSSTSQTSSP :  914
TaPERK12  : GEEVIK------------------------PSYITEWQVKAGSSSSYTGTSSQAGLSSSSTTQQSSGGGHAAGGDATPATGNPSP : 1041
TaPERK13  : -NGVQP-----GMSEMFN------------APNTAEIRLFQRMAFGSQDF---------------TTDFTQSSS---WNSSRQGG :  672
TaPERK14  : -EGVRP-----GHSRFMGSHA---SSEYDTSQYNEDLKKFRK--MALGTSSF------------QSSQLTPSSGEHEQEHQNPS- :  597
TaPERK15  : -NGVKL-----GHSTAYDS-----------GQYSADIELFRRMGFEADLDT-------------AEYGLSDGGLVPGGNSGQQLD :  694
TaPERK16  : -NGLQP-----GRSEVFL------------EPRTEEIRLFQLREFGSRE-C--------------SDELSQAS----WRSRRDL- :  675
TaPERK17  : -GGMAP-----GQSAMHRSGGG--NTDEVRRLRRMAFGPGTG--TAGGTISEY-----------ASSEMSAPTS---EYGLNPS- :  672
TaPERK18  : -NGVQP-----GMSEMFN------------APNTAEIRLFQRMAFGSQDF---------------TTDFTQSSS---WNSSRQGG :  672
TaPERK19  : -EGVRP-----GHSRFMGSHA---SSEYDTSQYNEDLKKFRK--MALGTSSF------------QSSQLTPSSD--EHERQDPS- :  599
TaPERK20  : -NGVKL-----GHSTAYDS-----------GQYSADIELFRRMGFEADLDT-------------AEYGLSDQDELPPGSGRQQR- :  693
TaPERK21  : -NGLQP-----GRSEVFL------------EPRTEEIRLFQLREFGSRE-C--------------SDELSQAS----WRSRRDL- :  677
TaPERK22  : -GGMAP-----GQSAMHRSGGG--NTDEVRRLRRMAFGPGTG--TAGGTISEY-----------ASSEMSAPTS---EYGLNPS- :  756
TaPERK23  : -NGVQP-----GMSEMFN------------APNTAEIRLFQRMAFGSQDF---------------TTDFTQSSS---WNSSRQGG :  386
TaPERK24  : -EGVRP-----GHSRFMGSHA---SSEYDTSQYNEDLKKFRK--MALGTSSF------------QSSQLTPSSG--EHEHQEPS- :  600
TaPERK25  : -NGLQP-----GRSEVFL------------EPRTEEIRLFQLREFGSRE-C--------------SDELSQAS----WRSRRDL- :  676
TaPERK26  : -GGMAP-----GQSAMHRSGGG--NTDEVRRLRRMAFGPGTG--TAGGTISEY-----------ASSEMSARTS---EYGLNPS- :  383
TaPERK27  : -EGMKP-----GQSMIYS--------SDESGSYAANINRLRQVAFESS-GEY-------------TNEYS---------GTGESG :  614
TaPERK28  : -DWVPK------------------------PSYITEWHIKGGGDTSYMSS-------------EVNGQSSSVP----------AP :  857
TaPERK29  : -EGMKP-----GQSMIYS--------SDESGSYAANINRLRQIAFESS-EEY-------------TNEYS---------GTGESG :  611
TaPERK30  : -DGVRP-----GQSRLFE---------EGAGSCSSDMNRTKVVVV--ASPEY-------------SGRFGRPSPV--SSDRPISD :  561
TaPERK31  : -DGVRP-----GQSRLFG---------EEAGSYSSDMNRAKEVAV--ASPEY-------------SGRSGRPSPV--SSDRPISD :  601
TaPERK32  : -DWVPK------------------------PSYITEWQIKGGGDTSYMSS-------------EVNGQSSSEP----------AP :  999
TaPERK33  : -NGVQP-----GKSQMFN------------AANTADIRQFQRMAFGSQDFS--------------SSEYTQSKA---SLSGRRDL :  728
TaPERK34  : -NGVQP-----GKSQMFN------------AANTADIRQFQRMAFGSQEFT--------------SSEYAQSKA---SLSGRRDL :  735
TaPERK35  : -DWVPK------------------------PSYITEWQIKGGGDTSYMSS-------------EVNGQSSSEP----------AP :  996
TaPERK36  : -NGVKV-----GQSQVFNN-----------SQQEAALEQLRRTPSPPRSSRGS-------------SSRPGSSS----RPGSTVP :  747
TaPERK37  : -NGVQP-----GKSQMFN------------AANTADIRQFQRMAFGSQDFS--------------SSEYTQSKA---SLSGRRDL :  734
AtPERK1   : -EGMRP-----GHSNVYSSYGG--STDYDTSQYNDDMIKFRK--MALGTQEYG-----------TTGEYSNPTS---DYGLYPS- :  623
AtPERK2   : -QGITP-----GHSNVYGSSGG--STDYDSSQDNEGMNKFRK--VGL-----E-----------TQDLYSNPIS---EYDLYPS- :  692
AtPERK3   : -QGITP-----GHSNVYSSXRG--STYYDTRTMXTYTRKFRI--KMG-----H-----------GXGEYSNPTS---DYRLYPSG :  522
AtPERK4   : -EGVKP-----GHSNVYGSL--GASSDYSQTSYNADMKKFRQIALS-------------------SQEFPVSDCE--GTSSNDSR :  622
AtPERK5   : -EGTRP-----GQSTYLSPG--SVSSEYDASSYTADMKKFKKLALENKEY-Q-------------SSEYGGTSEYGLNPSASSSE :  657
AtPERK6   : -EGGKA-----GQSSFLGRG--SSS-DYDSSTYSADMKKFRKVALDSHEYGA-------------SSEYGNTSEYGLDPSSSSSE :  682
AtPERK7   : -DGVKP-----KQSSSGGEG--SS--DYEMGTYGAEMRKFRKVTLESRDYGA-------------SSEYGATSEYGLDPSSSSSE :  681
AtPERK8   : -NGMRP-----GQSQVFDS-----------RQQSAQIRMFQRMAFGSQDYS--------------SDFFDRSQSHSSWGSRDQ-- :  676
AtPERK9   : -NGMRL-----GESEVFNS-----------AQQSAEIRLFRRMAFGSQNYS--------------TDFFSHSS----YNSRDANV :  708
AtPERK10  : -NGMRL-----GESEIINS-----------AQQSAEIRLFRRMAFGSQNYS--------------TDSLTRNS----YISKDENL :  762
AtPERK11  : -NGVKV-----GQSRVYDS-----------GQYSNEIRIFRRAS---EDSSDLG---------TNTGYYPSQ--------DYATS :  701
AtPERK12  : -NGIKI-----GQSTTYDS-----------GQYNEDIMKFRKMAFGGDNSVESG---------LYSGNYSAK--------SSSDF :  705
AtPERK13  : -NGNKV-----GQSSAYDS-----------GQYNNDTMKFRKMAFGFDDSSDSG---------MYSGDYSVQDSRKGSNGASSEF :  695
AtPERK14  : -EETGN-----S---VTYSSSE--NPNDITP----RYGTNKRRFDTG-----------------SSDGY---TS---EYGVNPS- :  720
AtPERK15  : -EGAAP-----GQSTIYSLDG---SSDYSSTQYKEDLKKFKK--MAFESKTF------------GSSECSGLTS---DNGQNPS- :  485
OsPERK1   : -EGVRP-----GHSRFLGSYN---SNEYDTGHYNEDLKKFRK--MAFGSGNL------------ESSQQTQPTE----FVPNRS- :  563
OsPERK2   : --------------SIFR------------ITYAEDTYSSIMESGESIGPRSRR---------APRSQRNTSSDYSSEQALTDKA :  560
OsPERK3   : --------------SIFR------------TTYAEDTYSSIMESG------------------LPRS---------------DLF :  512
OsPERK4   : -EGMRP-----GQSMVFGTAETGGSISEASGSYTFDMDRIIQEAT--AARLE-------------SGR---------RDDVSFSG :  540
OsPERK5   : -NGVQP-----GQSELFN------------VANTAEVRMFQRMVLGNHDD---------------SSDMSQYG----WSS-SRQ- :  396
OsPERK6   : -NGIKV-----GQSQVFTG-----------GSDAADIQQLRRIAFASEEFTGEF---------EQRTTNSNSES----RPMNRIP :  747
OsPERK7   : -DGARP-----GQSAMSSSS---GDSSSGSGSYTAQMERVRRTAASPASPEYY------------SSEYQGYGCP--SPASSAGD :  501
OsPERK8   : -------------SLDHH--------HDDDFSASSEISRHRQVAFDS--GDY-------------TDDYS---------TTSTST :  559
GmPERK1   : -NGMRL-----GESEVFD------------AQQSEEIRLFRRMAFGNQDYS--------------SDPHSCA------------- :  720
GmPERK2   : -DGIKP-----GQNVAYNSS--SSSDQYDTMQYNADMQKFRKAVFSN------------------SEEFG--------TSSGSSG :  625
GmPERK3   : -DGMKLKGSGNGNSSAPTSS--SGSSEYDTMQYNADMIKFRQAIMS-------------------SQEFN--------DSS---- :  598
GmPERK4   : -NGMRL-----GESQVFDS-----------AQQSEEIRLFRRMAFGSQNYS--------------TDFFSRAS----LNP----- :  740
GmPERK5   : HEGVKP-----GQSSMFSSA--SR--EYGAEAYGADMMRFRKLALDSGVG---------------SSEYGGTSEYGLNPSSSSSE :  626
GmPERK6   : -GDVTT-----G---LTTDTV---------------------------------------------------------------- :  552
GmPERK7   : -GDVTT-----G---LTTDTVY--NWSNILDYDAGHYQQDMRNFNLALSCHKY-----------SSSGYSETTS---AYGLHSS- :  659
GmPERK8   : -EGIRP-----GHSTMYSSHE---SSDYDTAQYKEDMKKFRK--MALGTQEYG-----------ASSEYSAATS---EYGLNPS- :  641
GmPERK9   : -NGMKP-----GQSSVFDS-----------AQQSAQIRMFRRMAFGSQD-S--------------SSFFNESQ--SSWRSRDHNS :  710
GmPERK10  : -NGVKY-----GHSTVYDS-----------GQYDKEIMLFRRLANGTFVDSDFE---------IYSREYSLSRDTSMTSRGSHQE :  691
GmPERK11  : -EGIRP-----GHSTMYSSHE---SSDYDTAQYKEDMKKFRK--MALGTQEYG-----------ASSEYSAATS---EYGLNPS- :  634
GmPERK12  : -DGIKP-----GQNVVYNSS--PSSNQYDTMQYNADMQKFRKAVFSN------------------SDEFG--------TSSGSSG :  623
GmPERK13  : -NGMRI-----GDS----------------ALQSAEIRLFRRMAFGIQDYN--------------SDFLSPRE------------ :  532
GmPERK14  : -DGMKLKGSGNGNSSAYPSS--YGSSEYDTMQYNADMIKFRQAIMS-------------------SQEFN--------DSS---- :  589
GmPERK15  : -NGMKY-----GHSTVYDS-----------GQYDKAIMLFRRMANGNFDDSDFD---------MVSREYSLSRDTSMTSRGSQQE :  599
GmPERK16  : -NGMKP-----GQSSVFDS-----------AQQSAQIRMFRRMAFGSQD-S--------------SGFFNESQ--SSWRSRDHDP :  742
```

```
                     *      1120         *      1140         *      1160
TaPERK1    : -ASSSEG--QQTQ-EIETGTMKKGGHSG--YSSGYSGPS----------------- :  658
TaPERK2    : -ASSSEG--QQTQ-EIETGTMKKGGHSG--YSSGYSGPS----------------- :  476
TaPERK3    : AEATSSNSRIL--------------------------------------------- :  656
TaPERK4    : -ASSSEG--QQTQ-EIETGTMKKGGHSG--YSSGYSGPS----------------- :  653
TaPERK5    : FLSSVIDEGR---------------------------------------------- : 1015
TaPERK6    : FLSSVIDEGR---------------------------------------------- : 1013
TaPERK7    : MFTSIIDEGR---------------------------------------------- : 1052
TaPERK8    : FLSSVMDEGR---------------------------------------------- : 1014
TaPERK9    : FLSSVIDEGR---------------------------------------------- : 1015
TaPERK10   : FLSSVIDEGR---------------------------------------------- : 1016
TaPERK11   : FLSSVIDEGR---------------------------------------------- :  924
TaPERK12   : IFTSIIDEGR---------------------------------------------- : 1051
TaPERK13   : -DADASGGPMHSQLQP---------------------------------------- :  687
TaPERK14   : -VPSSDGH-QQTQ-EVELGTSTKRDDGD--VESQASMR------------------ :  630
TaPERK15   : GEAHQINQLGSPAGRLNPPATLHRTP------------------------------ :  720
TaPERK16   : -------------------------------------------------------- :    -
TaPERK17   : -SEYTASSAADTEDMTDFPHRAGAGRGSAEGGSGEAGRGTTEGFSRRTTVRRTGRG :  727
TaPERK18   : GDVDASGGPRQSQP------------------------------------------ :  686
TaPERK19   : -VQSSDGH-RQTQ-EVELGTT-KRDDGD--VESQTSMR------------------ :  631
TaPERK20   : ----------TAAGR----------------------------------------- :  698
TaPERK21   : -------------------------------------------------------- :    -
TaPERK22   : -SEYTASSAADTEDMTDFPHRAGTGRGAAEGASGEAGRGTTEGFSRRTTGRRTSRG :  811
TaPERK23   : -DVDASGGPRHPQSQP---------------------------------------- :  401
TaPERK24   : -VPSSDGH-QQTQ-EVELGTR-KRDDGD--VESQASMR------------------ :  632
TaPERK25   : -------------------------------------------------------- :    -
TaPERK26   : -SEYTASSAADTEDMTDFPHRAGTGRGAAEGVSGEAGRGTTEGFSRRTTVRRTGRG :  438
TaPERK27   : ETTQRHH------------------------------------------------- :  621
TaPERK28   : FLGSVIDQGR---------------------------------------------- :  867
TaPERK29   : ETTQRHH------------------------------------------------- :  618
TaPERK30   : EMSPAERQQPHL-------------------------------------------- :  573
TaPERK31   : EMSPAERQPPHL-------------------------------------------- :  613
TaPERK32   : FLGSVIDQGR---------------------------------------------- : 1009
TaPERK33   : -------------------------------------------------------- :    -
TaPERK34   : -------------------------------------------------------- :    -
TaPERK35   : FLGSVIDQGR---------------------------------------------- : 1006
TaPERK36   : PPVL---------------------------------------------------- :  751
TaPERK37   : -------------------------------------------------------- :    -
AtPERK1    : -GSSSEGQ-ATREM--EMGKIKKTGQGY----SGPSL------------------- :  652
AtPERK2    : -WSSTDGQ-TTQGK--ATGNIKRPGQGY----G----------------------- :  717
AtPERK3    : GRSSSEGQ-ATRGNGFEMGKIKRTAQRY----GGDSL------------------- :  554
AtPERK4    : DMGTKSPTPPK--------------------------------------------- :  633
AtPERK5    : EMNRGSMKRNPQL------------------------------------------- :  670
AtPERK6    : EIRRGGANNNKTTPSRDH-------------------------------------- :  700
AtPERK7    : EMHIGGSTSKTTTTNRGI-------------------------------------- :  699
AtPERK8    : -SRFVP-------------------------------------------------- :  681
AtPERK9    : -------------------------------------------------------- :    -
AtPERK10   : -------------------------------------------------------- :    -
AtPERK11   : HEYESESRAFNTSHRNH--------------------------------------- :  718
AtPERK12   : SGNESETRPFNNRRF----------------------------------------- :  720
AtPERK13   : TRNETENRNFNNRRY----------------------------------------- :  710
AtPERK14   : -QSSSEHQQVNT-------------------------------------------- :  731
AtPERK15   : -GSSSITEGQRTTQEIEPEKNTKDT------------------------------- :  509
OsPERK1    : -VSMGDAR-QIPETEMEMGSLKKDGDSNGTRDSQAS-------------------- :  597
OsPERK2    : NRSPAKGR------------------------------------------------ :  568
OsPERK3    : TKQPNNYR------------------------------------------------ :  520
OsPERK4    : EMSAEWKQPPHRVSR----------------------------------------- :  555
OsPERK5    : -------------------------------------------------------- :    -
OsPERK6    : E------------------------------------------------------- :  748
OsPERK7    : NASSGEHDNQWRKAHR---------------------------------------- :  517
OsPERK8    : HSSRLPPKR----------------------------------------------- :  568
GmPERK1    : -------------------------------------------------------- :    -
GmPERK2    : EVSQKQQRL----------------------------------------------- :  634
GmPERK3    : ELSSKEMGYH---------------------------------------------- :  608
GmPERK4    : -------------------------------------------------------- :    -
GmPERK5    : QSSAEYARRTTGGGRMHTP------------------------------------- :  645
GmPERK6    : -------------------------------------------------------- :    -
GmPERK7    : -GSSSEAQQSFRDIV----------------------------------------- :  673
GmPERK8    : -GSSSEAQSRQTTREMEMRKMKNSQGFS-----GSS-------------------- :  671
GmPERK9    : NTMFSQNKTGPWNV------------------------------------------ :  724
GmPERK10   : LIRHSSSGSLNLELSTCIGPVLNENRD----------------------------- :  718
GmPERK11   : -GSSSEAQSRQTTREMEMRKMKNNQGFS-----GSS-------------------- :  664
GmPERK12   : EVSQKQQRL----------------------------------------------- :  632
GmPERK13   : -------------------------------------------------------- :    -
GmPERK14   : ELSSKEMSYH---------------------------------------------- :  599
GmPERK15   : LIQHSSSGEFDSRDINMHRSSIE--------------------------------- :  622
GmPERK16   : TTVFSQNKTGHGNV------------------------------------------ :  756
```

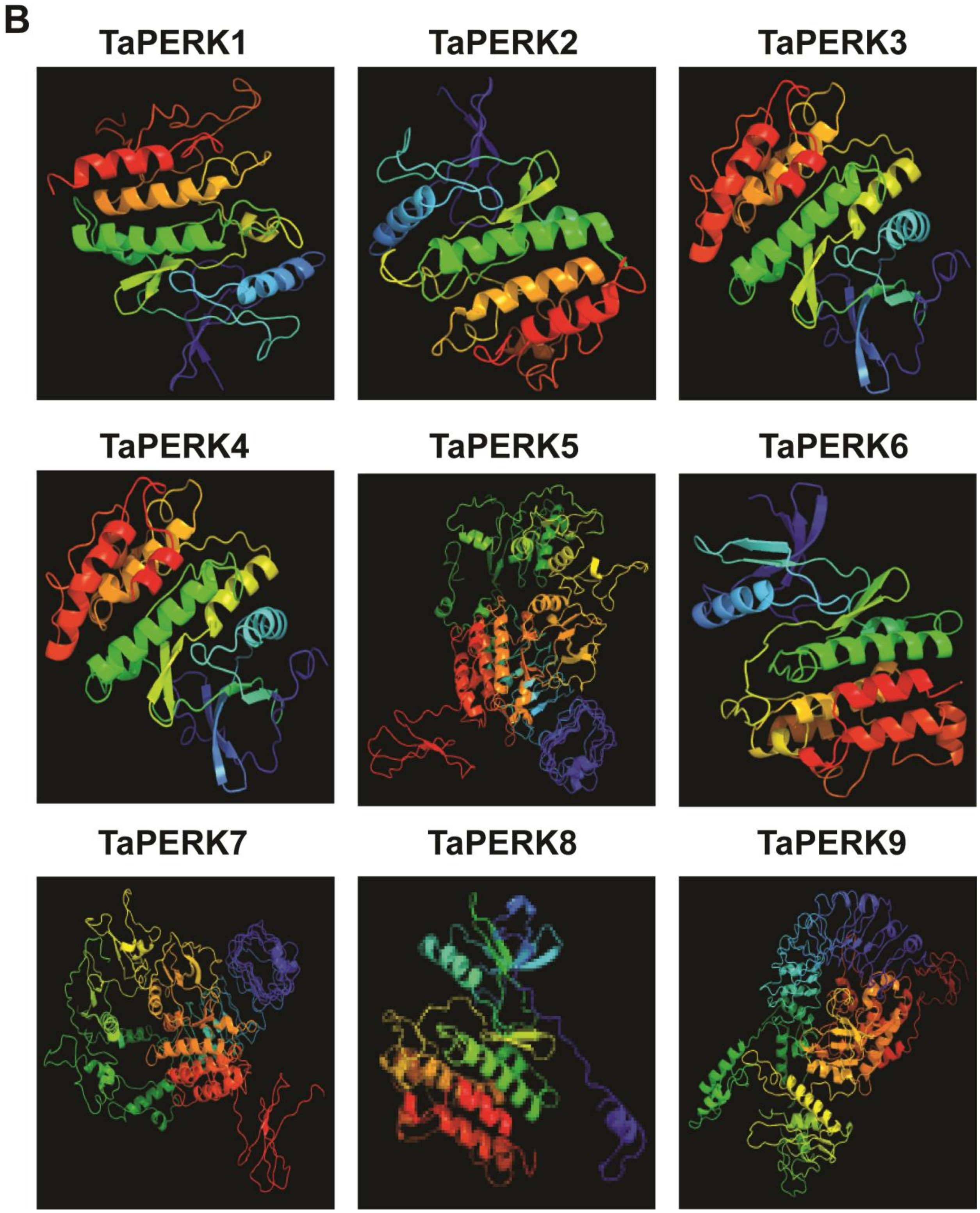
B
TaPERK1
TaPERK2
TaPERK3
TaPERK4
TaPERK5
TaPERK6
TaPERK7
TaPERK8
TaPERK9

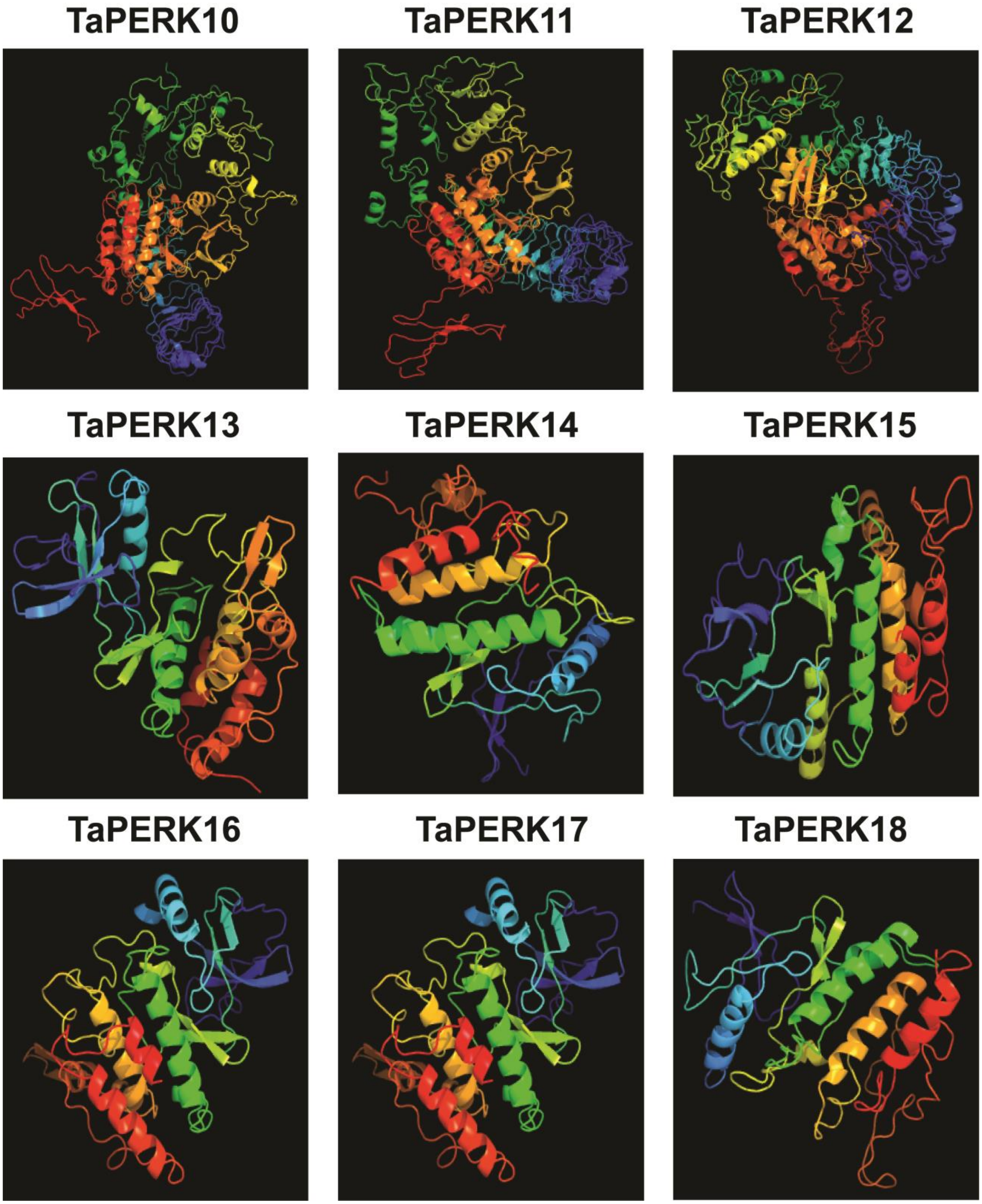
TaPERK10
TaPERK11
TaPERK12
TaPERK13
TaPERK14
TaPERK15
TaPERK16
TaPERK17
TaPERK18

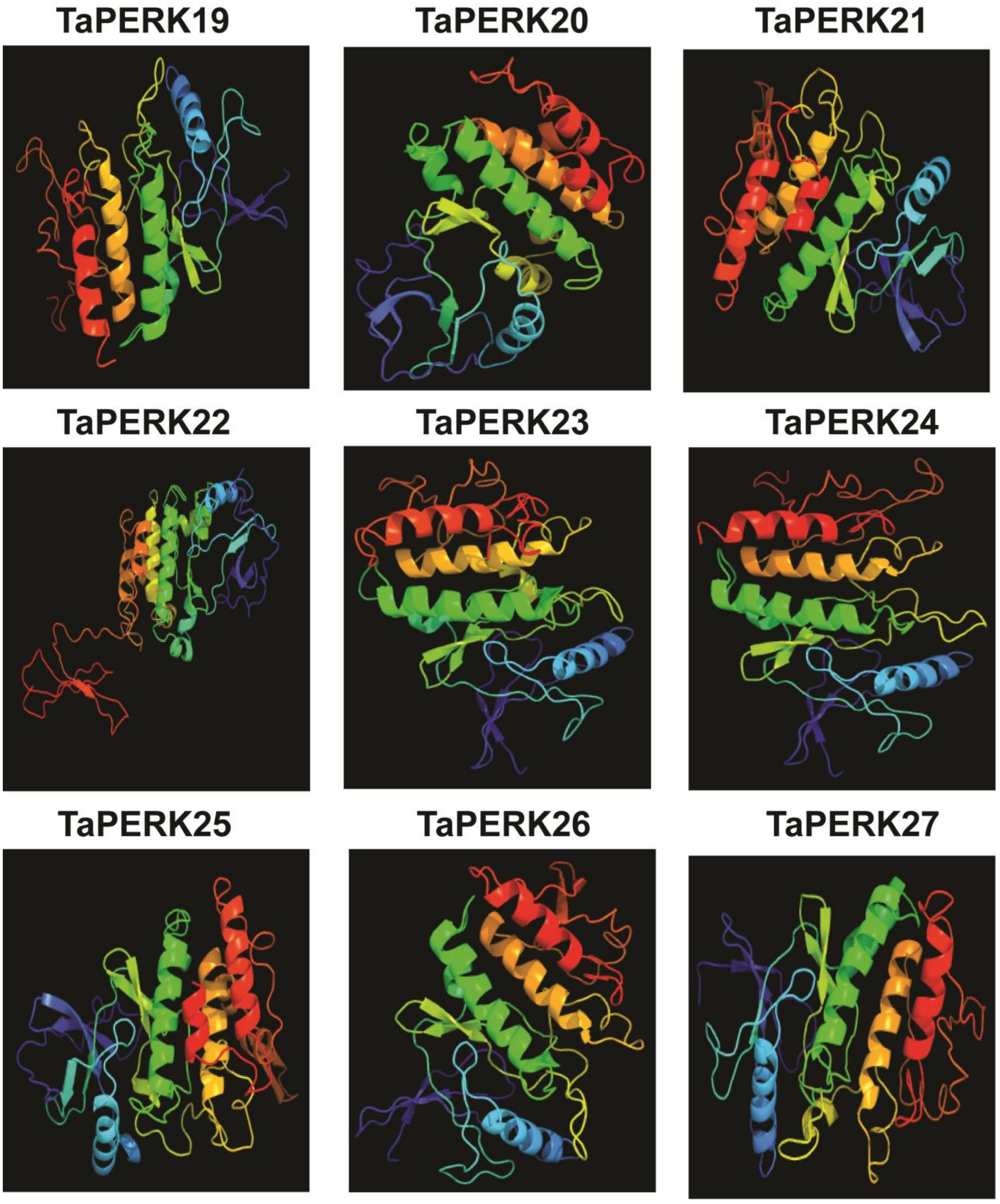
TaPERK19
TaPERK20
TaPERK21
TaPERK22
TaPERK23
TaPERK24
TaPERK25
TaPERK26
TaPERK27

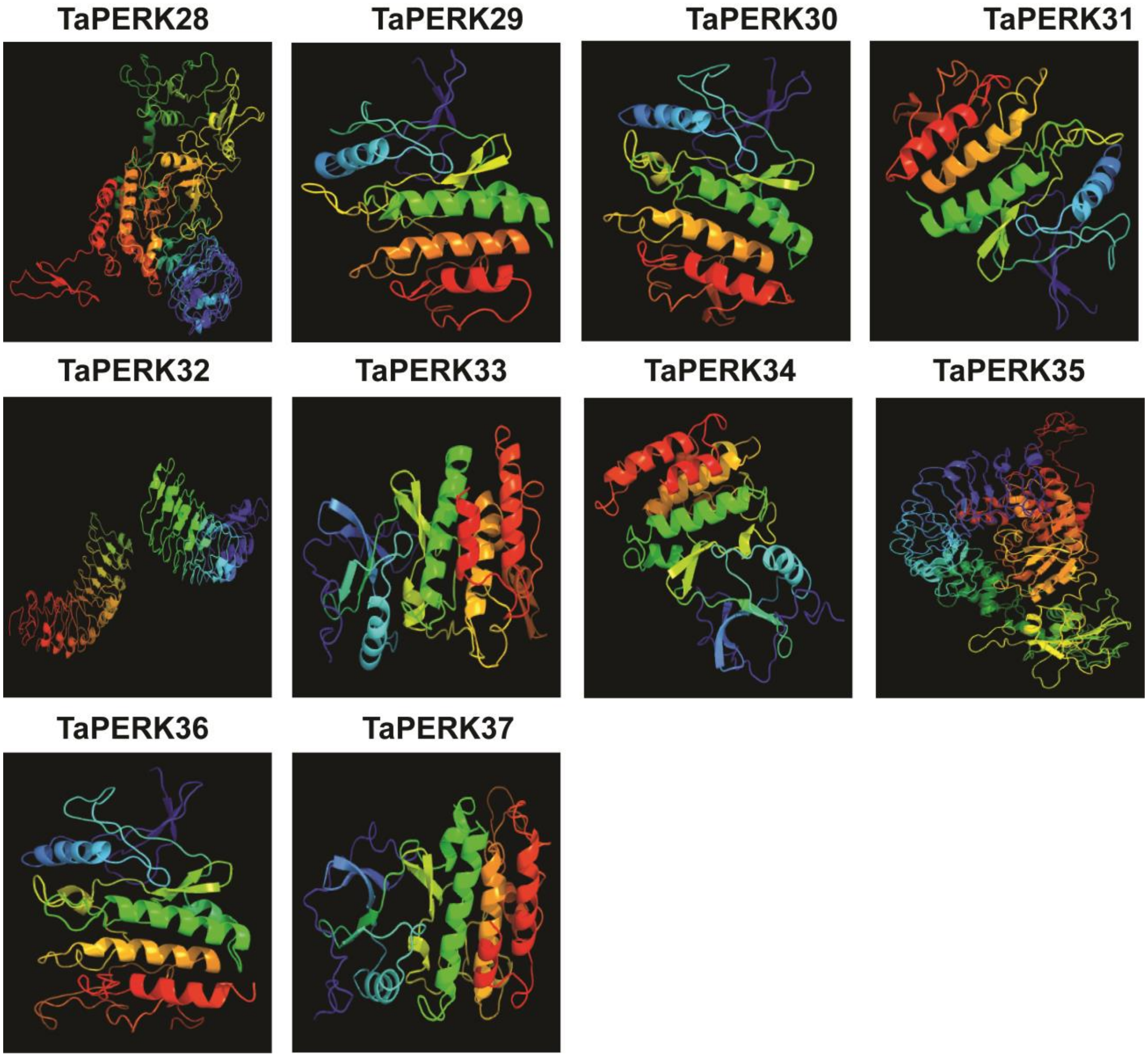

Figure S6. Alignment and 3-dimensional structure of the TaPERK protein sequences. **A.** The conserved protein tyrosine kinase domain is boxed with red color. Colored and shaded amino acids are chemically similar residues. Dashes indicate gaps introduced to maximize the alignment of the homologous region. **B.** Predicted 3D structures TaPERK proteins.

A

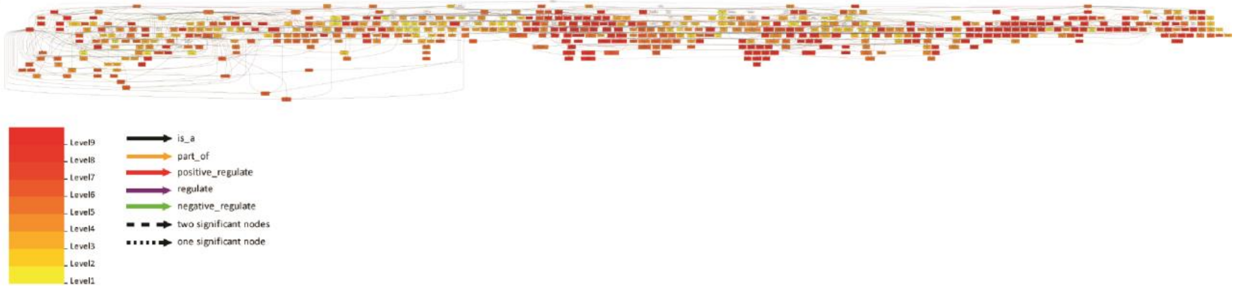

Level9
Level8
Level7
Level6
Level5
Level4
Level3
Level2
Level1
is_a
part_of
positive_regulate
regulate
negative_regulate
two significant nodes
one significant node

B

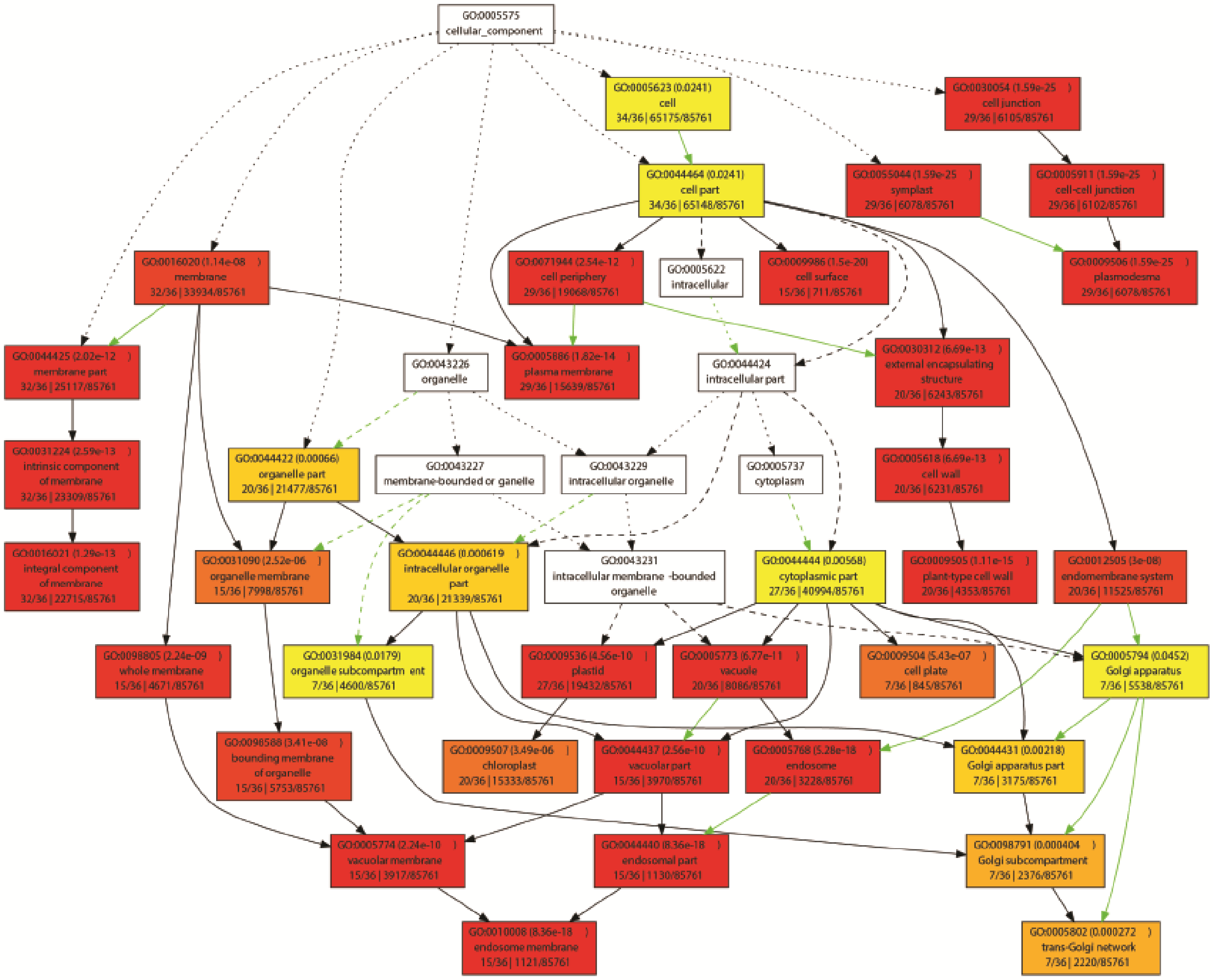

GO:0005575 cellular_component
GO:0005623 (0.0241) cell 34/36 | 65175/85761
GO:0030054 (1.59e-25) cell junction 29/36 | 6105/85761
GO:0044464 (0.0241) cell part 34/36 | 65148/85761
GO:0055044 (1.59e-25) symplast 29/36 | 6078/85761
GO:0005911 (1.59e-25) cell-cell junction 29/36 | 6102/85761
GO:0016020 (1.14e-08) membrane 32/36 | 33934/85761
GO:0071944 (2.54e-12) cell periphery 29/36 | 19068/85761
GO:0005622 intracellular
GO:0009986 (1.5e-20) cell surface 15/36 | 711/85761
GO:0009506 (1.59e-25) plasmodesma 29/36 | 6078/85761
GO:0044425 (2.02e-12) membrane part 32/36 | 25117/85761
GO:0043226 organelle
GO:0005886 (1.82e-14) plasma membrane 29/36 | 15639/85761
GO:0044424 intracellular part
GO:0030312 (6.69e-13) external encapsulating structure 20/36 | 6243/85761
GO:0031224 (2.59e-13) intrinsic component of membrane 32/36 | 23309/85761
GO:0044422 (0.00066) organelle part 20/36 | 21477/85761
GO:0043227 membrane-bounded organelle
GO:0043229 intracellular organelle
GO:0005737 cytoplasm
GO:0005618 (6.69e-13) cell wall 20/36 | 6231/85761
GO:0016021 (1.29e-13) integral component of membrane 32/36 | 22715/85761
GO:0031090 (2.52e-06) organelle membrane 15/36 | 7998/85761
GO:0044446 (0.000619) intracellular organelle part 20/36 | 21339/85761
GO:0043231 intracellular membrane -bounded organelle
GO:0044444 (0.00568) cytoplasmic part 27/36 | 40994/85761
GO:0009505 (1.11e-15) plant-type cell wall 20/36 | 4353/85761
GO:0012505 (3e-08) endomembrane system 20/36 | 11525/85761
GO:0098805 (2.24e-09) whole membrane 15/36 | 4671/85761
GO:0031984 (0.0179) organelle subcompartment 7/36 | 4600/85761
GO:0009536 (4.56e-10) plastid 27/36 | 19432/85761
GO:0005773 (6.77e-11) vacuole 20/36 | 8086/85761
GO:0009504 (5.43e-07) cell plate 7/36 | 845/85761
GO:0005794 (0.0452) Golgi apparatus 7/36 | 5538/85761
GO:0098588 (3.41e-08) bounding membrane of organelle 15/36 | 5753/85761
GO:0009507 (3.49e-06) chloroplast 20/36 | 15333/85761
GO:0044437 (2.56e-10) vacuolar part 15/36 | 3970/85761
GO:0005768 (5.28e-18) endosome 20/36 | 3228/85761
GO:0044431 (0.00218) Golgi apparatus part 7/36 | 3175/85761
GO:0005774 (2.24e-10) vacuolar membrane 15/36 | 3917/85761
GO:0044440 (8.36e-18) endosomal part 15/36 | 1130/85761
GO:0098791 (0.000404) Golgi subcompartment 7/36 | 2376/85761
GO:0010008 (8.36e-18) endosome membrane 15/36 | 1121/85761
GO:0005802 (0.000272) trans-Golgi network 7/36 | 2220/85761

C

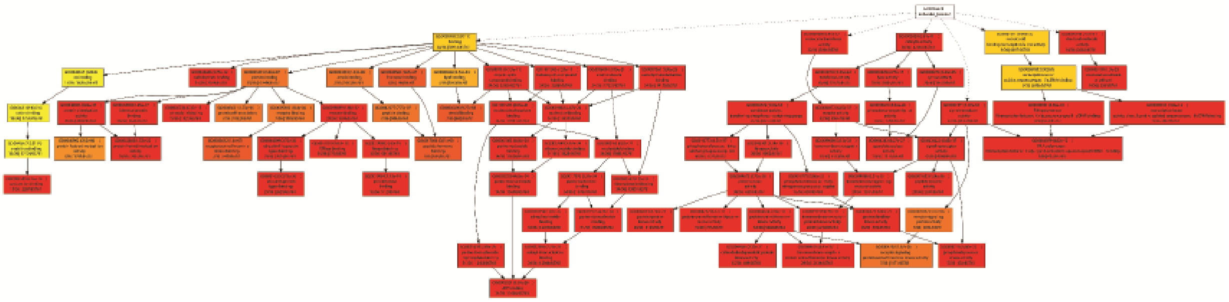

Figure S7. Gene ontology term distribution TaPERK gene family predicted using AgriGO **A.** Biological Process. **B**. Cellular component. **C.** Molecular function.

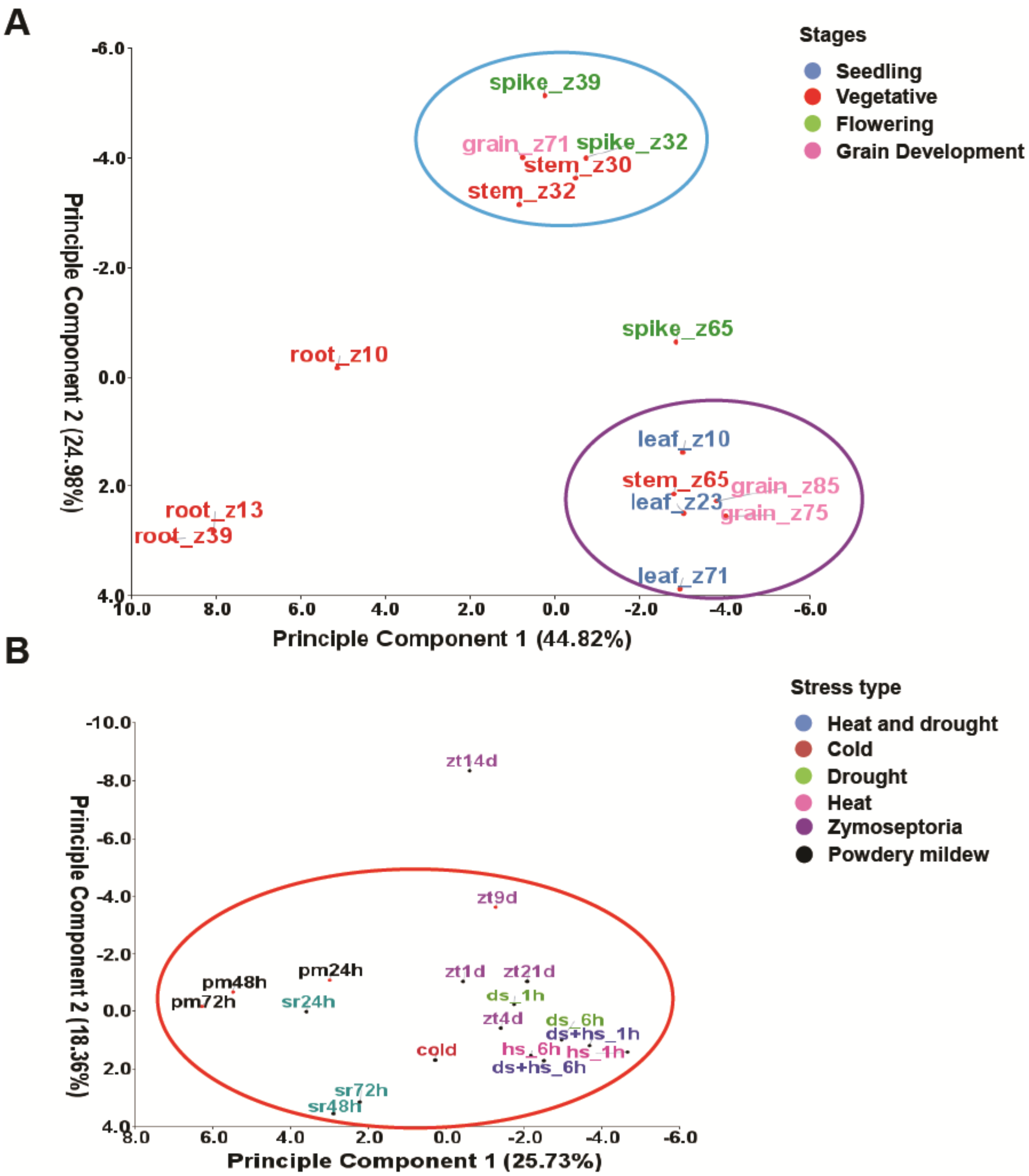

Figure S8. PCA plots displaying grouping of different (A) Developmental stages (B) Biotic and abiotic stress conditions based on the TaPERK expression pattern. DS: Drought stress, HS: Heat stress, Zt: *Zymoseptoria tritici*, PM: Powdery mildew; SR: Stripe rust, h: hour and d: days.

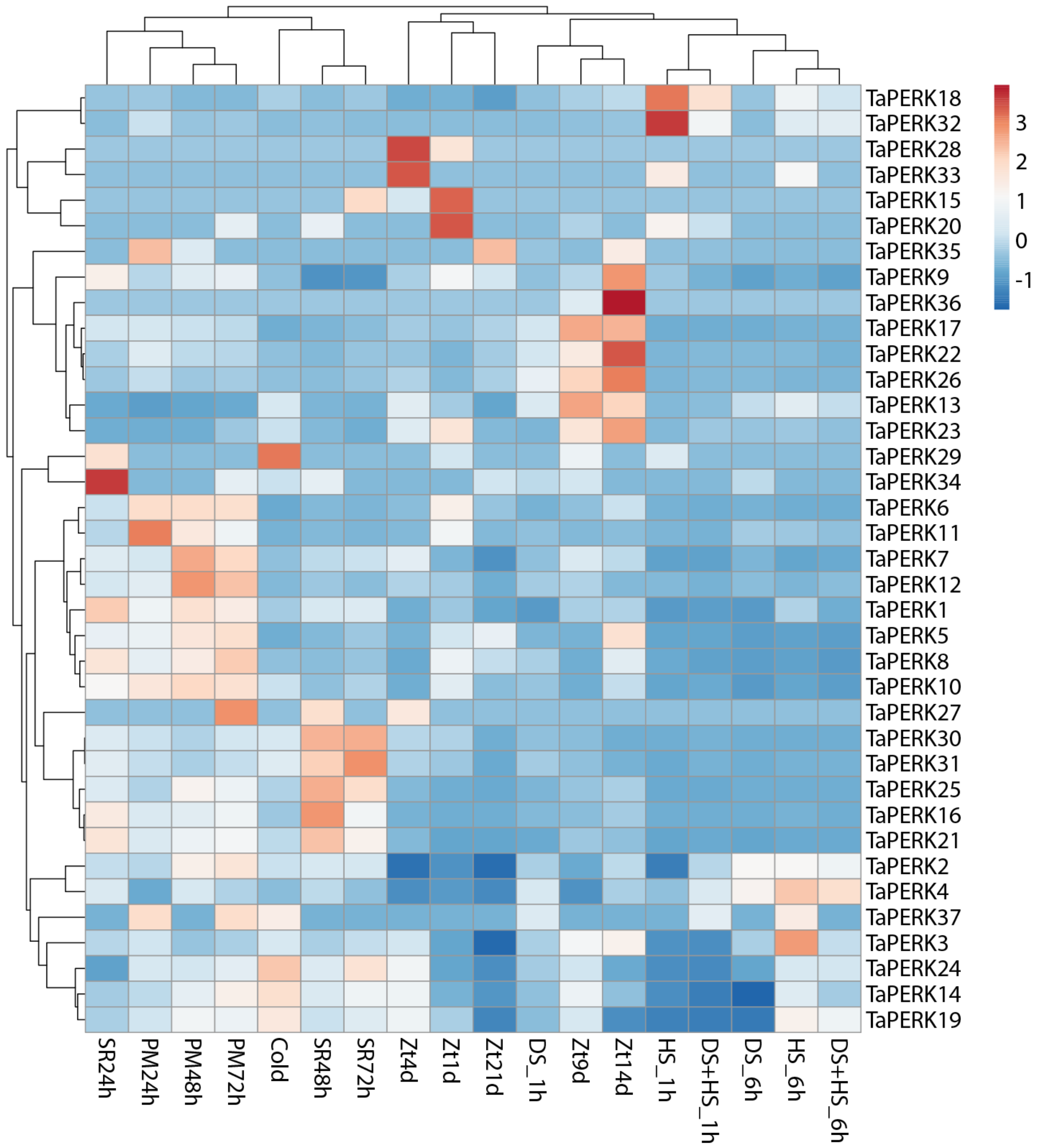

Figure S9. Heatmaps representing the expression pattern of TaPERK genes in different stress conditions. TPM values were directly used to construct the heatmaps. DS: Drought stress, HS: Heat stress, Zt: *Zymoseptoria tritici*, PM: Powdery mildew; SR: Stripe rust, h: hour and d: days.
