# Supplementary figures and images for: Genome-Wide Analysis and Characterization of the Proline-Rich Extensin-like Receptor Kinases (PERKs) Gene Family Reveals Their Role in Different Developmental Stages and Stress Conditions in Wheat (Triticum aestivum L.)
Source: Plants (Basel). 2022 Feb 11;11(4):496. doi: 10.3390/plants11040496 (PMC8880425; doi:10.3390/plants11040496)

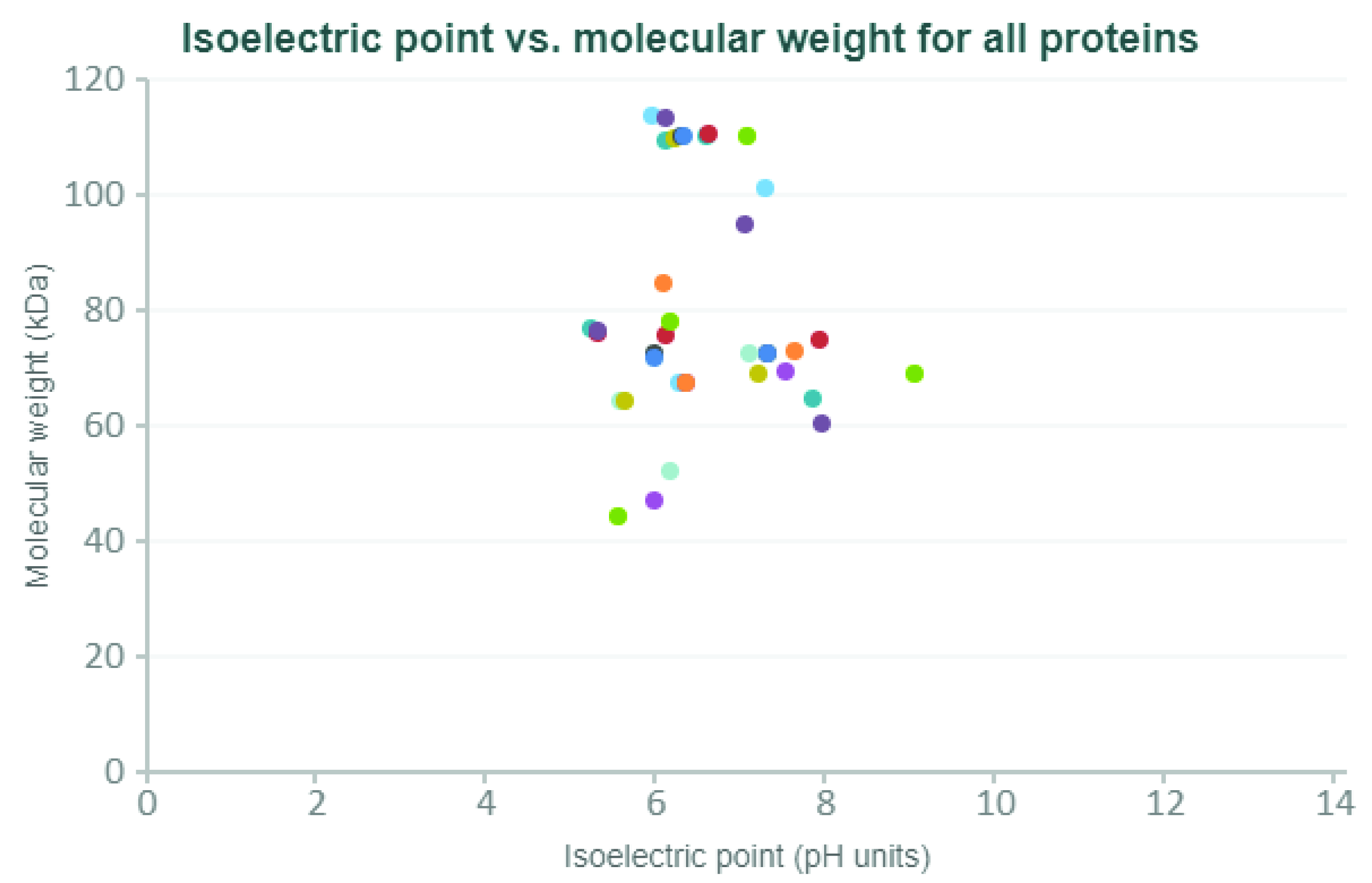

Supplement: Supplementary file 1 [file plants-11-00496-s001.zip › sup/Figure S1.tif]

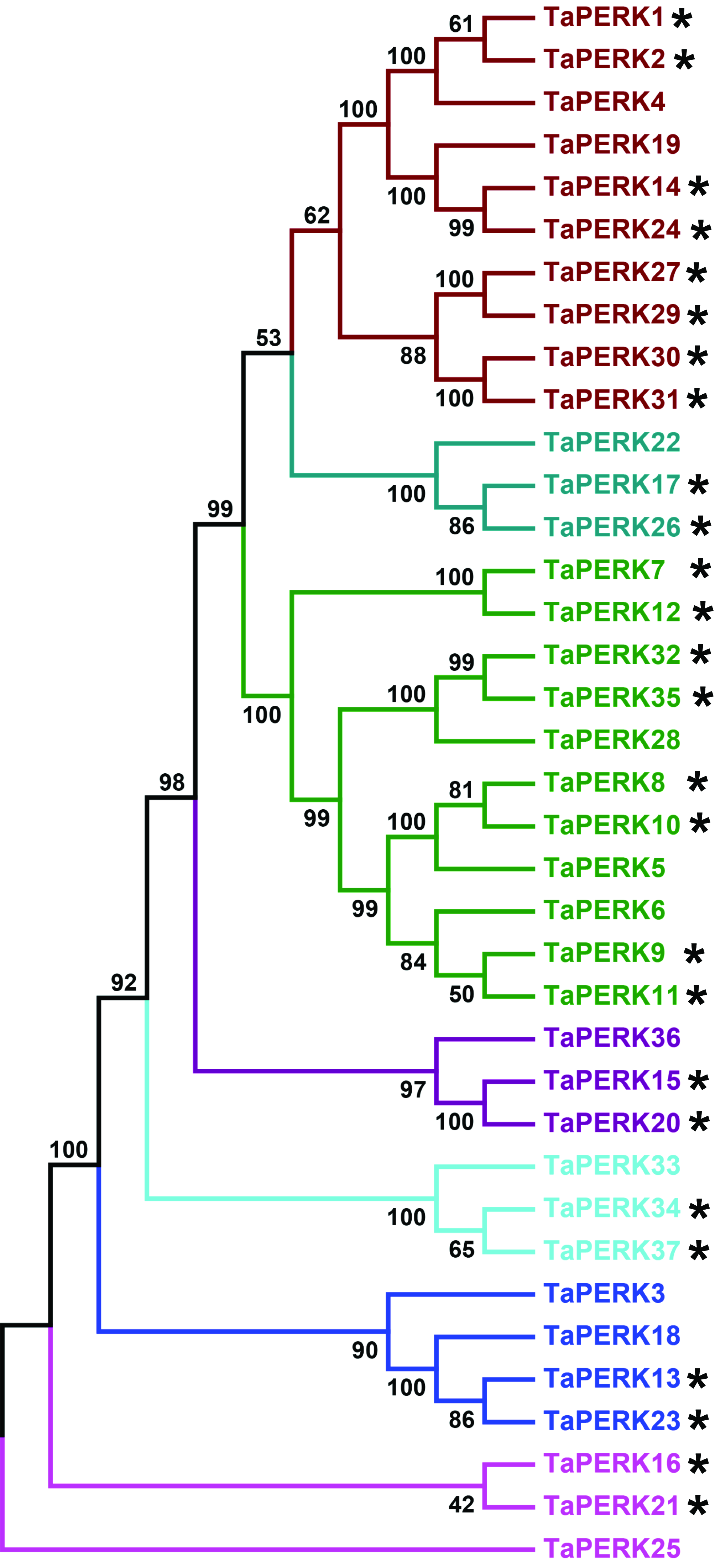

Supplement: Supplementary file 1 [file plants-11-00496-s001.zip › sup/Figure S3.tif]

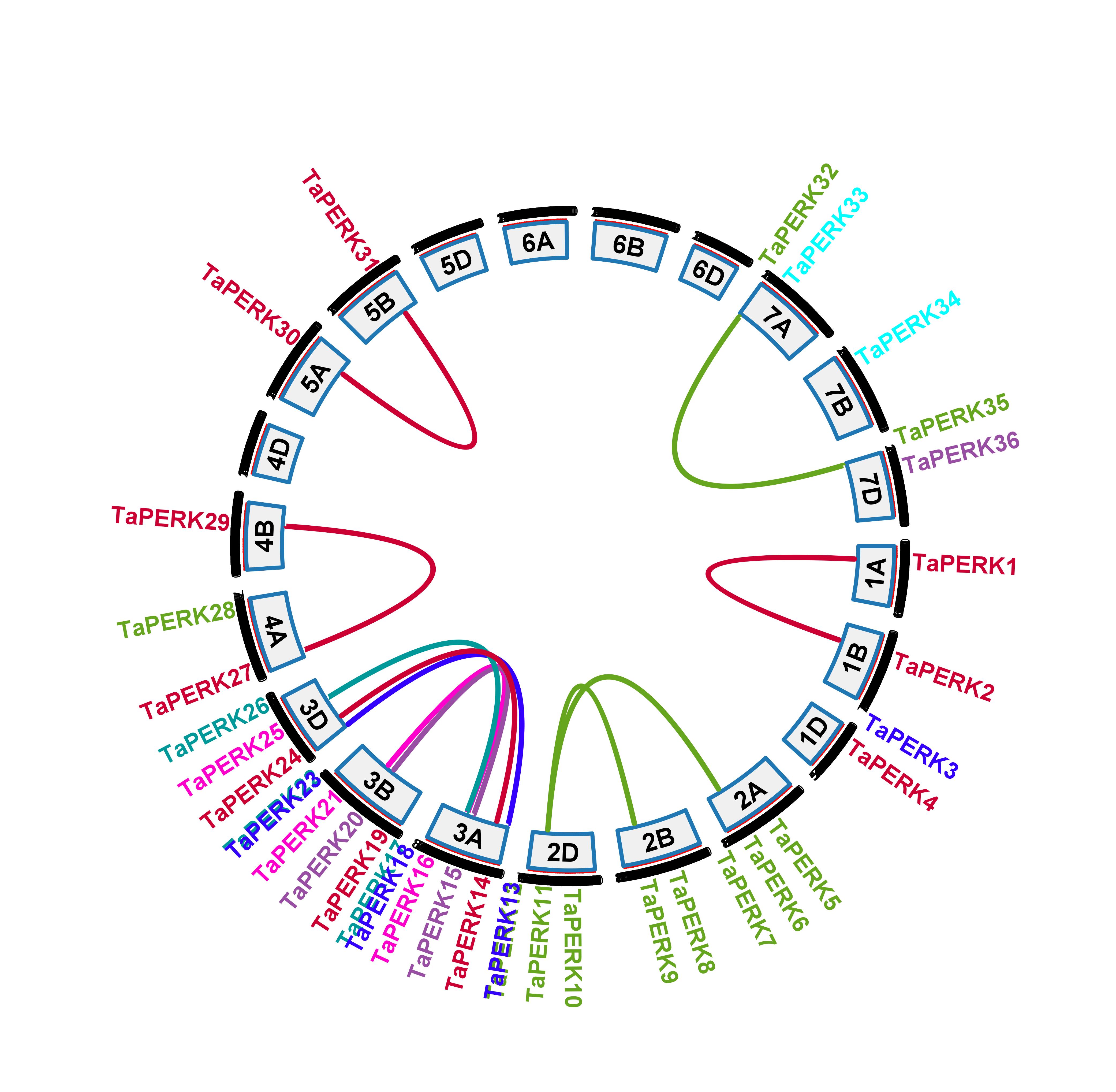

Supplement: Supplementary file 1 [file plants-11-00496-s001.zip › sup/Figure S4.jpg]

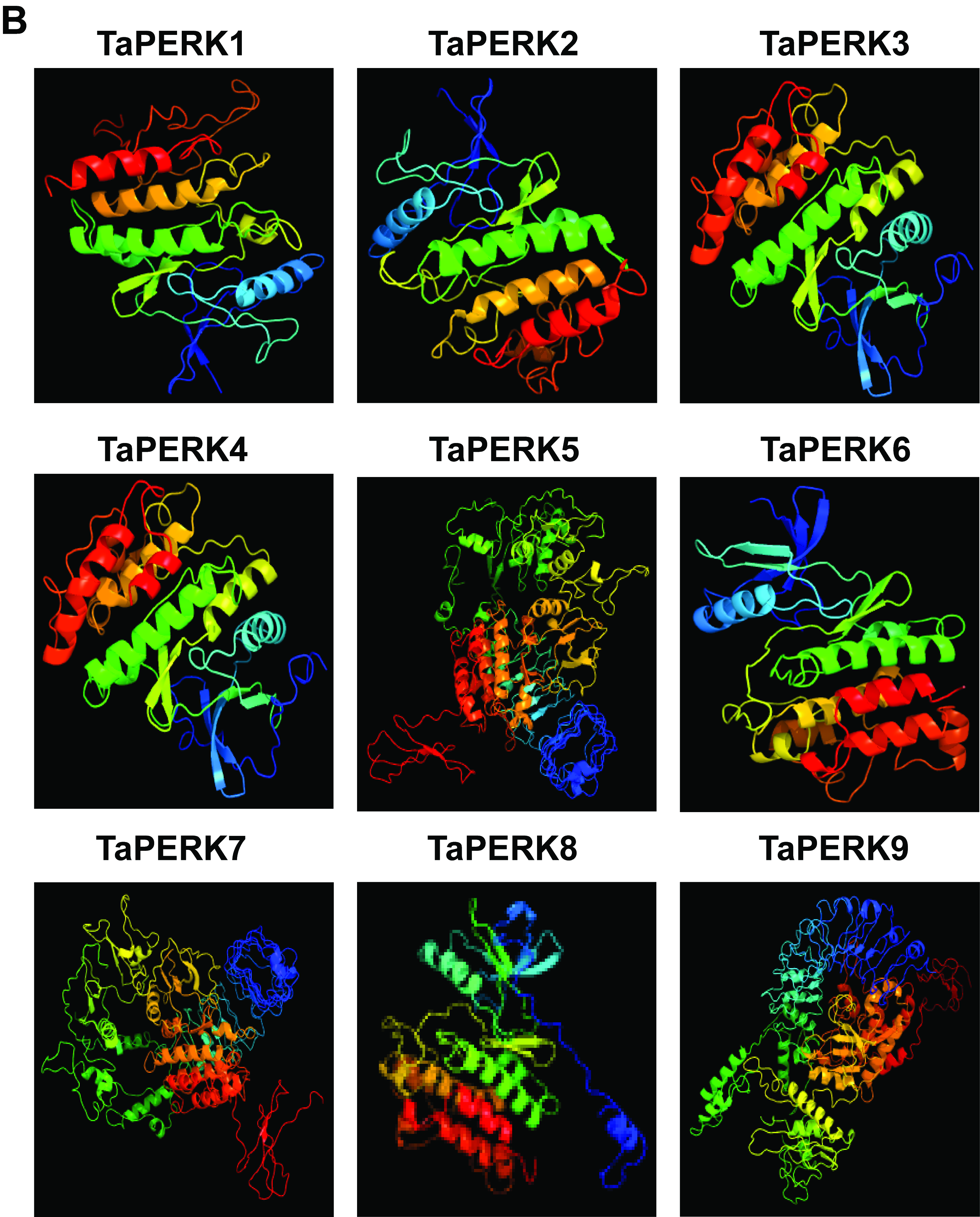

Supplement: Supplementary file 1 [file plants-11-00496-s001.zip › sup/Figure S6-B1.tif]

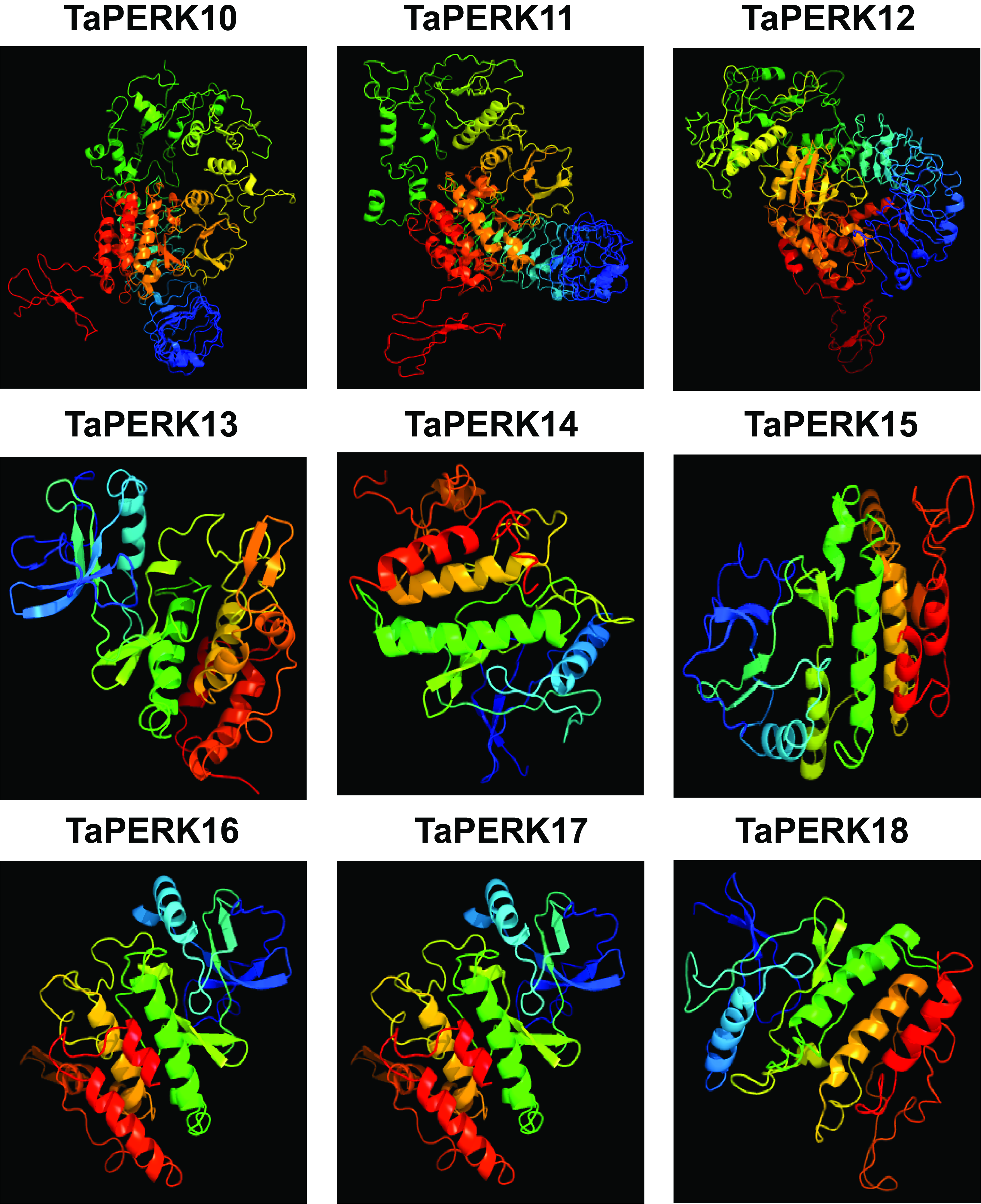

Supplement: Supplementary file 1 [file plants-11-00496-s001.zip › sup/Figure S6-B2.tif]

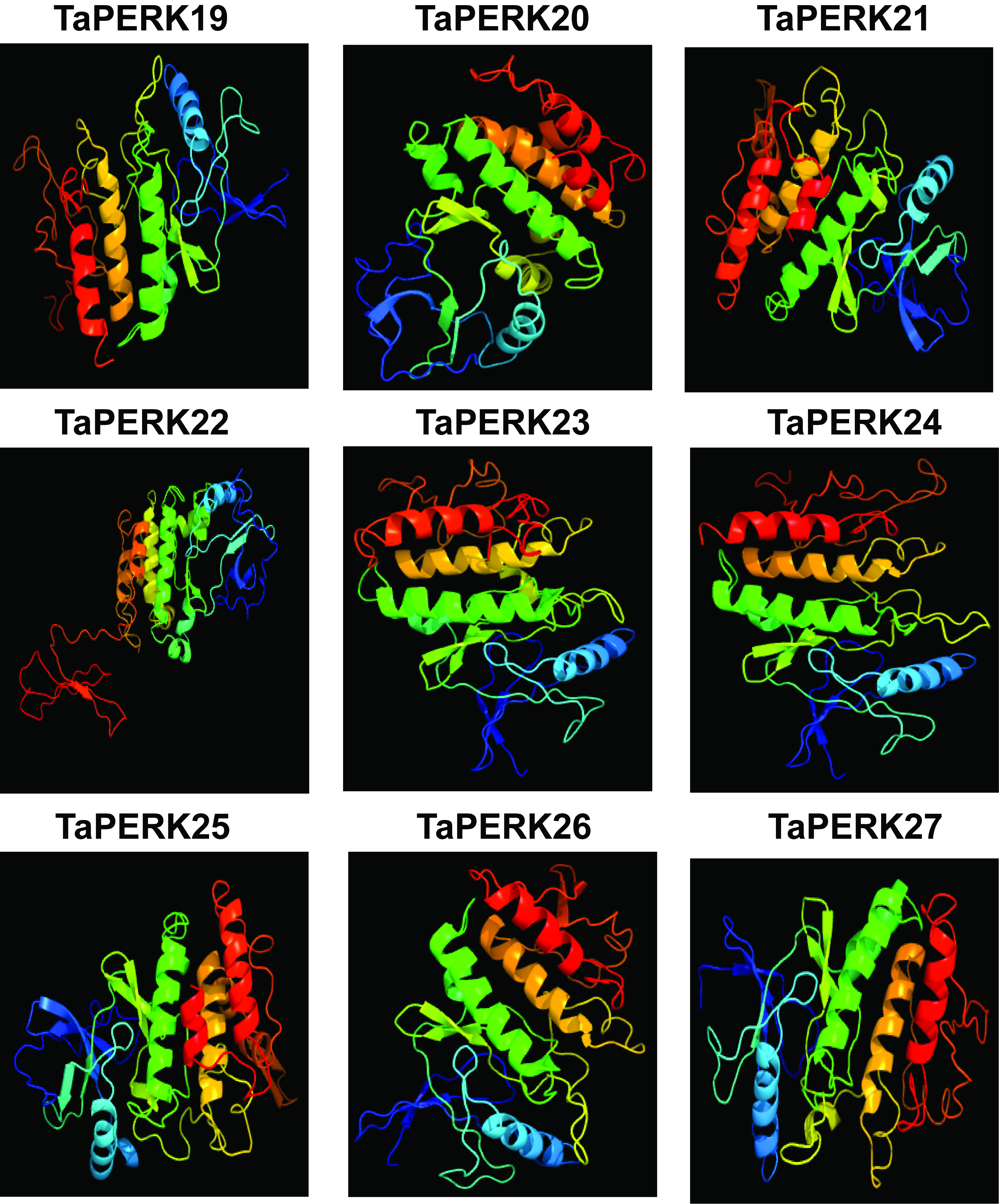

Supplement: Supplementary file 1 [file plants-11-00496-s001.zip › sup/Figure S6-B3.tif]

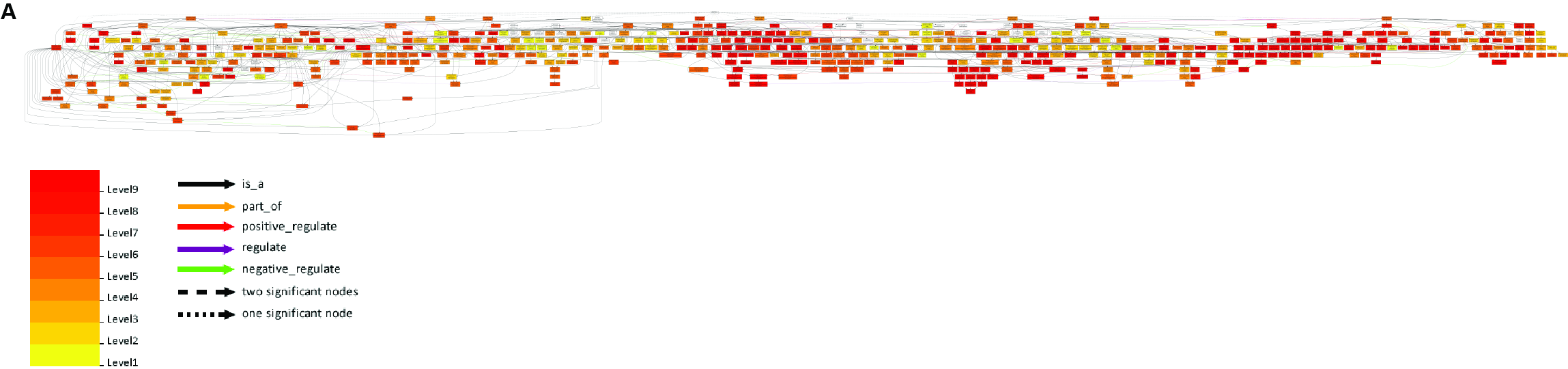

Supplement: Supplementary file 1 [file plants-11-00496-s001.zip › sup/Figure S7A.tif]

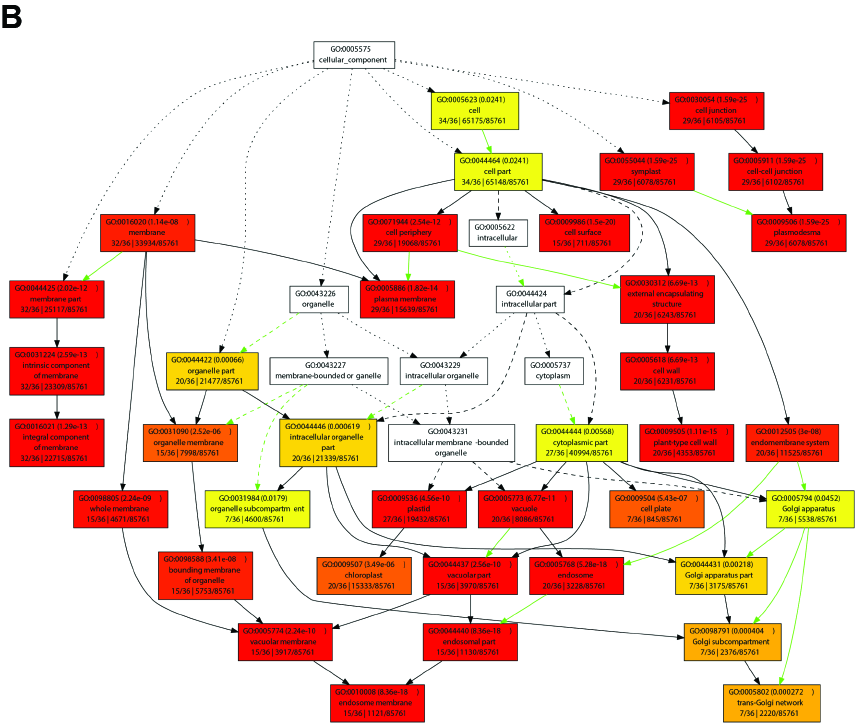

Supplement: Supplementary file 1 [file plants-11-00496-s001.zip › sup/Figure S7B.tif]

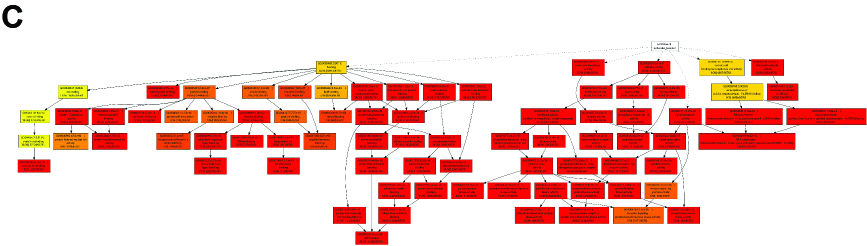

Supplement: Supplementary file 1 [file plants-11-00496-s001.zip › sup/Figure S7C.tif]

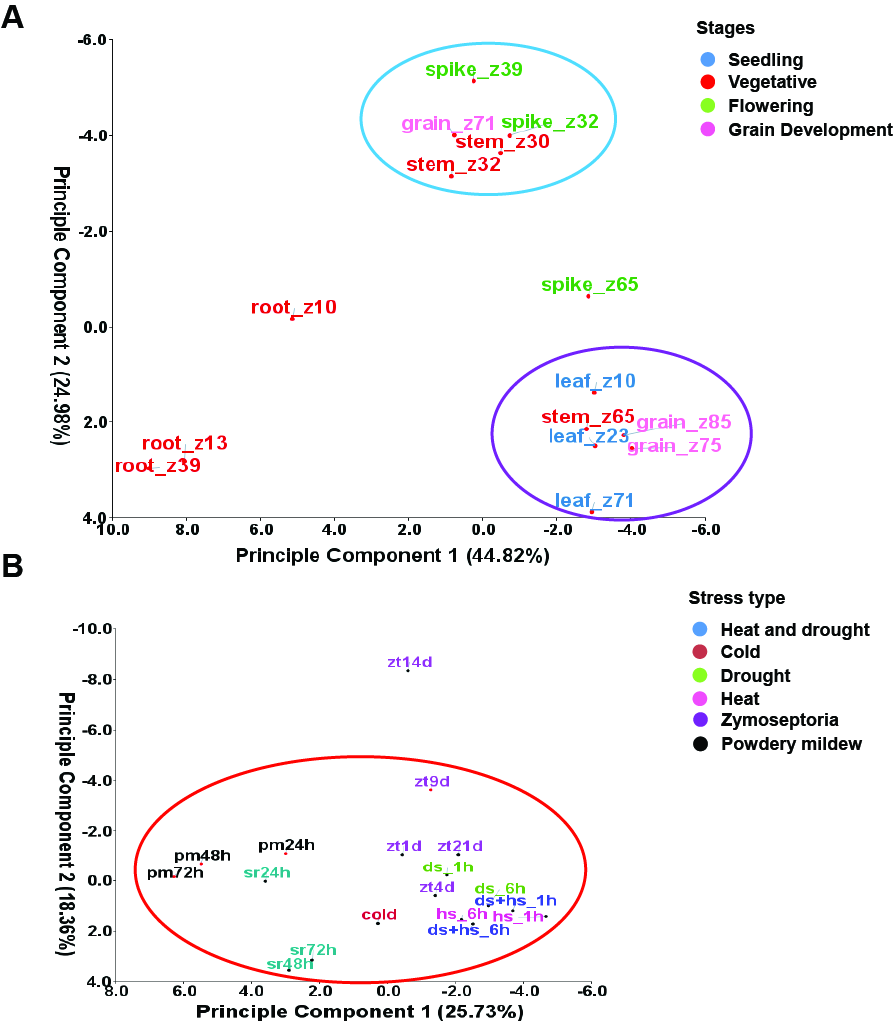

Supplement: Supplementary file 1 [file plants-11-00496-s001.zip › sup/Figure S8.tif]

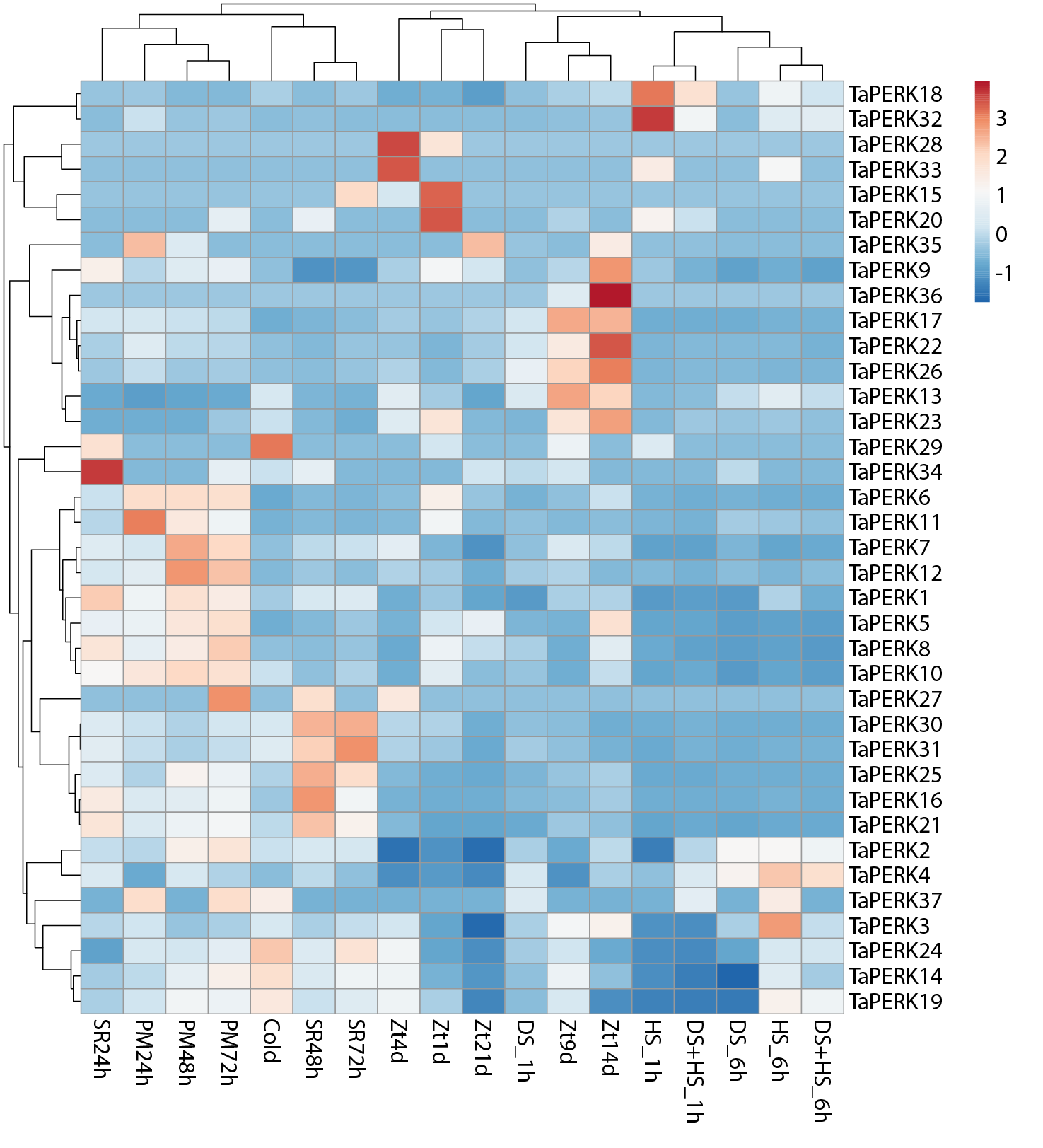

Supplement: Supplementary file 1 [file plants-11-00496-s001.zip › sup/Figure S9.tif]
